# The Quorum Sensing Auto-Inducer 2 (AI-2) Stimulates Nitrogen Fixation and Favors Ethanol Production over Biomass Accumulation in *Zymomonas mobilis*

**DOI:** 10.3390/ijms22115628

**Published:** 2021-05-26

**Authors:** Valquíria Campos Alencar, Juliana de Fátima dos Santos Silva, Renata Ozelami Vilas Boas, Vinícius Manganaro Farnézio, Yara N. L. F. de Maria, David Aciole Barbosa, Alex Tramontin Almeida, Emanuel Maltempi de Souza, Marcelo Müller-Santos, Daniela L. Jabes, Fabiano B. Menegidio, Regina Costa de Oliveira, Tiago Rodrigues, Ivarne Luis dos Santos Tersariol, Adrian R. Walmsley, Luiz R. Nunes

**Affiliations:** 1 Centro de Ciências Naturais e Humanas, Universidade Federal do ABC (UFABC), Alameda da Universidade, s/n, São Bernardo do Campo 09606-045, SP, Brazil; valquiria.alencar@ufabc.edu.br (V.C.A.); silva.juliana@ufabc.edu.br (J.d.F.d.S.S.); v.manganaro@ufabc.edu.br (V.M.F.); tiago.rodrigues@ufabc.edu.br (T.R.); 2 Núcleo Integrado de Biotecnologia, Universidade de Mogi das Cruzes (UMC), Av. Dr. Cândido Xavier de Almeida Souza, 200, Mogi das Cruzes 08780-911, SP, Brazil; 11141100788@alunos.umc.br (R.O.V.B.); 11152500497@alunos.umc.br (Y.N.L.F.d.M.); 11121503188@alunos.umc.br (D.A.B.); danielajabes@umc.br (D.L.J.); fabianomenegidio@umc.br (F.B.M.); reginaco@umc.br (R.C.d.O.); 3 Setor de Ciências Biológicas-Departamento de Bioquímica e Biologia Molecular, Universidade Federal do Paraná (UFPR), Rua Cel. Francisco H. dos Santos, 100, Curitiba 81531-980, PR, Brazil; alex.ta@ufpr.br (A.T.A.); souzaem@ufpr.br (E.M.d.S.); marcelomuller@ufpr.br (M.M.-S.); 4 Departamento de Bioquímica, Universidade Federal de São Paulo (UNIFESP), Rua Três de Maio, 100, São Paulo 04044-020, SP, Brazil; ivarne.tersariol@unifesp.br; 5 Department of Biosciences, Durham University, South Road, Durham DH1 3LE, UK; a.r.walmsley@durham.ac.uk

**Keywords:** *Zymomonas mobilis*, quorum sensing, AI-2, N_2_ fixation, ethanol production, transcriptome

## Abstract

Autoinducer 2 (or AI-2) is one of the molecules used by bacteria to trigger the Quorum Sensing (QS) response, which activates expression of genes involved in a series of alternative mechanisms, when cells reach high population densities (including bioluminescence, motility, biofilm formation, stress resistance, and production of public goods, or pathogenicity factors, among others). Contrary to most autoinducers, AI-2 can induce QS responses in both Gram-negative and Gram-positive bacteria, and has been suggested to constitute a trans-specific system of bacterial communication, capable of affecting even bacteria that cannot produce this autoinducer. In this work, we demonstrate that the ethanologenic Gram-negative bacterium *Zymomonas mobilis* (a non-AI-2 producer) responds to exogenous AI-2 by modulating expression of genes involved in mechanisms typically associated with QS in other bacteria, such as motility, DNA repair, and nitrogen fixation. Interestingly, the metabolism of AI-2-induced *Z. mobilis* cells seems to favor ethanol production over biomass accumulation, probably as an adaptation to the high-energy demand of N_2_ fixation. This opens the possibility of employing AI-2 during the industrial production of second-generation ethanol, as a way to boost N_2_ fixation by these bacteria, which could reduce costs associated with the use of nitrogen-based fertilizers, without compromising ethanol production in industrial plants.

## 1. Introduction

Quorum sensing (QS) is a chemical communication process employed by bacteria to assess the population density in their surrounding environment and synchronize their behavior on a community scale [1,2]. This communication is mediated by small signaling molecules called auto-inducers (AI), which are constitutively produced by the cells, during all stages of development. Thus, when the population reaches higher cellular densities, the concentration of AI molecules in the environment becomes critical, triggering a coordinated response, in which all cells reprogram their gene expression patterns simultaneously, activating genes involved with a series of alternative activities, such as bioluminescence, motility, biofilm production and, in many cases, secretion of public goods and/or pathogenicity factors [3]. In this sense, the QS response involves expression of phenotypes that would be too costly and counterproductive, if executed by only a few cells, but result in efficient responses, when expressed in a coordinated manner, by the entire population [3].

Gram-positive bacteria generally use oligopeptides as QS autoinducers, while Gram-negative bacteria use acyl-homoserine-lactone derivatives (AHLs). Collectively, these molecules are called type 1 auto-inducers, or AI-1 [2,3]. In addition, many bacteria can use an alternative QS signaling molecule, known as a type 2 auto-inducer (AI-2) [4]. The basic structure of AI-2 is a 4,5-dihydroxy-2,3-pentanedione (DPD), which cyclizes spontaneously to form a series of interconverting derivatives, which coexist in a dynamic balance [3]. AI-2 is synthesized during the reaction mediated by the enzyme LuxS, as a byproduct of the active methyl cycle, which transfers methyl groups to various substrates, during major biosynthetic pathways of bacterial metabolism [5]. Contrary to the oligopeptide and AHL autoinducers (which display species-specific structure and activity), AI-2 can trigger QS responses in various bacteria (both Gram-negative and Gram-positive), and has been suggested to constitute a trans-specific system of bacterial communication, capable of affecting the composition, and behavior of complex bacterial communities [4,5,6].

In this manuscript, we show that the Gram-negative bacterium *Zymomonas mobilis* (a non-AI-2 producer) responds to the presence of this autoinducer with significant alterations in its growth rate and gene expression profile. Such transcriptional reprogramming affects genes associated with various mechanisms typically associated with QS responses in other microorganisms, including motility, DNA repair, and nitrogen fixation, among others. *Z. mobilis* is a diazotrophic ethanologenic bacterium, widely employed in the production of second-generation ethanol and other industrial bioconversion processes [7]. Interestingly, previous studies have suggested that activation of the bacterial N_2_-fixing mechanism could result in significant savings for the bioethanol industry, allowing atmospheric N_2_ gas to be used as a nitrogen source, in substitution to industrial fertilizers [8,9]. Moreover, N_2_ fixation in *Z. mobilis* seems to be coupled with reduced glucose-to-biomass and increased glucose-to-ethanol turnover ratios, suggesting that AI-2 may assist in the development of improved mechanisms for bioethanol production.

## 2. Results

### 2.1. Z. mobilis Response to Increasing Concentrations of AI-2

**Figure 1:**
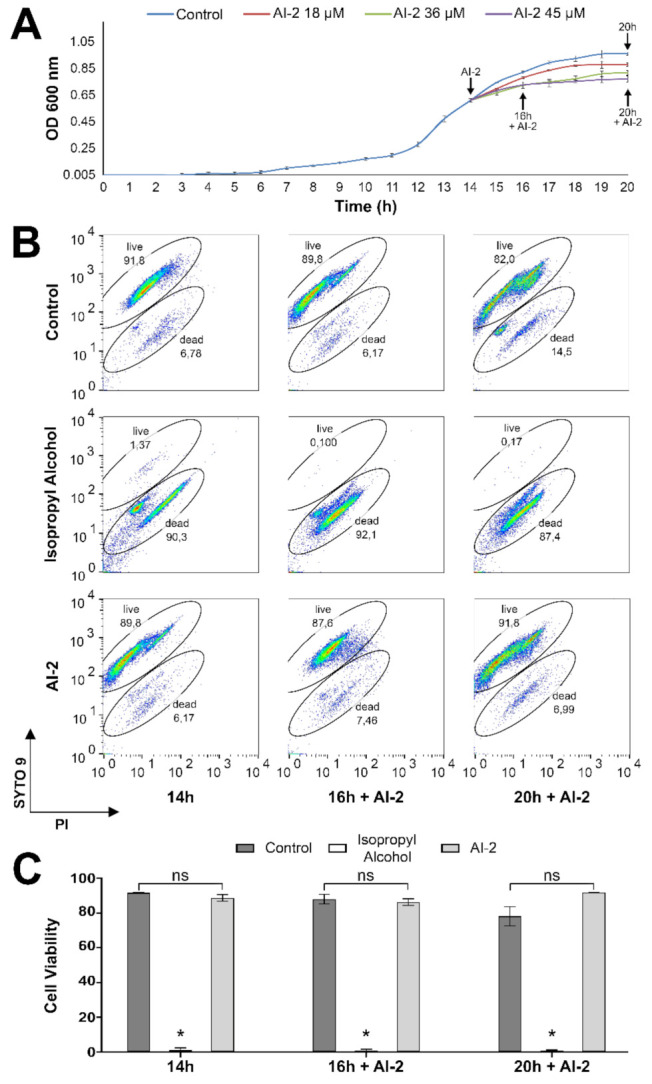
Effects of AI-2 on *Z. mobilis* growth rate and viability. (**A**) An initial *Z. mobilis* culture was established, in 5 mL of liquid MM, from an isolated colony, grown in solid MM. This culture was incubated at 30 °C (without shaking) for 24 h, when aliquots were taken and used to inoculate a flask containing 80 mL of liquid MM, to an OD_600_ = 0.05. This flask was then incubated under the same conditions and cell growth was monitored hourly by OD_600_ readings. When this culture reached mid-exponential growth phase (at t = 14 h), it was subdivided into four vials of 20 mL each. AI-2 was then added to these cultures, at different concentrations (0, 18, 36, and 45 µM). Next, the cultures were re-incubated, as described above, and the OD_600_ continued to be monitored, until all reached stationary phase. OD_600_ readings shown in the graph represent the averages ± SEM from three independent experiments. Aliquots (2 mL) were taken for RNA extraction at the times indicated by the arrows, namely: (i) t = 14 h (before addition of AI-2); (ii) t = 16 h + AI-2 (treated with 45 µM AI- 2); (iii) t = 20 h + AI-2 (treated with 45 µM AI- 2); and (iv) t = 20 h (not treated with AI-2). See text for further details. (**B**) Representative dot plots showing SYTO 9 and PI fluorescence obtained by flow cytometry in cells grown in the absence of AI-2 (control), treated with 70% isopropyl alcohol (positive control), and treated with 45 µM AI-2, at the same incubation times highlighted in panel (**A**). (**C**) Quantification of percentages of live and dead cells. The results are presented as the mean ± SEM of three independent experiments and * represents statistically significant differences in relation to the respective controls (AI-2 untreated cultures), after a two-tailed *t*-test, using *p* < 0.05 as a threshold; ns represent statistically non-significant differences.

To check *Z. mobilis* response to AI-2, liquid cultures were established in minimal medium (MM), as described in Materials and Methods. When cells reached exponential growth phase, AI-2 was added to each culture, at different concentrations (0, 18, 36, and 45 μM), and cell growth continued to be monitored by OD_600_ measurements, until all cultures reached stationary phase. As observed in Figure 1A, cells grown in MM showed a long period of adaptive growth (lag phase), reaching exponential phase only after 12–14 h of incubation. In the absence of AI-2, the cultures showed decreased growth rate after 16 h and stationary phase is established after 19 h. However, the decrease in exponential growth rate and the consequent establishment of stationary phase are induced earlier in *Z. mobilis* cultures (in a concentration-dependent manner) by the presence of AI-2. For example, cultures subjected to 45 μM AI-2 showed a pronounced reduction in growth rate during the first hour of incubation, entering stationary phase at 16 h (Figure 1A).

To verify whether AI-2 exhibits toxicity against *Z. mobilis* cells, a flow cytometry assay, employing the LIVE/DEAD^®^ BacLigh^®^ Bacterial Viability Kit, was conducted at the same experimental conditions (see Materials and Methods, for details). This assay uses two fluorochromes that bind to nucleic acids, SYTO 9, and propidium iodide (PI). SYTO 9 is a cell-permeant green fluorescent dye that labels live and dead bacteria, whereas PI, as a cell-impermeant red fluorescent dye, only enters into bacterial cells with altered membrane permeability (damaged/dead). The binding of PI to nucleic acids results in an apparent diminished green fluorescence of SYTO 9, due to quantum yielding, when both fluorochromes are inside the cells, allowing visual separation of viable cells (containing intact membranes), from membrane-compromised/dead bacteria [10]. In this assay, isopropyl alcohol was used to promote a positive control for cell damage/death, as indicated in the manufacturer’s instructions (see Materials and Methods). Based on the position of cell populations in both untreated controls and alcohol-treated experiments, a gate-based strategy was used to distinguish between live and damaged/dead cells. The representative dot plots presented in Figure 1B show that AI-2 did not alter the viability of *Z. mobilis* cells, when AI-2-treated cells were compared to the negative control (absence of AI-2). The quantification of the percentages of live and dead/damaged cells is presented in Figure 1C, considering a total of three independent replicas. Thus, these data show that the decreased growth rate induced by AI-2 does not seem to be due to AI-2 toxicity.

Global transcriptome analyses were next conducted to characterize the main metabolic traits of *Z. mobilis* that were affected by the presence of AI-2, using RNA-seq libraries (see Materials and Methods for details). Thus, aliquots were taken from cultures treated with 45 μM AI-2 for periods of 2 and 6 h (t = 16 h + AI-2 and t = 20 h + AI-2, respectively, as shown in Figure 1). The transcriptomes of cells harvested in these two moments were then compared to the transcriptome of cells not treated with AI-2, in exponential growth phase, harvested at time t = 14 h (immediately before the addition of AI-2). However, since cells involved in these analyses were at different growth phases (exponential phase at t = 14h, or stationary phase at t = 16 h + AI-2 and t = 20 + AI-2), cells were also harvested from a culture not treated with AI-2, at time t = 20 h. These cells also showed a growth pattern compatible with stationary phase, allowing us to differentiate gene expression changes involved with entry into stationary phase from those derived from the presence of AI-2, when comparing all available transcriptomes (t = 20 h, t = 16 h + AI-2 and t = 20 h + AI-2) against the same reference (t = 14 h).

### 2.2. Characterization of Gene Modulation Patterns in Z. mobilis, in Response to Cell Growth and the Presence of AI-2

**Figure 2:**
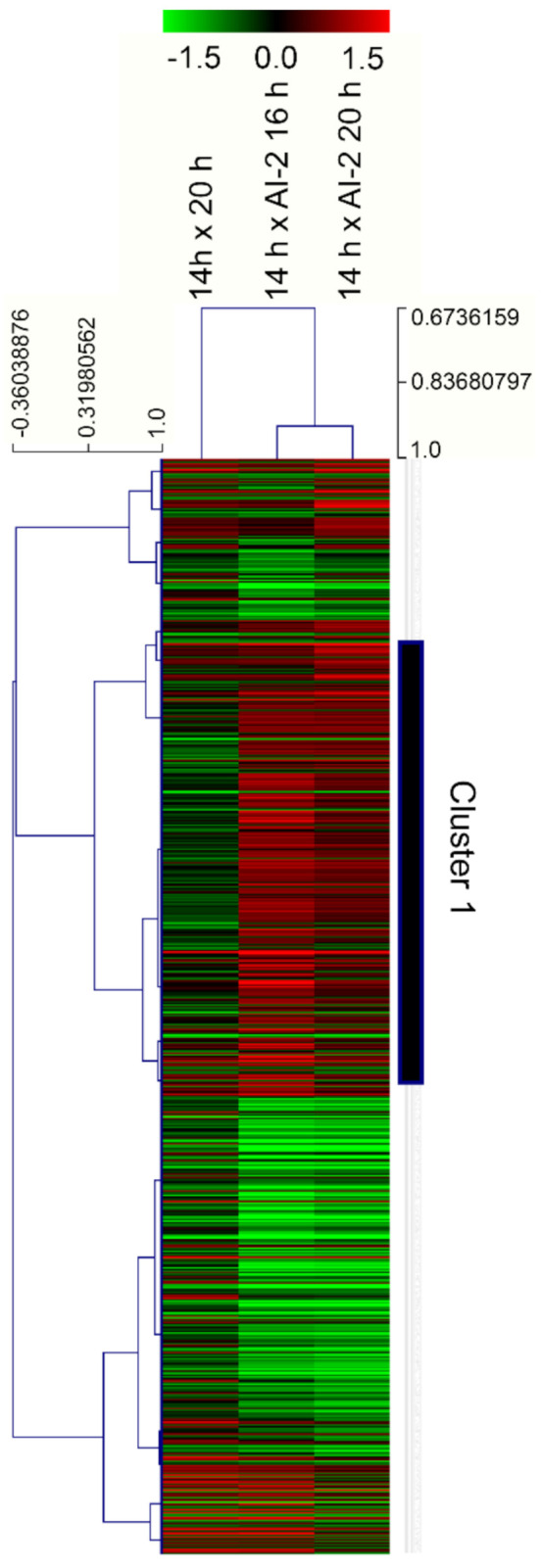
Gene expression patterns of *Z. mobilis* cultures, in response to the presence of AI-2 and different growth phases. The gene expression patterns shown above were obtained through RNA-seq experiments, employing samples obtained from the different timepoints depicted in Figure 1. RNA obtained from cells grown for t = 14 h (exponential growth phase) was used as a common reference for transcriptome comparisons with cells grown for t = 20 h (stationary phase), as well as cells induced to early stationary phase, due to the presence of 45 µM AI-2 (t = 16 h + AI-2 and t = 20 h + AI-2). The figure shows the average values for each of the 724 genes identified as modulated during three relative comparisons [log_2_(t = 20 h /t = 14 h), Log_2_(t = 16 h + AI-2/t = 14 h) and log_2_(t = 20 h + AI-2/t = 14 h)] (see Materials and Methods for details). The genes and experimental conditions were subjected to a hierarchical clustering algorithm and visualized with the aid of TMEV [11]. Genes highlighted in Cluster 1 show an expression pattern that differentiates cells treated with AI-2 from those that spontaneously entered stationary phase (see text for details).

Total RNA was extracted from the bacteria harvested at the abovementioned timepoints and used to construct RNA-seq libraries. All transcriptome comparisons were made using the transcriptome of untreated cells, at mid-exponential growth phase (t = 14 h) as a common reference, resulting in three pairs of comparisons: (i) t = 20 h × t = 14 h; (ii) t = 16 h + AI-2 × t = 14 h and (iii) t = 20 h + AI-2 × t = 14 h. As highlighted in Materials and Methods, all analyses were conducted with results obtained from three independent cultures, obtained for each experimental condition. The complete dataset derived from the RNA-seq analyses can be found at the Open Science Framework (OSF) repository (https://osf.io/rs8pu/, accessed on 25 April 2021). Differentially expressed genes were identified by ANOVA, followed by a Benjamini–Hochberg (BH) post-test, using *q* ≤ 0.01 as the limit for statistical reliability. Additionally, only genes displaying absolute log_2_ modulation ≥ 0.6, in at least one experimental timepoint, were considered for further analyses (this threshold is equivalent to >50% up, or down-regulation, in comparisons with gene expression values at t = 14 h). These conditions led to the identification of 724 genes, whose modulation patterns are shown in Figure 2 (details concerning gene identities are provided in the Supplementary Materials File S1). To verify the overall reliability of the RNA-seq results, a set of 20 genes had their relative expression ratios confirmed by qPCR and a qualitative comparison of the results obtained by these two methodologies indicate that ~80% of them were equally identified as either induced, or repressed, by these two independent approaches (see Supplementary Materials File S2).

As seen in Figure 2, significant gene modulation can be verified in *Z. mobilis* cells after 2 h of treatment with AI-2 (t = 16 h + AI-2 × t = 14 h) and such pattern remains practically unchanged, even after 6 h of induction (t = 20 h + AI-2 × t = 14 h). In fact, most of these modulations are likely associated with bacterial entry into stationary phase (which seems to be early induced by AI-2), as they can also be verified in control cells (not treated with the autoinducer), which have spontaneously entered stationary phase, at t = 20 h (t = 20 h × t = 14 h). However, when these three pairs of comparisons are viewed with the aid of a hierarchical clustering algorithm, it is possible to identify a series of genes that are specifically modulated only in AI-2-treated cells, confirming that this signaling molecule induces a significant number of transcriptional changes in *Z. mobilis,* which are not related with entry into stationary phase (see cluster 1 in Figure 2).

### 2.3. Metabolic and Structural Alterations Verified in Z. mobilis in Response to Cell Growth and the Presence of AI-2

**Figure 3:**
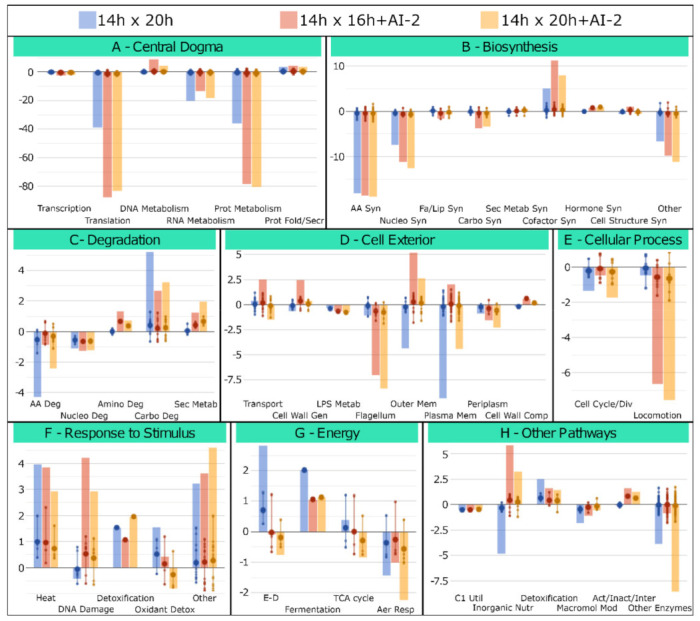
Functional distribution of differentially modulated genes. The OMICS Dashboard tool, available through the Pathway Tools package, was used to distribute the 724 genes shown in Figure 2 into the 8 main functional/structural systems/subsystems defined in the ZM4 Pathway Genome Database (PGDB), available at BioCyc (panels **A** to **H**) (see text, for details). Total modulation of the different categories is shown by the sum of relative expression (Log2 ratios) for all genes present in each system/subsystem. Details regarding each gene present in each functional/structural category can be found in Supplementary Materials File S3.

A prospective evaluation of the *Z. mobilis* functional/structural response to AI-2, at different growth conditions, was obtained by comparing the expression profile of the 724 genes observed in Figure 2 with the OMICS Dashboard tool, available through the Pathway Tools software (https://arxiv.org/abs/1510.03964, accessed on 20 June 2020). Thus, genes were initially distributed into their respective Gene Ontology (GO) categories [12] and grouped in specific structural/functional systems/subsystems, according to the *Z. mobilis* ZM4 Pathway/Genome Database (PGDB), available at the BioCyc repository (https://biocyc.org/, accessed on 20 June 2020). Expression ratios for each categorized gene are shown in Figure 3, along with the overall modulation of each system/subsystem, which is represented by the sum of relative expression ratios of their individual genes (detailed information regarding individual genes present in each system/subsystem can be found in the Supplementary Materials File S3).

These analyses confirmed that many *Z. mobilis* structural/functional systems/subsystems display similar modulation patterns in all three transcriptome comparisons, suggesting that they are likely associated with entry into stationary phase, rather than AI-2-specific responses. For example, genes involved in processes associated with protein synthesis and metabolism are always negatively modulated, regardless of the presence of AI-2 (Figure 3A and Supplementary Materials File S3A). A similar situation can be verified with genes involved in general biosynthetic processes, with emphasis on amino acid and nucleotide biosynthesis (Figure 3B and Supplementary Materials File S3B), as well as in catabolic processes involved in the degradation of such biomolecules (Figure 3C and Supplementary Materials File S3C). However, when the transcriptome profiles of AI-2 treated cells (at t = 16 h + AI-2 and/or at t = 20 h + AI-2) are compared with untreated controls (at t = 20 h), significant differences can be found in the expression profile of genes involved in: (i) cell membrane composition (Figure 3D and Supplementary Materials File S3D); (ii) locomotion (Figure 3E and Supplementary Materials File S3E); (iii) DNA repair (Figure 3F and Supplementary Materials File S3F); (iv) Entner–Doudoroff glycolytic pathway (Figure 3G and Supplementary Materials File S3G) and (v) inorganic nutrient metabolism, with emphasis on nitrogen (N_2_) fixation (Figure 3H and Supplementary Materials File S3H), a metabolic process that has been proposed to bear significant biotechnological potential for the bioethanol industry [8,9].

### 2.4. The Presence of AI-2 Increases N_2_ Fixation by Z. mobilis

**Figure 4:**
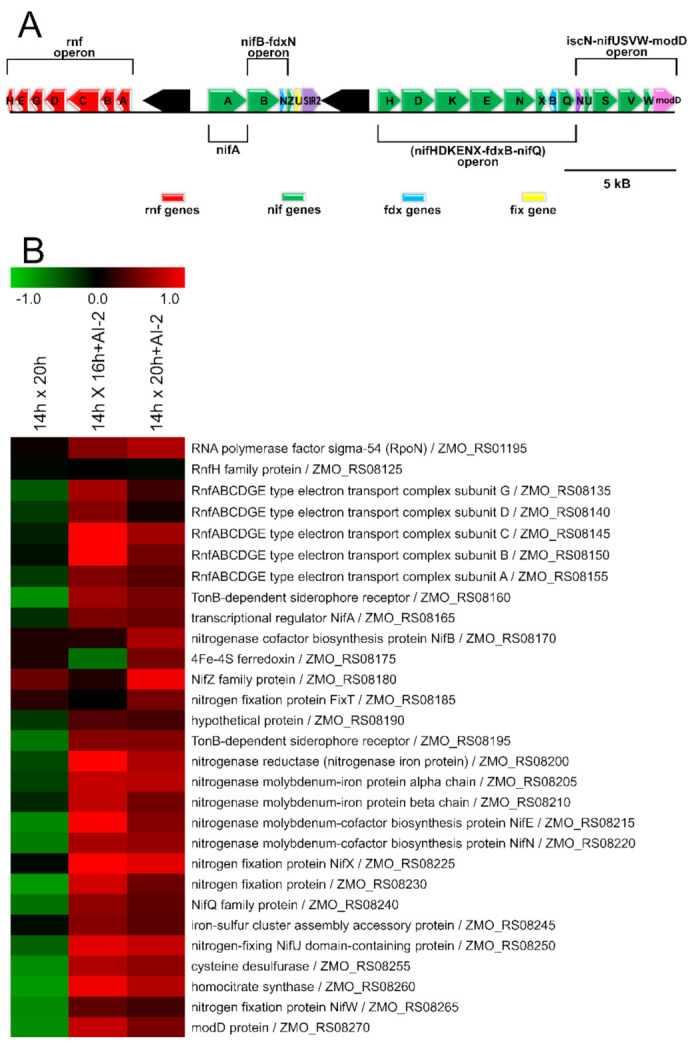
Transcriptional profile of genes responsible for N_2_ fixation in *Z. mobilis*, in response to AI-2. The upper panel (**A**) shows the N_2_ fixation locus present in the *Z. mobilis* chromosome. This locus consists of four operons, which contain genes directly involved in the production/maturation of nitrogenase, as well as those encoding electron donors, involved in the N_2_ fixation process. The bottom panel (**B**) shows the expression pattern of the genes contained in this locus, in the presence or absence of AI-2, according to our RNA-seq data. The top element in panel B shows the expression profile of gene ZMO_RS01195, which is located outside the N_2_ fixation locus shown in panel A. This element encodes an RpoN-like sigma factor (Sigma 54), supposedly involved in transcription of N_2_-fixation genes, which is also upregulated in response to AI-2.

The *Z. mobilis* genome contains all genes necessary to perform N_2_-fixation, including an RpoN-like sigma factor (Sigma 54) [13]. All additional genes involved in this process are located in a single chromosomal region [13], which includes the gene encoding the NifA regulator, the operon *nif*HDKENX-*fdx*B-*nif*Q (which encodes the nitrogenase enzyme), two operons involved in nitrogenase maturation (*ni*fB-*fdx*N and *isc*N-*nif*USVW-*mod*D), and the RNF operon (*rnf*ABCDGEH), which encodes members of the RNF electron-transport complex, responsible for donating electrons to nitrogenase, during N_2_ fixation. As observed in Figure 4, most of these elements were identified as positively modulated, in response to the presence of AI-2.

**Figure 5:**
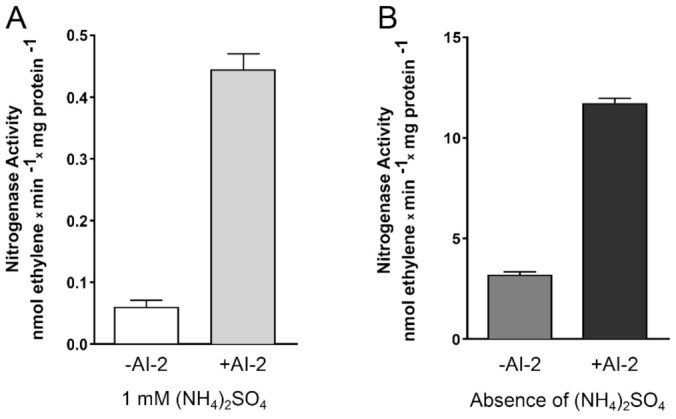
Acetylene reduction assay (ARA) to measure nitrogenase activity in *Z. mobilis*, in response to the presence of AI-2. *Z. mobilis* cells were inoculated in semi-solid MM, either in the absence or in the presence of 45 µM AI-2. After incubation for 48 h at 30 °C, 1 mL of 10% acetylene was injected into the headspace of the flasks, which were incubated for another hour, at 30 °C. The ethylene (C_2_H_4_) formed inside the bottles (resulting from the action of bacterial nitrogenase) was then detected and quantified by gas chromatography. The cellular biomass present in each flask was estimated by total protein quantification and the nitrogenase activity expressed as nmols of C_2_H_4_ produced/min/mg of protein. The experiments were carried out in triplicate and the graphs show the mean and SEMs obtained for each condition. The figure also compares the results obtained in an assay conducted in MM medium containing 1 mM (NH_4_)_2_SO_4_ (**A**) and a similar assay, conducted in MM lacking (NH_4_)_2_SO_4_ (**B**), since the presence of NH_4_^+^ is known to inhibit nitrogenase activity [16].

To verify if the AI-2-induced modulation verified in the abovementioned genes really affects nitrogen fixation in *Z. mobilis*, cultures of this bacterium were subjected to an acetylene reduction assay (ARA), the conventional methodology used to measure nitrogenase activity in bacterial cells [14]. In this assay, bacterial nitrogenase activity reduces acetylene (C_2_H_2_) to ethylene (C_2_H_4_), which is then measured by gas chromatography (see Materials and Methods for details). As observed in Figure 5, *Z. mobilis* cells incubated in the presence of 45 µM AI-2 showed a significant increase (~4–8×) in nitrogenase activity, when compared to cells incubated in the absence of this auto-inducer. Interestingly, N_2_ fixation appears to be stimulated by AI-2 both in the presence, or absence, of fixed nitrogen (NH_4_^+^) in the culture medium, although total enzyme activity is reduced in the first condition, as previously observed in other diazotrophic bacteria [15]. 

### 2.5. The Presence of AI-2 Favors Glucose-to-Ethanol Conversion, in Detriment of Biomass Accumulation in Z. mobilis Cells

As mentioned above, previous studies have suggested that activation of the N_2_-fixing mechanism in *Z. mobilis* could be economically relevant for bioethanol plants, which could use N_2_ gas as a substitute for industrial fertilizers, during the production of second-generation ethanol [8,9]. Interestingly, the activation of N_2_ fixation in *Z. mobilis* seems to favor glucose-to-ethanol conversion (instead of diverting this carbon source to biomass accumulation), resulting in more efficient ethanol/glucose ratios during fermentation [8]. Glucose internalization is mediated by a specific permease, encoded by gene ZMO_RS01265 [17]. Once inside the cell, this monosaccharide is degraded by the Entner–Doudoroff glycolytic pathway (ED), followed by pyruvate decarboxylation and acetaldehyde reduction to ethanol [18]. However, the presence of AI-2 seems to affect the expression of few genes involved in glucose-to-ethanol interconversion and it is difficult to infer the overall effect of AI-2 on ethanol production based solely on the RNA-seq data (see Supplementary Materials File S4).

**Figure 6:**
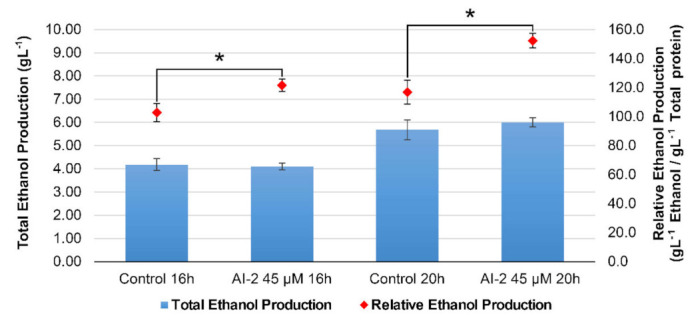
Ethanol production by *Z. mobilis* in response to AI-2. Cultures of *Z. mobilis* were established, as described in Figure 1 and grown for 14 h, until they reached mid exponential growth phase. At this time, AI-2 was added, at 45 µM final concentration, and the cultures were further incubated for additional six hours, until they all reached stationary growth phase. Aliquots (2 mL) were collected at times t = 16 h and t = 20 h for ethanol quantification, using the EnzyChrom^®^ Ethanol Assay kit, and to quantify total protein accumulated in the bacterial biomass, at these different timepoints (see Materials and Methods for details). Blue bars display the absolute ethanol concentration in each sample (left axis), while the red dots display their relative ethanol production, normalized by biomass accumulation (right axis). The graph shows the average results and their respective SEMs, obtained from three independent experiments. * represents statistically significant differences in relation to the respective controls (AI-2 untreated cultures), after a two-tailed *t*-test, using *p* < 0.05 as a threshold.

Thus, the production of ethanol by cells grown in the presence or absence of AI-2 was tested directly, using aliquots harvested from cultures grown under conditions similar to those shown in Figure 1. These aliquots (harvested at times t = 16 h and 20 h) were centrifuged and the concentration of ethanol in their supernatants was measured with the aid of the EnzyChrom^®^ Ethanol Assay kit (see Materials and Methods for details). The results showed that total ethanol production was not significantly affected in all cultures, regardless of the presence or absence of AI-2 (Figure 6). However, since AI-2 inhibits *Z. mobilis* growth (thus reducing cellular biomass), the ethanol-to-glucose turnover ratio, per cell, is significantly higher in AI-2-treated cultures, when compared to untreated controls (Figure 6). Overall, these results point to the possible use of this autoinducer in improved mechanisms for bioethanol production, based on boosting N_2_ fixation (see below).

## 3. Discussion

QS is defined as a chemical communication mechanism employed by bacteria to assess the population density in their surrounding environment and synchronize behavior on a community scale [1,2]. In this sense, QS seems to blur a well-established distinction between prokaryotes and eukaryotes, by allowing bacteria to coordinate their gene expression patterns not as single-cell individuals, but rather as observed in multicellular eukaryotic structures, such as tissues and organs [19]. Although different signaling molecules can be used to trigger this process, the QS response invariably involves behaviors that can be considered unproductive when performed by a few cells acting alone, but become beneficial to the community, when performed simultaneously by a large number of individuals, such as biofilm formation, motility, and toxin production, among others [3]. Moreover, the existence of a trans-specific QS signaling system, involving AI-2, allows the QS response to affect the behavior and composition of even complex bacterial communities, as recently demonstrated by studies involving the intestinal microbiota [20,21].

The studies presented herein show that *Z. mobilis* cells, while incapable of producing AI-2, can respond to the presence of this signaling molecule through a significant reduction in their growth rate, compatible with early induction of stationary phase. This effect is readily observed (<1 h after adding the signal to the cultures) and does not appear to derive from cytotoxic effects, as it does not affect cell viability and occurs (in a dose-dependent manner) under AI-2 concentrations typically involved with QS responses in *E. coli* and other bacteria (~20–50 µM) [22,23,24,25]. Accordingly, when treated with exogenous AI-2, *Z. mobilis* displays transcriptome alterations observed in cells naturally entering stationary phase, characterized by sub-regulation of genes involved in several energy-demanding processes, including protein synthesis, and other biosynthetic pathways, among others. However, specific AI-2-related responses can be found in the expression of genes involved in the composition of cellular membranes and elements associated with various processes, such as bacterial motility, DNA repair, inorganic nutrient metabolism, and carbohydrate degradation. Interestingly, similar scenarios have also been observed during the QS response in additional bacterial species [26,27,28,29,30,31,32,33,34,35,36,37,38,39,40,41,42,43,44,45].

For example, previous studies have already established the connection between the QS response and motility control in bacteria [41,46,47]. In most cases (as in *Serratia marcescens*, *Vibrio cholerae* and *E. coli*), the QS response leads to increased expression of genes related to flagellar structure, with consequent stimulation of bacterial motility [48,49,50]. This phenomenon is usually interpreted as an adaptation to population growth, with a consequent increase in competition for nutrients in the surrounding environment. Thus, increased motility causes population dispersion, allowing bacteria to colonize less populated niches, where reduced competition (or increased nutrient availability) may provide a more favorable environment [51]. In other cases, flagellar gene expression enables a specific form of motility known as swarming, often verified during the initial stages of biofilm formation [52,53,54].

However, there are cases in which the QS response can lead to decreased expression of flagellar genes, allowing bacteria to settle and accumulate at niches that favor their development and differentiation, as in the case of *Sinorhizobium meliloti*, which reduces flagella production at higher cellular densities. This process is mediated by a QS-1-type system (based on AHLs) known as ExpR/Sin, which controls bacterial adaptation to the symbiotic lifestyle in their host plants, during the development of nitrogen-fixing leguminous root nodules [51]. Interestingly, despite having at least two QS-1-type systems [55], *S. meliloti* is also able to respond to exogenous AI-2, although a more comprehensive assessment of which genes are affected by the presence of this signaling molecule is yet to be carried out [56]. 

Like *S. meliloti*, *Z. mobilis* also responds to the presence of AI-2, although both bacteria are unable to synthesize this molecule (a feature shared by all Alphaproteobacteria) [57]. Additionally, they are both diazotrophic bacteria, being able to fix nitrogen from gaseous N_2_. As mentioned earlier, one of the most notable aspects of *Z. mobilis* response to the presence of AI-2 involves increased expression of nitrogen-fixing genes. More importantly, although the original transcriptome data only allowed us to infer the positive effect of AI-2 on N_2_ fixation, we have been able to confirm that this autoinducer indeed stimulates this important metabolic process in *Z. mobilis*. These findings represent the first evidence to establish a direct correlation between a QS-2-type response (based AI-2) and the activation of N_2_ fixation. So far, only QS-1-type responses have been associated with the activation of the N_2_ fixation mechanism, especially by stimulating the development of root nodules in some diazotrophic bacteria [58]. For example, a set of C_6_-, C_7_- and C_8_-AHLs, produced by the enzyme RhiI stimulate the development of root nodules in *Rhizobium leguminosarum* [59], while *cin*I^−^ mutants decrease N_2_ fixation in root nodules of *Rhizobium etli* [60]. Both RhiI and SinI are AHL synthases involved in QS-1-type systems found in these bacteria [58].

The discovery that *Z. mobilis* responds to the presence of AI-2 with increased N_2_- fixing activity bears relevant biotechnological potential, since Kremer et al. (2015) [8] demonstrated that N_2_ gas can be used as a cheaper substitute for nitrogen sources traditionally used in the industrial production of second-generation ethanol. In fact, studies conducted by these authors showed that a *Z. mobilis*-based, second-generation bioethanol plant could save more than USD 1.5 million per year by replacing nitrogen supplements, such as corn steep liquor (CSL) or diammonium phosphate (DAP), for gaseous N_2_, directly harvested from the atmosphere [8]. Moreover, these studies also showed that activation of N_2_ fixation induces a curious change in the metabolic behavior of *Z. mobilis* cultures, as a metabolic flows analysis demonstrated that N_2_-fixing cells preferentially allocate energy resources to maintaining N_2_ fixation, in detriment of general biosynthetic processes [8]. Surprisingly, however, while this metabolic shift results in reduced bacterial growth rates and biomass accumulation, final ethanol yields are not affected during the fermentation process. In fact, fermentations conducted under N_2_-fixing conditions resulted in significant increase in the proportion of glucose molecules converted to ethanol (97%), as well as in the number of ethanol molecules produced per cell [8]. 

These results are in perfect agreement with the observations described herein. Apparently, activation of genes responsible for N_2_ fixation, triggered in response to AI-2, cause cells to redirect the use of ATP to fuel this highly energy-demanding mechanism (which consumes 8 ATPs for processing each N_2_ molecule), in detriment of other energy-consuming processes, such as biosynthesis and motility (whose genes are mostly downregulated, in response to this autoinducer). In such an energy-demanding scenario, it is not surprising to see that *Z. mobilis* adapts its metabolism to increase glucose turnover to ethanol, as this process is the main mechanism employed by this bacterium to produce ATP [8]. As mentioned before, ethanol production in *Z. mobilis* involves glucose degradation by the ED pathway, followed by pyruvate decarboxylation and acetaldehyde reduction. Interestingly, the RNA-seq data suggest that AI-2 does not appear to affect the transcription of most genes involved in this process, when cells incubated with AI-2 are compared to untreated controls (see Supplementary Materials S3G and S4). However, these modulations do not seem to significantly impact total ethanol production, as shown in Figure 6. Thus, the increment in relative ethanol yield per cell, verified in AI-2 treated cultures, is likely to derive from increased glucose uptake, as our transcriptome data show that AI-2-treated cells display increased expression of ZMO_RS01265, a Major Facilitator Superfamily (MFS) permease, located in the bacterial inner membrane, which has been identified as the main glucose transporter in *Z. mobilis* [17] (see Supplementary Materials File S1). In accord with this scenario, two carbohydrate-specific porins, located in the cellular outer membrane (ZMO_RS00270 and ZMO_RS08390), are also upregulated in response to AI-2 (see Supplementary Materials File S1).

From an environmental perspective, it is tempting to speculate if the *Z. mobilis* AI-2 response might have evolved as a strategy to outcompete invading microorganisms from different species. Since *Z. mobilis* does not produce AI-2, the capacity to detect higher concentrations of this autoinducer allows *Z. mobilis* to perceive increased cellular density of AI-2-producing microorganisms in the surrounding environment. In such a scenario, *Z. mobilis* cells have evolved to trigger a series of coordinated responses to increase their metabolic fitness and eliminate such invaders. To accomplish that, *Z. mobilis* activates N_2_ fixation (which allows cell survival even if nitrogenous nutrients become scarce, due to interspecies competition), and becomes more effective in glucose uptake, by overexpressing membrane transporters. Additionally, the increased glucose-to-ethanol turnover ratios lead to ethanol accumulation in the local environment, triggering toxic effects in most microorganisms, but preserving *Z. mobilis* cells, given their elevated tolerance to this alcohol [7].

In conclusion, activation of the AI-2-dependent QS response of *Z. mobilis* results in a series of metabolic alterations that directly affect important biological traits in this bacterium, influencing mechanisms such as glucose uptake, nitrogen fixation, relative ethanol yield and biomass accumulation—all of which bear significant biotechnological potential. Altogether, these findings may set the basis for the use of AI-2-based strategies to boost N_2_ fixation in *Z. mobilis*, which could lead to the development of improved biotransformation processes involving this bacterium (including the production of second-generation bioethanol). This could be achieved, for example, by employing mixed *Z. mobilis* cultures, containing AI-2-producing bacteria, or with the use of a genetically modified *Z. mobilis* strain carrying only two transgenes incorporated into the genome (*pfs* and *lux*S), which would render these cells capable of producing their own AI-2 [5]. 

## 4. Materials and Methods

### 4.1. Bacterial Growth and Extraction of Genetic Material

The ZM4 strain of *Z. mobilis* (ATCC 14990) was used throughout the entire project. Bacteria were grown in a *Z. mobilis*-specific minimal medium (MM), described by Goodman et al. (1982) [61]. The bacteria were routinely maintained in Petri dishes, containing solid MM medium (1.4% agar–agar Difco) (Sigma-Aldrich/Merck, Munich, Germany), at 30 °C. Fresh colonies were picked and transferred to fresh plates, every 10–15 days. Liquid cultures were established in Schott^®^ flasks (Schott Pharmaceuticals, Itupeva, Brazil), containing liquid MM medium, which were inoculated with isolated colonies, selected from the Petri dishes. In such cases, bacterial growth was carried out under semi-anaerobic conditions, without agitation, at 30 °C. Extraction of RNA from bacteria grown in liquid medium was done with the aid of the RNeasy^®^ kit (Qiagen, Hilden, Germany), according to the manufacturer’s recommendations.

### 4.2. Testing Z. mobilis Viability by Flow Cytometry Analyses

The LIVE/DEAD^®^ BacLigh^®^ Bacterial Viability Kit (Thermo Fisher Scientific, Waltham, MA, USA) was used according to the manufacturer´s instructions, to discern viable cells from damaged/dead cells, by flow cytometry. A positive control for damaged/dead cells was established by pre-treating cells with 70% isopropyl alcohol, as recommended in the kit. For cell staining, 10 µL of bacterial suspension were added into a flow cytometry analysis tube containing 0.85% NaCl solution, followed by the addition of 1.5 µL of 3.34 mM SYTO 9 and 1.5 µL of 30 mM propidium iodide (1 mL final volume). After incubation for 15 min at room temperature, protected from light, fluorescence emission was evaluated in a FACSCalibur flow cytometer (BD Biosciences, Franklin Lake, NJ, USA), acquiring 10,000 events per sample, using a blue laser with excitation at 488 nm, 502 LP dichroic mirror, 530/30 nm bandpass filter for SYTO 9 detection (FL1 channel), and 670 nm long-pass filter for PI detection (FL3 channel). Data analysis and graphs were made using FlowJo vX.0.7 software (BD Biosciences).

### 4.3. Transcriptomic Analysis of Z. mobilis in Response to the Presence of AI-2

The identification of AI-2-responsive genes in *Z. mobilis* was carried out by comparative transcriptome analyses, using RNA-seq [62]. For this purpose, bacterial cultures were grown in liquid MM for 16–18 h and used to inoculate 80 mL MM cultures, at an initial OD_600_ = 0.05. These cultures were incubated, at 30 °C (without shaking) and monitored by OD_600_ readings, until stationary growth phase was reached. Whenever appropriate, the cultures were stimulated with different concentrations of AI-2 (acquired from Carbosynth Ltd., San Diego, CA, USA), after reaching middle exponential growth phase (OD_600_ typically between 0.6 and 0.8), and further incubated, under the same conditions, for additional time periods. RNA samples, used for the construction of RNA-seq libraries, were obtained from cultures grown and treated under different conditions (middle of exponential phase and stationary phase, both in the presence and absence of AI-2). RNA-seq libraries were constructed with the aid of the ScriptSeq RNA Sequence Library Preparation Kit V2, according to manufacturer’s specifications (Illumina, Inc., Santa Clara, CA, USA). Libraries were produced with RNA samples extracted from three replicated cultures and sequenced in an Illumina Next-seq DNA sequencer, (2 × 75 cycles), according to the manufacturer’s specifications.

### 4.4. Bioinformatics Analyses

The sequences obtained from the various RNA-seq libraries were initially processed by Rockhopper [63], using the reference genome of *Z. mobilis* ZM4 [64]. Average gene expression values were calculated directly by the software and expressed by their average RPKM values. Differentially expressed genes were initially identified by an analysis of variance (ANOVA), followed by a Benjamini–Hochberg (BH) post-test, using q ≤ 0.01 as a cutoff for statistical significance. After statistical validation, the remaining data were manually curated to retain only genes displaying average absolute modulation values > 50% (−0.6 ≤ log_2_ (Experiment/Control) ≤ 0.6), when compared with controls (t = 14 h). The differentially expressed genes were initially visualized and subjected to hierarchical clustering with TMEV [11]. Analysis of the main cellular functions affected during the cell growth experiments was performed with the OMICS Dashboard tool, part of the Pathway Tools package (https://arxiv.org/abs/1510.03964, accessed on 20 June 2020), using the Pathway Genome Database (PGDB) of *Zymomonas mobilis* ZM4, available at the BioCyc repository (https://biocyc.org/, accessed on 20 June 2020).

### 4.5. Quantitative Real-Time PCR Experiments (qPCR)

Several genes identified as differentially expressed in the RNA-seq analysis were randomly selected for validation by qPCR. cDNA was prepared for each studied condition from 1 µg total RNA, previously incubated with 1 U of RQ1 DNase for 30 min, at 37 °C (10 µL final volume). Next, 1 µL of RQ1 Stop Solution was added to the reaction, which was then heated at 65 °C for 30 min, followed by the addition of 1 µL random nonamers (50 ng/µL) and 1 µL of a 10 mM dNTP solution. Primers were then allowed to anneal for 5 min, at 65 °C, and the cDNA synthesis was started by adding 4 µL of 5X First Strand Buffer, 2 µL 0.1 M DTT, 1 µL RNase Out (400 U/µL), and 1 µL Superscript II RT (200 U/µL). Incubation was conducted for 2 h, at 42 °C, followed by 15 min at 70 °C. Finally, the reactions were treated with 1 µL RNAse H (20 U/µL) for 20 min, at 37 °C (all enzymes and reagents were obtained from Invitrogen/Thermo Fisher Scientific, Carlsbad, CA, USA, except for the RQ1 DNase, which was obtained from Promega Corp., Madison, WI, USA). Samples of these cDNAs (100 ng) were then used in 10 µL qPCR reactions, containing EvaGreen PCR Master Mix (Solis Biodyne, Tartu, Estonia) and specific forward and reverse primers (0.25 nM each). All qPCR reactions were conducted in triplicate, in an ABI PRISM^®^ 7500 Real Time PCR System (Applied Biosystems, Waltham, MA, USA). Thermocycling conditions comprised an initial step at 50 °C for 2 min, followed by 15 min at 95 °C, and 40 cycles of (i) 95 °C for 20 s; (ii) 60 °C for 30 s, and (iii) 72 °C for 30 s. Specific primer sequences were obtained with the aid of PrimerQuest (https://www.idtdna.com/pages/tools/primerquest, accessed multiple times from January 2020 to December 2020) and are available at Supplementary Materials File S5. Gene ZMO_RS03720 (which encodes the enzyme UDP-N-acetylmuramate-L-alanine ligase) was used as an internal control to normalize expression ratios by the 2^−ΔΔCt^ method [65], since it did not display detectable transcriptional modulation throughout all conditions evaluated in the RNA-seq assays.

### 4.6. Ethanol Quantification

Ethanol quantification was carried with the aid of the EnzyChrom^®^ Ethanol Assay kit, according to the manufacturer’s specifications (BioAssay Systems, Hayward, CA, USA). Briefly, 1-mL aliquots of *Z. mobilis* cultures (grown in MM medium) were collected at different timepoints, centrifuged at 8000× *g* for 1 min and the resulting supernatant was filtered through 0.22 µm Microcon^®^ filters (Millipore, Burlington, VT, USA). Cells present in the centrifugation pellet were eluted in 1 × saline solution and used to quantify total protein present in the bacterial biomass with the aid of Bradford reagent, after treatment with 0.2 mM NaOH, for 30 min, according to the manufacturer’s specifications (Sigma-Aldrich/Merck). The filtered supernatant was submitted to serial dilution and 10 µL of each dilution was distributed into individual wells of a 96-well microplate, along with 90 µL of freshly prepared EnzyChrom^®^ reaction mixture (provided in the kit). The contents were mixed and incubated at room temperature for 30 min and reactions were interrupted by the addition of 100 µL of Stop Solution (provided in the kit). OD_595_ readings were performed in a Multiskan Go microplate reader (Thermo Fisher Scientific) and ethanol content was determined by interpolating the results with a standard curve, made with samples containing pre-defined ethanol concentrations.

### 4.7. Acetylene Reduction Assay (ARA)

Nitrogenase activity in *Z. mobilis* cells was estimated by the Acetylene Reduction Assay (ARA) [14]. Briefly, a *Z. mobilis* colony was inoculated in 5 mL MM and grown for 48 h, as described above. Subsequently, 40 µL of this culture were added to 4 mL of semi-solid MM (containing 0.175% agar), in triplicate, in either the absence or presence of 45 µM AI-2 (assays were also conducted with modified MM lacking (NH_4_)_2_SO_4_). The vials were then closed, using a rubber stopper and incubated for 48 h, at 30 °C. Next, 1 mL of 10% acetylene was injected into the headspace of the vials, which were then incubated at 30 °C, for 1 h. After this, 0.5 mL of the gas phase from the vials were collected with a 1-mL syringe and injected in a Trace GC Ultra gas chromatographer (Thermo Fisher Scientific), equipped with a Porapak N column and a flame ionization detector (FID), to quantify the ethylene produced. Finally, the cell biomass present in each flask was estimated by total protein quantification, with the aid of Bradford reagent, after treatment with 0.2 mM NaOH, for 30 min, according to the manufacturer´s specifications (Sigma-Aldrich/Merck). Nitrogenase activity was then calculated and expressed as nmols of ethylene (C_2_H_2_) produced/min/mg of protein.

## Data Availability

Raw sequencing reads are available at the NCBI Sequence Read Archive (SRA), under accession number SUB9185339 (provisional). Additional data derived from this study (including all Supplementary Materials) are also available from the Open Science Framework (OSF) repository (https://osf.io/rs8pu/, accessed on 25 April 2021).

## Supplementary Materials

The following are available online, at https://www.mdpi.com/article/10.3390/ijms22115628/s1, and at the Open Science Framework (OSF) repository (https://osf.io/rs8pu/, accessed on 25 April 2021): File S1—figure showing the gene expression patterns of the 724 genes from *Z. mobilis* modulated under the experimental conditions described in the manuscript; File S2—table comparing the relative expression data obtained by RNA-seq and qPCR); File S3—figure showing the gene expression patterns of the 724 genes from *Z. mobilis* modulated under the experimental conditions described in the manuscript, distributed in different functional/structural categories/subcategories; File S4—figure showing expression modulation in genes involved in glucose-to-ethanol interconversion in *Z. mobilis*; File S5—table containing primer sequences for the qPCR experiments.
