# Supplementary material for: The Quorum Sensing Auto-Inducer 2 (AI-2) Stimulates Nitrogen Fixation and Favors Ethanol Production over Biomass Accumulation in Zymomonas mobilis
Source: Int J Mol Sci. 2021 May 26;22(11):5628. doi: 10.3390/ijms22115628 (PMC8198075; doi:10.3390/ijms22115628)
Supplement: Supplementary file 1 [file ijms-22-05628-s001.zip › Supplementary/Supplementary Material S1.pdf]

Supplementary Material S1 – Expression patterns of the 724 *Z. mobilis* genes identified as modulated in response to AI-2.

Alencar *et al*. (2021). The quorum sensing auto-inducer 2 (AI-2) stimulates nitrogen fixation and favors ethanol production over biomass accumulation in *Zymomonas mobilis*. *Int. J. Mol. Sci. Submitted.*

.

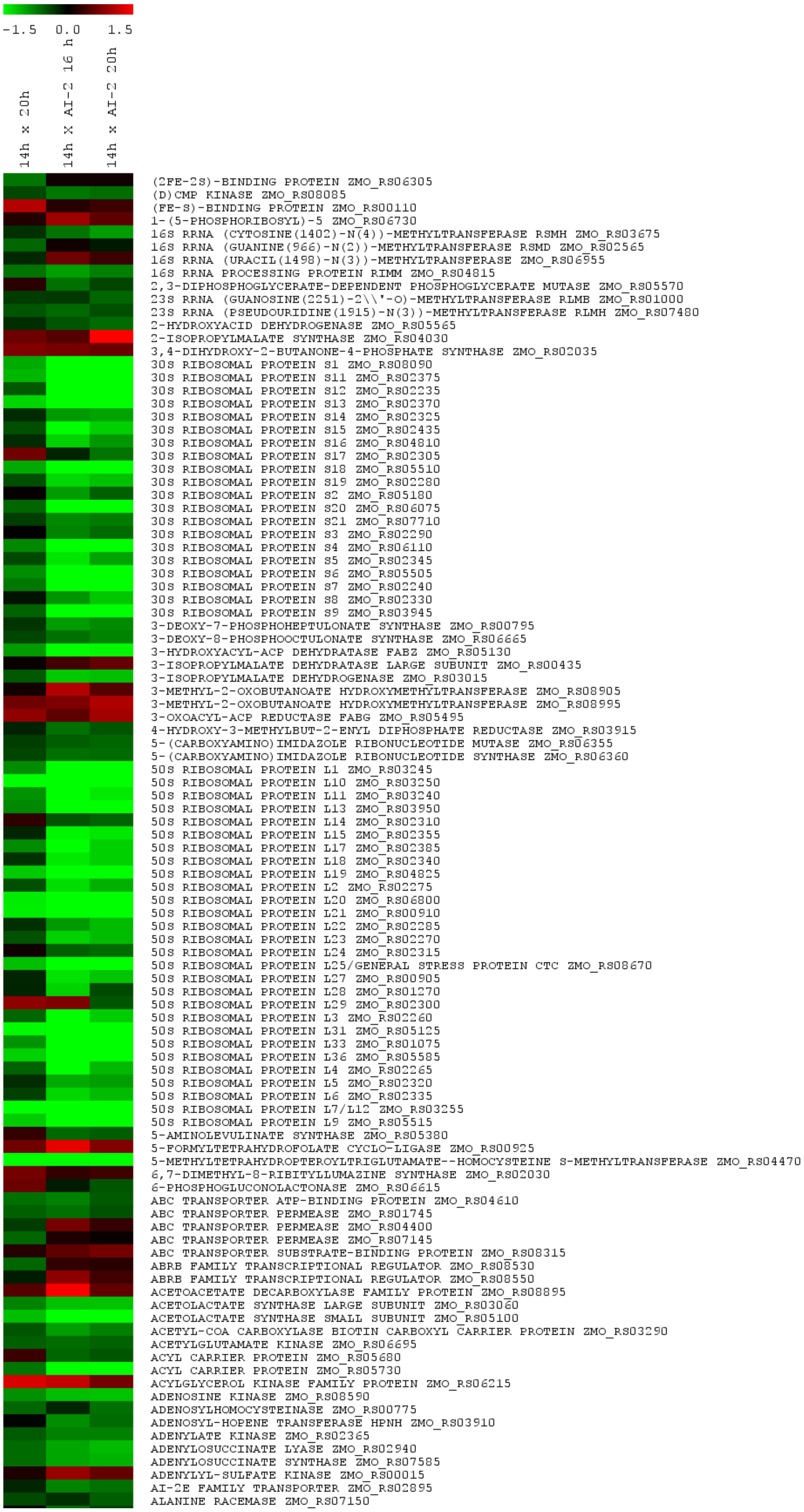
-1.5 0.0 1.5
14h x 20h
14h X AI-2 16 h
14h x AI-2 20h
(2FE-2S)-BINDING PROTEIN ZMO_RS06305
(D)CMP KINASE ZMO_RS08085
(FE-S)-BINDING PROTEIN ZMO_RS00110
1-(5-PHOSPHORIBOSYL)-5 ZMO_RS06730
16S RRNA (CYTOSINE(1402)-N(4))-METHYLTRANSFERASE RSMH ZMO_RS03675
16S RRNA (GUANINE(966)-N(2))-METHYLTRANSFERASE RSMD ZMO_RS02565
16S RRNA (URACIL(1498)-N(3))-METHYLTRANSFERASE ZMO_RS06955
16S RRNA PROCESSING PROTEIN RIMM ZMO_RS04815
2,3-DIPHOSPHOGLYCERATE-DEPENDENT PHOSPHOGLYCERATE MUTASE ZMO_RS05570
23S RRNA (GUANOSINE(2251)-2\\'-O)-METHYLTRANSFERASE RLMB ZMO_RS01000
23S RRNA (PSEUDOURIDINE(1915)-N(3))-METHYLTRANSFERASE RLMH ZMO_RS07480
2-HYDROXYACID DEHYDROGENASE ZMO_RS05565
2-ISOPROPYLMALATE SYNTHASE ZMO_RS04030
3,4-DIHYDROXY-2-BUTANONE-4-PHOSPHATE SYNTHASE ZMO_RS02035
30S RIBOSOMAL PROTEIN S1 ZMO_RS08090
30S RIBOSOMAL PROTEIN S11 ZMO_RS02375
30S RIBOSOMAL PROTEIN S12 ZMO_RS02235
30S RIBOSOMAL PROTEIN S13 ZMO_RS02370
30S RIBOSOMAL PROTEIN S14 ZMO_RS02325
30S RIBOSOMAL PROTEIN S15 ZMO_RS02435
30S RIBOSOMAL PROTEIN S16 ZMO_RS04810
30S RIBOSOMAL PROTEIN S17 ZMO_RS02305
30S RIBOSOMAL PROTEIN S18 ZMO_RS05510
30S RIBOSOMAL PROTEIN S19 ZMO_RS02280
30S RIBOSOMAL PROTEIN S2 ZMO_RS05180
30S RIBOSOMAL PROTEIN S20 ZMO_RS06075
30S RIBOSOMAL PROTEIN S21 ZMO_RS07710
30S RIBOSOMAL PROTEIN S3 ZMO_RS02290
30S RIBOSOMAL PROTEIN S4 ZMO_RS06110
30S RIBOSOMAL PROTEIN S5 ZMO_RS02345
30S RIBOSOMAL PROTEIN S6 ZMO_RS05505
30S RIBOSOMAL PROTEIN S7 ZMO_RS02240
30S RIBOSOMAL PROTEIN S8 ZMO_RS02330
30S RIBOSOMAL PROTEIN S9 ZMO_RS03945
3-DEOXY-7-PHOSPHOHEPTULONATE SYNTHASE ZMO_RS00795
3-DEOXY-8-PHOSPHOOCTULONATE SYNTHASE ZMO_RS06665
3-HYDROXYACYL-ACP DEHYDRATASE FABZ ZMO_RS05130
3-ISOPROPYLMALATE DEHYDRATASE LARGE SUBUNIT ZMO_RS00435
3-ISOPROPYLMALATE DEHYDROGENASE ZMO_RS03015
3-METHYL-2-OXOBUTANOATE HYDROXYMETHYLTRANSFERASE ZMO_RS08905
3-METHYL-2-OXOBUTANOATE HYDROXYMETHYLTRANSFERASE ZMO_RS08995
3-OXOACYL-ACP REDUCTASE FABG ZMO_RS05495
4-HYDROXY-3-METHYLBUT-2-ENYL DIPHOSPHATE REDUCTASE ZMO_RS03915
5-(CARBOXYAMINO)IMIDAZOLE RIBONUCLEOTIDE MUTASE ZMO_RS06355
5-(CARBOXYAMINO)IMIDAZOLE RIBONUCLEOTIDE SYNTHASE ZMO_RS06360
50S RIBOSOMAL PROTEIN L1 ZMO_RS03245
50S RIBOSOMAL PROTEIN L10 ZMO_RS03250
50S RIBOSOMAL PROTEIN L11 ZMO_RS03240
50S RIBOSOMAL PROTEIN L13 ZMO_RS03950
50S RIBOSOMAL PROTEIN L14 ZMO_RS02310
50S RIBOSOMAL PROTEIN L15 ZMO_RS02355
50S RIBOSOMAL PROTEIN L17 ZMO_RS02385
50S RIBOSOMAL PROTEIN L18 ZMO_RS02340
50S RIBOSOMAL PROTEIN L19 ZMO_RS04825
50S RIBOSOMAL PROTEIN L2 ZMO_RS02275
50S RIBOSOMAL PROTEIN L20 ZMO_RS06800
50S RIBOSOMAL PROTEIN L21 ZMO_RS00910
50S RIBOSOMAL PROTEIN L22 ZMO_RS02285
50S RIBOSOMAL PROTEIN L23 ZMO_RS02270
50S RIBOSOMAL PROTEIN L24 ZMO_RS02315
50S RIBOSOMAL PROTEIN L25/GENERAL STRESS PROTEIN CTC ZMO_RS08670
50S RIBOSOMAL PROTEIN L27 ZMO_RS00905
50S RIBOSOMAL PROTEIN L28 ZMO_RS01270
50S RIBOSOMAL PROTEIN L29 ZMO_RS02300
50S RIBOSOMAL PROTEIN L3 ZMO_RS02260
50S RIBOSOMAL PROTEIN L31 ZMO_RS05125
50S RIBOSOMAL PROTEIN L33 ZMO_RS01075
50S RIBOSOMAL PROTEIN L36 ZMO_RS05585
50S RIBOSOMAL PROTEIN L4 ZMO_RS02265
50S RIBOSOMAL PROTEIN L5 ZMO_RS02320
50S RIBOSOMAL PROTEIN L6 ZMO_RS02335
50S RIBOSOMAL PROTEIN L7/L12 ZMO_RS03255
50S RIBOSOMAL PROTEIN L9 ZMO_RS05515
5-AMINOLEVULINATE SYNTHASE ZMO_RS05380
5-FORMYLTETRAHYDROFOLATE CYCLO-LIGASE ZMO_RS00925
5-METHYLTETRAHYDROPTEROYLTRIGLUTAMATE--HOMOCYSTEINE S-METHYLTRANSFERASE ZMO_RS04470
6,7-DIMETHYL-8-RIBITYLLUMAZINE SYNTHASE ZMO_RS02030
6-PHOSPHOGLUCONOLACTONASE ZMO_RS06615
ABC TRANSPORTER ATP-BINDING PROTEIN ZMO_RS04610
ABC TRANSPORTER PERMEASE ZMO_RS01745
ABC TRANSPORTER PERMEASE ZMO_RS04400
ABC TRANSPORTER PERMEASE ZMO_RS07145
ABC TRANSPORTER SUBSTRATE-BINDING PROTEIN ZMO_RS08315
ABRB FAMILY TRANSCRIPTIONAL REGULATOR ZMO_RS08530
ABRB FAMILY TRANSCRIPTIONAL REGULATOR ZMO_RS08550
ACETOACETATE DECARBOXYLASE FAMILY PROTEIN ZMO_RS08895
ACETOLACTATE SYNTHASE LARGE SUBUNIT ZMO_RS03060
ACETOLACTATE SYNTHASE SMALL SUBUNIT ZMO_RS05100
ACETYL-COA CARBOXYLASE BIOTIN CARBOXYL CARRIER PROTEIN ZMO_RS03290
ACETYLGLUTAMATE KINASE ZMO_RS06695
ACYL CARRIER PROTEIN ZMO_RS05680
ACYL CARRIER PROTEIN ZMO_RS05730
ACYLGLYCEROL KINASE FAMILY PROTEIN ZMO_RS06215
ADENOSINE KINASE ZMO_RS08590
ADENOSYLHOMOCYSTEINASE ZMO_RS00775
ADENOSYL-HOPENE TRANSFERASE HPNH ZMO_RS03910
ADENYLATE KINASE ZMO_RS02365
ADENYLOSUCCINATE LYASE ZMO_RS02940
ADENYLOSUCCINATE SYNTHASE ZMO_RS07585
ADENYLYL-SULFATE KINASE ZMO_RS00015
AI-2E FAMILY TRANSPORTER ZMO_RS02895
ALANINE RACEMASE ZMO_RS07150

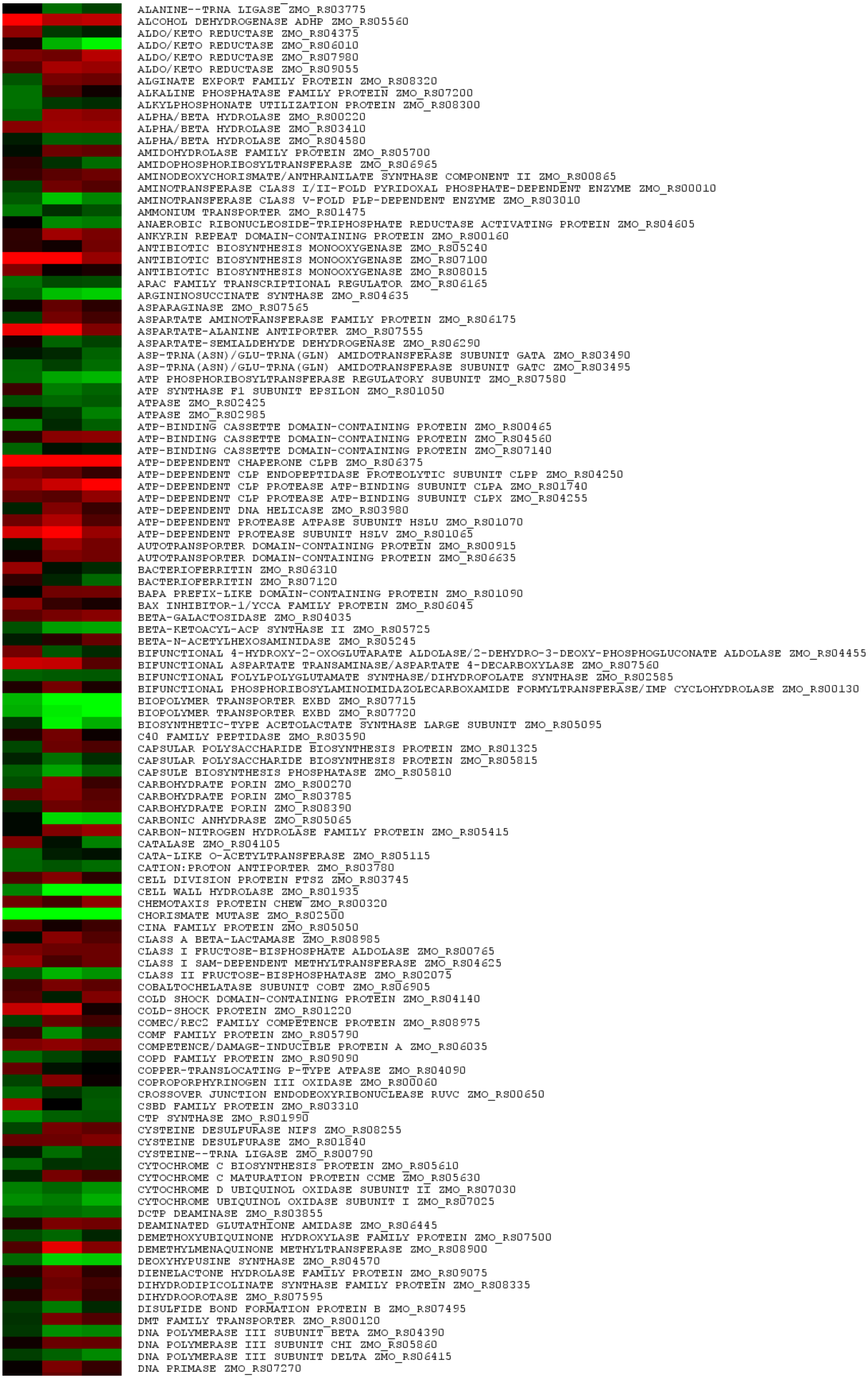
ALANINE--TRNA LIGASE ZMO_RS03775
ALCOHOL DEHYDROGENASE ADHP ZMO_RS05560
ALDO/KETO REDUCTASE ZMO_RS04375
ALDO/KETO REDUCTASE ZMO_RS06010
ALDO/KETO REDUCTASE ZMO_RS07980
ALDO/KETO REDUCTASE ZMO_RS09055
ALGINATE EXPORT FAMILY PROTEIN ZMO_RS08320
ALKALINE PHOSPHATASE FAMILY PROTEIN ZMO_RS07200
ALKYLPHOSPHONATE UTILIZATION PROTEIN ZMO_RS08300
ALPHA/BETA HYDROLASE ZMO_RS00220
ALPHA/BETA HYDROLASE ZMO_RS03410
ALPHA/BETA HYDROLASE ZMO_RS04580
AMIDOHYDROLASE FAMILY PROTEIN ZMO_RS05700
AMIDOPHOSPHORIBOSYLTRANSFERASE ZMO_RS06965
AMINODEOXYCHORISMATE/ANTHRANILATE SYNTHASE COMPONENT II ZMO_RS00865
AMINOTRANSFERASE CLASS I/II-FOLD PYRIDOXAL PHOSPHATE-DEPENDENT ENZYME ZMO_RS00010
AMINOTRANSFERASE CLASS V-FOLD PLP-DEPENDENT ENZYME ZMO_RS03010
AMMONIUM TRANSPORTER ZMO_RS01475
ANAEROBIC RIBONUCLEOSIDE-TRIPHOSPHATE REDUCTASE ACTIVATING PROTEIN ZMO_RS04605
ANKYRIN REPEAT DOMAIN-CONTAINING PROTEIN ZMO_RS00160
ANTIBIOTIC BIOSYNTHESIS MONOOXYGENASE ZMO_RS05240
ANTIBIOTIC BIOSYNTHESIS MONOOXYGENASE ZMO_RS07100
ANTIBIOTIC BIOSYNTHESIS MONOOXYGENASE ZMO_RS08015
ARAC FAMILY TRANSCRIPTIONAL REGULATOR ZMO_RS06165
ARGININOSUCCINATE SYNTHASE ZMO_RS04635
ASPARAGINASE ZMO_RS07565
ASPARTATE AMINOTRANSFERASE FAMILY PROTEIN ZMO_RS06175
ASPARTATE-ALANINE ANTIPORTER ZMO_RS07555
ASPARTATE-SEMIALDEHYDE DEHYDROGENASE ZMO_RS06290
ASP-TRNA(ASN)/GLU-TRNA(GLN) AMIDOTRANSFERASE SUBUNIT GATA ZMO_RS03490
ASP-TRNA(ASN)/GLU-TRNA(GLN) AMIDOTRANSFERASE SUBUNIT GATC ZMO_RS03495
ATP PHOSPHORIBOSYLTRANSFERASE REGULATORY SUBUNIT ZMO_RS07580
ATP SYNTHASE F1 SUBUNIT EPSILON ZMO_RS01050
ATPASE ZMO_RS02425
ATPASE ZMO_RS02985
ATP-BINDING CASSETTE DOMAIN-CONTAINING PROTEIN ZMO_RS00465
ATP-BINDING CASSETTE DOMAIN-CONTAINING PROTEIN ZMO_RS04560
ATP-BINDING CASSETTE DOMAIN-CONTAINING PROTEIN ZMO_RS07140
ATP-DEPENDENT CHAPERONE CLPB ZMO_RS06375
ATP-DEPENDENT CLP ENDOPEPTIDASE PROTEOLYTIC SUBUNIT CLPP ZMO_RS04250
ATP-DEPENDENT CLP PROTEASE ATP-BINDING SUBUNIT CLPA ZMO_RS01740
ATP-DEPENDENT CLP PROTEASE ATP-BINDING SUBUNIT CLPX ZMO_RS04255
ATP-DEPENDENT DNA HELICASE ZMO_RS03980
ATP-DEPENDENT PROTEASE ATPASE SUBUNIT HSLU ZMO_RS01070
ATP-DEPENDENT PROTEASE SUBUNIT HSLV ZMO_RS01065
AUTOTRANSPORTER DOMAIN-CONTAINING PROTEIN ZMO_RS00915
AUTOTRANSPORTER DOMAIN-CONTAINING PROTEIN ZMO_RS06635
BACTERIOFERRITIN ZMO_RS06310
BACTERIOFERRITIN ZMO_RS07120
BAPA PREFIX-LIKE DOMAIN-CONTAINING PROTEIN ZMO_RS01090
BAX INHIBITOR-1/YCCA FAMILY PROTEIN ZMO_RS06045
BETA-GALACTOSIDASE ZMO_RS04035
BETA-KETOACYL-ACP SYNTHASE II ZMO_RS05725
BETA-N-ACETYLHEXOSAMINIDASE ZMO_RS05245
BIFUNCTIONAL 4-HYDROXY-2-OXOGLUTARATE ALDOLASE/2-DEHYDRO-3-DEOXY-PHOSPHOGLUCONATE ALDOLASE ZMO_RS04455
BIFUNCTIONAL ASPARTATE TRANSAMINASE/ASPARTATE 4-DECARBOXYLASE ZMO_RS07560
BIFUNCTIONAL FOLYLPOLYGLUTAMATE SYNTHASE/DIHYDROFOLATE SYNTHASE ZMO_RS02585
BIFUNCTIONAL PHOSPHORIBOSYLAMINOIMIDAZOLECARBOXAMIDE FORMYLTRANSFERASE/IMP CYCLOHYDROLASE ZMO_RS00130
BIOPOLYMER TRANSPORTER EXBD ZMO_RS07715
BIOPOLYMER TRANSPORTER EXBD ZMO_RS07720
BIOSYNTHETIC-TYPE ACETOLACTATE SYNTHASE LARGE SUBUNIT ZMO_RS05095
C40 FAMILY PEPTIDASE ZMO_RS03590
CAPSULAR POLYSACCHARIDE BIOSYNTHESIS PROTEIN ZMO_RS01325
CAPSULAR POLYSACCHARIDE BIOSYNTHESIS PROTEIN ZMO_RS05815
CAPSULE BIOSYNTHESIS PHOSPHATASE ZMO_RS05810
CARBOHYDRATE PORIN ZMO_RS00270
CARBOHYDRATE PORIN ZMO_RS03785
CARBOHYDRATE PORIN ZMO_RS08390
CARBONIC ANHYDRASE ZMO_RS05065
CARBON-NITROGEN HYDROLASE FAMILY PROTEIN ZMO_RS05415
CATALASE ZMO_RS04105
CATA-LIKE O-ACETYLTRANSFERASE ZMO_RS05115
CATION:PROTON ANTIPORTER ZMO_RS03780
CELL DIVISION PROTEIN FTSZ ZMO_RS03745
CELL WALL HYDROLASE ZMO_RS01935
CHEMOTAXIS PROTEIN CHEW ZMO_RS00320
CHORISMATE MUTASE ZMO_RS02500
CINA FAMILY PROTEIN ZMO_RS05050
CLASS A BETA-LACTAMASE ZMO_RS08985
CLASS I FRUCTOSE-BISPHOSPHATE ALDOLASE ZMO_RS00765
CLASS I SAM-DEPENDENT METHYLTRANSFERASE ZMO_RS04625
CLASS II FRUCTOSE-BISPHOSPHATASE ZMO_RS02075
COBALTOCHELATASE SUBUNIT COBT ZMO_RS06905
COLD SHOCK DOMAIN-CONTAINING PROTEIN ZMO_RS04140
COLD-SHOCK PROTEIN ZMO_RS01220
COMEC/REC2 FAMILY COMPETENCE PROTEIN ZMO_RS08975
COMF FAMILY PROTEIN ZMO_RS05790
COMPETENCE/DAMAGE-INDUCIBLE PROTEIN A ZMO_RS06035
COPD FAMILY PROTEIN ZMO_RS09090
COPPER-TRANSLOCATING P-TYPE ATPASE ZMO_RS04090
COPROPORPHYRINOGEN III OXIDASE ZMO_RS00060
CROSSOVER JUNCTION ENDODEOXYRIBONUCLEASE RUVC ZMO_RS00650
CSBD FAMILY PROTEIN ZMO_RS03310
CTP SYNTHASE ZMO_RS01990
CYSTEINE DESULFURASE NIFS ZMO_RS08255
CYSTEINE DESULFURASE ZMO_RS01840
CYSTEINE--TRNA LIGASE ZMO_RS00790
CYTOCHROME C BIOSYNTHESIS PROTEIN ZMO_RS05610
CYTOCHROME C MATURATION PROTEIN CCME ZMO_RS05630
CYTOCHROME D UBIQUINOL OXIDASE SUBUNIT II ZMO_RS07030
CYTOCHROME UBIQUINOL OXIDASE SUBUNIT I ZMO_RS07025
DCTP DEAMINASE ZMO_RS03855
DEAMINATED GLUTATHIONE AMIDASE ZMO_RS06445
DEMETHOXYUBIQUINONE HYDROXYLASE FAMILY PROTEIN ZMO_RS07500
DEMETHYLMENAQUINONE METHYLTRANSFERASE ZMO_RS08900
DEOXYHYPUSINE SYNTHASE ZMO_RS04570
DIENELACTONE HYDROLASE FAMILY PROTEIN ZMO_RS09075
DIHYDRODIPICOLINATE SYNTHASE FAMILY PROTEIN ZMO_RS08335
DIHYDROOROTASE ZMO_RS07595
DISULFIDE BOND FORMATION PROTEIN B ZMO_RS07495
DMT FAMILY TRANSPORTER ZMO_RS00120
DNA POLYMERASE III SUBUNIT BETA ZMO_RS04390
DNA POLYMERASE III SUBUNIT CHI ZMO_RS05860
DNA POLYMERASE III SUBUNIT DELTA ZMO_RS06415
DNA PRIMASE ZMO_RS07270

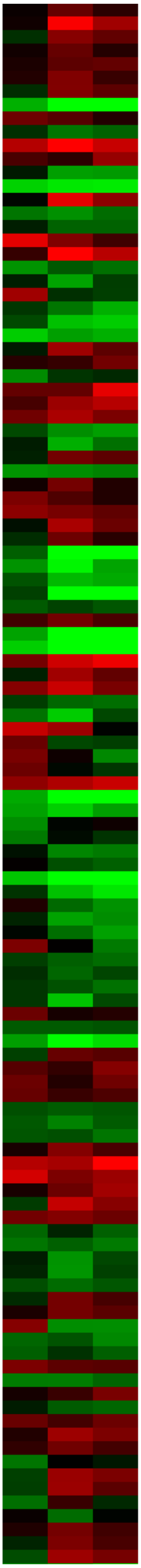
DNA RECOMBINATION PROTEIN RMUC ZMO_RS03630
DNA REPAIR PROTEIN RADC ZMO_RS08780
DNA REPLICATION/REPAIR PROTEIN RECF ZMO_RS07115
DNA TOPOISOMERASE (ATP-HYDROLYZING) SUBUNIT B ZMO_RS07110
DNA TOPOISOMERASE IV SUBUNIT B ZMO_RS01770
DNA TRANSLOCASE FTSK ZMO_RS07660
DNA-3-METHYLADENINE GLYCOSYLASE 2 FAMILY PROTEIN ZMO_RS01620
DNA-DIRECTED RNA POLYMERASE SUBUNIT ALPHA ZMO_RS02380
DNA-DIRECTED RNA POLYMERASE SUBUNIT BETA\\' ZMO_RS03270
DNA-DIRECTED RNA POLYMERASE SUBUNIT OMEGA ZMO_RS07745
DNAJ DOMAIN-CONTAINING PROTEIN ZMO_RS07600
DNTP TRIPHOSPHOHYDROLASE ZMO_RS06650
DO FAMILY SERINE ENDOPEPTIDASE ZMO_RS01015
DUF1013 DOMAIN-CONTAINING PROTEIN ZMO_RS03830
DUF1016 FAMILY PROTEIN ZMO_RS08770
DUF192 DOMAIN-CONTAINING PROTEIN ZMO_RS03960
DUF2093 DOMAIN-CONTAINING PROTEIN ZMO_RS06815
DUF2141 DOMAIN-CONTAINING PROTEIN ZMO_RS06220
DUF2285 DOMAIN-CONTAINING PROTEIN ZMO_RS08815
DUF2474 DOMAIN-CONTAINING PROTEIN ZMO_RS07035
DUF2497 DOMAIN-CONTAINING PROTEIN ZMO_RS08500
DUF308 DOMAIN-CONTAINING PROTEIN ZMO_RS06870
DUF3297 FAMILY PROTEIN ZMO_RS07830
DUF3576 DOMAIN-CONTAINING PROTEIN ZMO_RS06430
DUF3617 FAMILY PROTEIN ZMO_RS05605
DUF4136 DOMAIN-CONTAINING PROTEIN ZMO_RS05580
DUF4136 DOMAIN-CONTAINING PROTEIN ZMO_RS07365
DUF4167 DOMAIN-CONTAINING PROTEIN ZMO_RS06780
DUF4186 DOMAIN-CONTAINING PROTEIN ZMO_RS04520
DUF423 DOMAIN-CONTAINING PROTEIN ZMO_RS02635
DUF445 DOMAIN-CONTAINING PROTEIN ZMO_RS01255
DUF459 DOMAIN-CONTAINING PROTEIN ZMO_RS01915
DUF465 DOMAIN-CONTAINING PROTEIN ZMO_RS04430
DUF5309 DOMAIN-CONTAINING PROTEIN ZMO_RS01655
DUF721 DOMAIN-CONTAINING PROTEIN ZMO_RS07530
EFFLUX RND TRANSPORTER PERIPLASMIC ADAPTOR SUBUNIT ZMO_RS03470
ELECTRON TRANSFER FLAVOPROTEIN SUBUNIT ALPHA ZMO_RS06620
ELECTRON TRANSFER FLAVOPROTEIN SUBUNIT BETA/FIXA FAMILY PROTEIN ZMO_RS06625
ELECTRON TRANSPORT COMPLEX SUBUNIT RSXC ZMO_RS08145
ELECTRON TRANSPORT COMPLEX SUBUNIT RSXG ZMO_RS08135
ELONGATION FACTOR G ZMO_RS02245
ELONGATION FACTOR P ZMO_RS01410
ELONGATION FACTOR TS ZMO_RS05175
ELONGATION FACTOR TU ZMO_RS02250
ENDOLYTIC TRANSGLYCOSYLASE MLTG ZMO_RS05720
ENDOPEPTIDASE LA ZMO_RS01615
ENERGY TRANSDUCER TONB ZMO_RS07730
ENERGY-DEPENDENT TRANSLATIONAL THROTTLE PROTEIN ETTA ZMO_RS00780
EPOXYQUEUOSINE REDUCTASE QUEH ZMO_RS08540
ETHANOLAMINE PERMEASE ZMO_RS06160
EXCINUCLEASE ABC SUBUNIT UVRB ZMO_RS01550
EXODEOXYRIBONUCLEASE III ZMO_RS06260
EXODEOXYRIBONUCLEASE VII SMALL SUBUNIT ZMO_RS03825
F0F1 ATP SYNTHASE SUBUNIT ALPHA ZMO_RS01035
F0F1 ATP SYNTHASE SUBUNIT C ZMO_RS02975
F0F1 ATP SYNTHASE SUBUNIT DELTA ZMO_RS01030
F0F1 ATP SYNTHASE SUBUNIT GAMMA ZMO_RS01040
FAD:PROTEIN FMN TRANSFERASE ZMO_RS08290
FERREDOXIN FAMILY PROTEIN ZMO_RS00940
FERREDOXIN--NADP REDUCTASE ZMO_RS07885
FERROUS IRON TRANSPORT PROTEIN A ZMO_RS06890
FKBP-TYPE PEPTIDYL-PROLYL CIS-TRANS ISOMERASE ZMO_RS07705
FLAGELLAR BASAL BODY L-RING PROTEIN FLGH ZMO_RS02705
FLAGELLAR BASAL BODY P-RING PROTEIN FLGI ZMO_RS02700
FLAGELLAR BASAL BODY ROD PROTEIN FLGB ZMO_RS02735
FLAGELLAR BASAL BODY ROD PROTEIN FLGC ZMO_RS02730
FLAGELLAR BASAL-BODY ROD PROTEIN FLGF ZMO_RS02715
FLAGELLAR BASAL-BODY ROD PROTEIN FLGG ZMO_RS02710
FLAGELLAR HOOK CAPPING PROTEIN ZMO_RS02725
FLAGELLAR HOOK-BASAL BODY COMPLEX PROTEIN ZMO_RS02720
FLAGELLAR MOTOR SWITCH PROTEIN FLIG ZMO_RS02815
FLAGELLAR MOTOR SWITCH PROTEIN FLIN ZMO_RS02855
FLAGELLAR M-RING PROTEIN FLIF ZMO_RS02810
FLAGELLAR PROTEIN FLIS ZMO_RS02890
FLAGELLIN ZMO_RS02795
FORMATE--TETRAHYDROFOLATE LIGASE ZMO_RS01960
FYDLN ACID DOMAIN-CONTAINING PROTEIN ZMO_RS08075
GLSB/YEAQ/YMGE FAMILY STRESS RESPONSE MEMBRANE PROTEIN ZMO_RS05765
GLUCONOKINASE ZMO_RS07905
GLUCOSE-6-PHOSPHATE ISOMERASE ZMO_RS05445
GLUTAMATE 5-KINASE ZMO_RS00890
GLUTAMATE--TRNA LIGASE ZMO_RS04015
GLUTAMINE-HYDROLYZING CARBAMOYL-PHOSPHATE SYNTHASE SMALL SUBUNIT ZMO_RS07260
GLUTAMINE-HYDROLYZING GMP SYNTHASE ZMO_RS05670
GLUTAMINYL-PEPTIDE CYCLOTRANSFERASE ZMO_RS08505
GLUTAREDOXIN 2 ZMO_RS00290
GLUTAREDOXIN 3 ZMO_RS03370
GLUTATHIONE-DISULFIDE REDUCTASE ZMO_RS05440
GLYCERATE KINASE ZMO_RS00250
GLYCINE ZIPPER 2TM DOMAIN-CONTAINING PROTEIN ZMO_RS01875
GLYCINE--TRNA LIGASE SUBUNIT ALPHA ZMO_RS06475
GLYCINE--TRNA LIGASE SUBUNIT BETA ZMO_RS06470
GLYCOSYL TRANSFERASE FAMILY 2 ZMO_RS02790
GLYCOSYLTRANSFERASE FAMILY 2 PROTEIN ZMO_RS05820
GLYCOSYLTRANSFERASE ZMO_RS04245
GLYCOSYLTRANSFERASE ZMO_RS07815
GLYOXYLATE/HYDROXYPYRUVATE REDUCTASE A ZMO_RS08535
GNAT FAMILY N-ACETYLTRANSFERASE ZMO_RS00900
GNTP FAMILY PERMEASE ZMO_RS07900
GTPASE ERA ZMO_RS06180
GTPASE OBGE ZMO_RS00895
GUANYLATE KINASE ZMO_RS01870
HAD FAMILY PHOSPHATASE ZMO_RS08410
HAD-IB FAMILY HYDROLASE ZMO_RS02925
HEAT-INDUCIBLE TRANSCRIPTIONAL REPRESSOR HRCA ZMO_RS00075
HELICASE ZMO_RS00935
HELIX-TURN-HELIX DOMAIN-CONTAINING PROTEIN ZMO_RS00875
HELIX-TURN-HELIX TRANSCRIPTIONAL REGULATOR ZMO_RS03120
HEME ABC EXPORTER ATP-BINDING PROTEIN CCMA ZMO_RS01125
HEME LYASE CCMF/NRFE FAMILY SUBUNIT ZMO_RS05625
HEPARINASE II/III FAMILY PROTEIN ZMO_RS00095
HISTIDINE KINASE ZMO_RS03055
HLYD FAMILY EFFLUX TRANSPORTER PERIPLASMIC ADAPTOR SUBUNIT ZMO_RS01105
HOLLIDAY JUNCTION BRANCH MIGRATION PROTEIN RUVA ZMO_RS00655
HOMOCITRATE SYNTHASE ZMO_RS08260

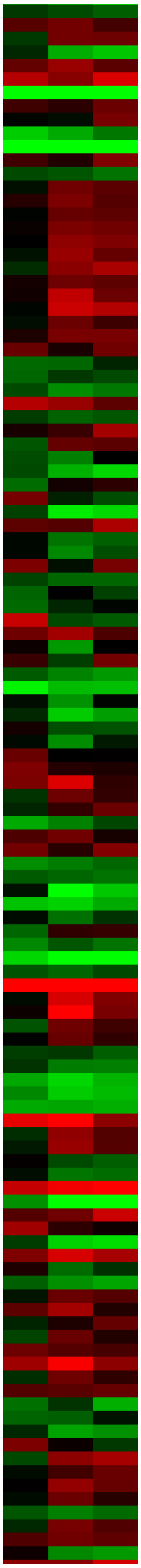

HOMOSERINE KINASE ZMO_RS07180
HOMOSERINE O-ACETYLTRANSFERASE ZMO_RS00965
HPNL FAMILY PROTEIN ZMO_RS04365
HPNM FAMILY PROTEIN ZMO_RS03920
HPR FAMILY PHOSPHOCARRIER PROTEIN ZMO_RS05950
HSP20 FAMILY PROTEIN ZMO_RS04435
HU FAMILY DNA-BINDING PROTEIN ZMO_RS01060
HYDROXYACYLGLUTATHIONE HYDROLASE ZMO_RS03395
HYPOTHETICAL PROTEIN ZMO_RS00055
HYPOTHETICAL PROTEIN ZMO_RS00660
HYPOTHETICAL PROTEIN ZMO_RS00785
HYPOTHETICAL PROTEIN ZMO_RS00995
HYPOTHETICAL PROTEIN ZMO_RS01285
HYPOTHETICAL PROTEIN ZMO_RS01635
HYPOTHETICAL PROTEIN ZMO_RS01640
HYPOTHETICAL PROTEIN ZMO_RS01645
HYPOTHETICAL PROTEIN ZMO_RS01660
HYPOTHETICAL PROTEIN ZMO_RS01665
HYPOTHETICAL PROTEIN ZMO_RS01670
HYPOTHETICAL PROTEIN ZMO_RS01675
HYPOTHETICAL PROTEIN ZMO_RS01690
HYPOTHETICAL PROTEIN ZMO_RS01695
HYPOTHETICAL PROTEIN ZMO_RS01700
HYPOTHETICAL PROTEIN ZMO_RS01705
HYPOTHETICAL PROTEIN ZMO_RS01715
HYPOTHETICAL PROTEIN ZMO_RS01895
HYPOTHETICAL PROTEIN ZMO_RS02050
HYPOTHETICAL PROTEIN ZMO_RS02070
HYPOTHETICAL PROTEIN ZMO_RS02785
HYPOTHETICAL PROTEIN ZMO_RS03000
HYPOTHETICAL PROTEIN ZMO_RS03335
HYPOTHETICAL PROTEIN ZMO_RS03430
HYPOTHETICAL PROTEIN ZMO_RS03535
HYPOTHETICAL PROTEIN ZMO_RS03635
HYPOTHETICAL PROTEIN ZMO_RS03680
HYPOTHETICAL PROTEIN ZMO_RS04165
HYPOTHETICAL PROTEIN ZMO_RS04505
HYPOTHETICAL PROTEIN ZMO_RS04955
HYPOTHETICAL PROTEIN ZMO_RS05405
HYPOTHETICAL PROTEIN ZMO_RS05460
HYPOTHETICAL PROTEIN ZMO_RS05785
HYPOTHETICAL PROTEIN ZMO_RS05825
HYPOTHETICAL PROTEIN ZMO_RS05890
HYPOTHETICAL PROTEIN ZMO_RS05900
HYPOTHETICAL PROTEIN ZMO_RS05910
HYPOTHETICAL PROTEIN ZMO_RS06135
HYPOTHETICAL PROTEIN ZMO_RS06210
HYPOTHETICAL PROTEIN ZMO_RS06240
HYPOTHETICAL PROTEIN ZMO_RS06245
HYPOTHETICAL PROTEIN ZMO_RS06420
HYPOTHETICAL PROTEIN ZMO_RS06530
HYPOTHETICAL PROTEIN ZMO_RS06560
HYPOTHETICAL PROTEIN ZMO_RS06580
HYPOTHETICAL PROTEIN ZMO_RS06610
HYPOTHETICAL PROTEIN ZMO_RS06640
HYPOTHETICAL PROTEIN ZMO_RS06755
HYPOTHETICAL PROTEIN ZMO_RS07230
HYPOTHETICAL PROTEIN ZMO_RS07235
HYPOTHETICAL PROTEIN ZMO_RS07305
HYPOTHETICAL PROTEIN ZMO_RS07310
HYPOTHETICAL PROTEIN ZMO_RS07325
HYPOTHETICAL PROTEIN ZMO_RS07515
HYPOTHETICAL PROTEIN ZMO_RS07650
HYPOTHETICAL PROTEIN ZMO_RS07735
HYPOTHETICAL PROTEIN ZMO_RS07785
HYPOTHETICAL PROTEIN ZMO_RS07875
HYPOTHETICAL PROTEIN ZMO_RS07880
HYPOTHETICAL PROTEIN ZMO_RS08030
HYPOTHETICAL PROTEIN ZMO_RS08045
HYPOTHETICAL PROTEIN ZMO_RS08520
HYPOTHETICAL PROTEIN ZMO_RS08580
HYPOTHETICAL PROTEIN ZMO_RS08625
HYPOTHETICAL PROTEIN ZMO_RS08720
HYPOTHETICAL PROTEIN ZMO_RS08790
HYPOTHETICAL PROTEIN ZMO_RS08835
HYPOTHETICAL PROTEIN ZMO_RS09020
IMIDAZOLE GLYCEROL PHOSPHATE SYNTHASE SUBUNIT HISF ZMO_RS06725
IMIDAZOLEGLYCEROL-PHOSPHATE DEHYDRATASE HISB ZMO_RS06740
IMP DEHYDROGENASE ZMO_RS05920
INORGANIC DIPHOSPHATASE ZMO_RS06760
INOSITOL MONOPHOSPHATASE FAMILY PROTEIN ZMO_RS06805
INOSITOL MONOPHOSPHATASE ZMO_RS01415
INTEGRATION HOST FACTOR SUBUNIT BETA ZMO_RS08100
IRON ABC TRANSPORTER PERMEASE ZMO_RS00985
IRON ABC TRANSPORTER PERMEASE ZMO_RS08310
IRON-CONTAINING ALCOHOL DEHYDROGENASE ZMO_RS07970
IRON-SULFUR CLUSTER ASSEMBLY ACCESSORY PROTEIN ZMO_RS06265
IRON-SULFUR CLUSTER ASSEMBLY SCAFFOLD PROTEIN ZMO_RS02630
KETOL-ACID REDUCTOISOMERASE ZMO_RS05105
L-2-AMINO-THIAZOLINE-4-CARBOXYLIC ACID HYDROLASE ZMO_RS01345
LACTATE UTILIZATION PROTEIN ZMO_RS00105
LARGE CONDUCTANCE MECHANOSENSITIVE CHANNEL PROTEIN MSCL ZMO_RS04675
L-ASPARTATE OXIDASE ZMO_RS00610
LEMA FAMILY PROTEIN ZMO_RS04670
LEUCINE--TRNA LIGASE ZMO_RS06425
LEUCYL AMINOPEPTIDASE FAMILY PROTEIN ZMO_RS03595
LEUCYL AMINOPEPTIDASE ZMO_RS05865
LEUCYL/PHENYLALANYL-TRNA--PROTEIN TRANSFERASE ZMO_RS04150
LIPOPOLYSACCHARIDE BIOSYNTHESIS PROTEIN ZMO_RS00980
LL-DIAMINOPIMELATE AMINOTRANSFERASE ZMO_RS07780
LON PEPTIDASE SUBSTRATE-BINDING DOMAIN-CONTAINING PROTEIN ZMO_RS07670
LOW MOLECULAR WEIGHT PHOSPHOTYROSINE PROTEIN PHOSPHATASE ZMO_RS00590
LPS EXPORT ABC TRANSPORTER PERMEASE LPTF ZMO_RS06225
LPTA/OSTA FAMILY PROTEIN ZMO_RS01205
LRP/ASNC FAMILY TRANSCRIPTIONAL REGULATOR ZMO_RS01970
LRP/ASNC FAMILY TRANSCRIPTIONAL REGULATOR ZMO_RS04950
LUD DOMAIN-CONTAINING PROTEIN ZMO_RS00100
LYSE FAMILY TRANSLOCATOR ZMO_RS00205
LYSE FAMILY TRANSLOCATOR ZMO_RS05990
LYSR FAMILY TRANSCRIPTIONAL REGULATOR ZMO_RS00210
LYSR FAMILY TRANSCRIPTIONAL REGULATOR ZMO_RS05980
LYSR FAMILY TRANSCRIPTIONAL REGULATOR ZMO_RS07805
LYSR FAMILY TRANSCRIPTIONAL REGULATOR ZMO_RS08065
LYTIC MUREIN TRANSGLYCOSYLASE ZMO_RS04860
M13 FAMILY METALLOPEPTIDASE ZMO_RS03220

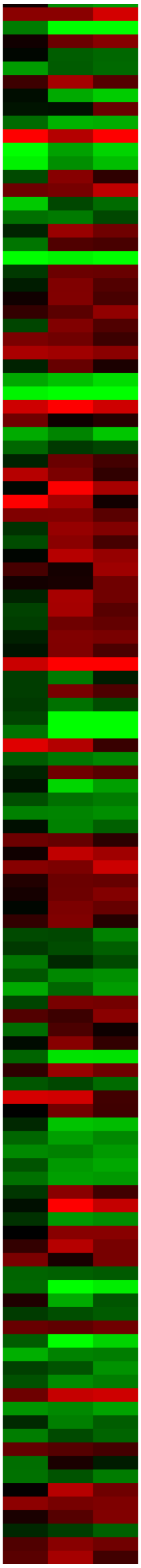

[illegible]
M20 PEPTIDASE AMINOACYLASE FAMILY PROTEIN ZMO_RS01340
M23 FAMILY METALLOPEPTIDASE ZMO_RS06270
M48 FAMILY METALLOPEPTIDASE ZMO_RS00050
MANNOSE-1-PHOSPHATE GUANYLYLTRANSFERASE/MANNOSE-6-PHOSPHATE ISOMERASE ZMO_RS05545
MARC FAMILY PROTEIN ZMO_RS01775
MCE FAMILY PROTEIN ZMO_RS04555
MECHANOSENSITIVE ION CHANNEL ZMO_RS05965
MEMBRANE LIPOPROTEIN LIPID ATTACHMENT SITE-CONTAINING PROTEIN ZMO_RS07340
MEMBRANE PROTEIN INSERTASE YIDC ZMO_RS07290
MERC DOMAIN-CONTAINING PROTEIN ZMO_RS03955
METALLOREGULATOR ARSR/SMTB FAMILY TRANSCRIPTION FACTOR ZMO_RS07860
METHIONINE ADENOSYLTRANSFERASE ZMO_RS01190
METHYL-ACCEPTING CHEMOTAXIS PROTEIN ZMO_RS00355
METHYLATED-DNA- ZMO_RS09070
METHYLENETETRAHYDROFOLATE REDUCTASE ZMO_RS07855
METHYLENETETRAHYDROFOLATE--TRNA-(URACIL(54)-C(5))-METHYLTRANSFERASE (FADH(2)-OXIDIZING) TRMFO ZMO_RS03080
MFS TRANSPORTER ZMO_RS00215
MFS TRANSPORTER ZMO_RS00410
MFS TRANSPORTER ZMO_RS06525
MFS TRANSPORTER ZMO_RS07055
MFS TRANSPORTER ZMO_RS08350
MFS TRANSPORTER ZMO_RS09025
MLIC FAMILY PROTEIN ZMO_RS04115
MODD PROTEIN ZMO_RS08270
MOLECULAR CHAPERONE DNAJ ZMO_RS02935
MOLECULAR CHAPERONE DNAK ZMO_RS02930
MONOFUNCTIONAL BIOSYNTHETIC PEPTIDOGLYCAN TRANSGLYCOSYLASE ZMO_RS04290
MOTA/TOLQ/EXBB PROTON CHANNEL FAMILY PROTEIN ZMO_RS07725
MUCR FAMILY TRANSCRIPTIONAL REGULATOR ZMO_RS06320
MULTISPECIES: HELIX-TURN-HELIX DOMAIN-CONTAINING PROTEIN ZMO_RS08820
MULTISPECIES: L-THREONINE DEHYDROGENASE ZMO_RS07165
MULTISPECIES: TRANSLATION INITIATION FACTOR IF-1 ZMO_RS04545
MUREIN TRANSGLYCOSYLASE A ZMO_RS07335
N-ACETYLGLUCOSAMINE-6-PHOSPHATE DEACETYLASE ZMO_RS04300
NAD(P)H:QUINONE OXIDOREDUCTASE ZMO_RS05975
NAD(P)H-DEPENDENT OXIDOREDUCTASE ZMO_RS08880
NAD-DEPENDENT SUCCINATE-SEMIALDEHYDE DEHYDROGENASE ZMO_RS07890
NIFU FAMILY PROTEIN ZMO_RS06335
NIFU FAMILY PROTEIN ZMO_RS08250
NIFX-ASSOCIATED NITROGEN FIXATION PROTEIN ZMO_RS08230
NITROGEN FIXATION PROTEIN NIFX ZMO_RS08225
NITROGEN FIXATION PROTEIN NIFZ ZMO_RS08180
NITROGENASE COFACTOR BIOSYNTHESIS PROTEIN NIFB ZMO_RS08170
NITROGENASE IRON PROTEIN ZMO_RS08200
NITROGENASE IRON-MOLYBDENUM COFACTOR BIOSYNTHESIS PROTEIN NIFE ZMO_RS08215
NITROGENASE IRON-MOLYBDENUM COFACTOR BIOSYNTHESIS PROTEIN NIFN ZMO_RS08220
NITROGENASE MOLYBDENUM-IRON PROTEIN ALPHA CHAIN ZMO_RS08205
NITROGENASE MOLYBDENUM-IRON PROTEIN SUBUNIT BETA ZMO_RS08210
NITRONATE MONOOXYGENASE ZMO_RS08400
NUCLEAR TRANSPORT FACTOR 2 FAMILY PROTEIN ZMO_RS01010
NUCLEOSIDE TRANSPORTER ZMO_RS04325
NUCLEOTIDYLTRANSFERASE FAMILY PROTEIN ZMO_RS04925
OMPA FAMILY PROTEIN ZMO_RS06055
OMPH FAMILY OUTER MEMBRANE PROTEIN ZMO_RS05135
ORGANIC HYDROPEROXIDE RESISTANCE PROTEIN ZMO_RS03090
OROTIDINE-5\\'-PHOSPHATE DECARBOXYLASE ZMO_RS02610
OUTER MEMBRANE BETA-BARREL PROTEIN ZMO_RS06155
OUTER MEMBRANE BETA-BARREL PROTEIN ZMO_RS06675
OUTER MEMBRANE PROTEIN ASSEMBLY FACTOR BAMA ZMO_RS05140
OUTER MEMBRANE PROTEIN ASSEMBLY FACTOR BAMD ZMO_RS07910
OUTER MEMBRANE PROTEIN ASSEMBLY FACTOR BAME ZMO_RS08660
OXIDOREDUCTASE ZMO_RS08295
PAAI FAMILY THIOESTERASE ZMO_RS08890
PANTOATE--BETA-ALANINE LIGASE ZMO_RS09000
PARA FAMILY PROTEIN ZMO_RS09035
PAS-DOMAIN CONTAINING PROTEIN ZMO_RS04935
PBSX FAMILY PHAGE TERMINASE LARGE SUBUNIT ZMO_RS01630
PENICILLIN-BINDING PROTEIN 2 ZMO_RS01535
PENICILLIN-BINDING PROTEIN 2 ZMO_RS03685
PEPTIDE CHAIN RELEASE FACTOR 2 ZMO_RS00850
PEPTIDOGLYCAN DD-METALLOENDOPEPTIDASE FAMILY PROTEIN ZMO_RS07485
PEPTIDYLPROLYL ISOMERASE ZMO_RS02005
PEPTIDYLPROLYL ISOMERASE ZMO_RS03445
PHAGE MAJOR TAIL TUBE PROTEIN ZMO_RS00285
PHAGE SHOCK PROTEIN PSPA ZMO_RS04755
PHOSPHATASE PAP2 FAMILY PROTEIN ZMO_RS00545
PHOSPHATASE PAP2 FAMILY PROTEIN ZMO_RS02230
PHOSPHATE ACYLTRANSFERASE PLSX ZMO_RS08620
PHOSPHATE REGULON TRANSCRIPTIONAL REGULATOR PHOB ZMO_RS05215
PHOSPHOENOLPYRUVATE CARBOXYLASE ZMO_RS06705
PHOSPHOGLUCONATE DEHYDRATASE ZMO_RS01575
PHOSPHO-N-ACETYLMURAMOYL-PENTAPEPTIDE-TRANSFERASE ZMO_RS03700
PHOSPHORIBOSYLAMINOIMIDAZOLESUCCINOCARBOXAMIDE SYNTHASE ZMO_RS04710
PHOSPHORIBOSYLANTHRANILATE ISOMERASE ZMO_RS02605
PHOSPHORIBOSYL-ATP DIPHOSPHATASE ZMO_RS06720
PHOSPHORIBOSYLTRANSFERASE ZMO_RS02910
PHOSPHOSERINE TRANSAMINASE ZMO_RS07570
PHOSPHOTRANSFERASE ZMO_RS06170
PHZF FAMILY PHENAZINE BIOSYNTHESIS PROTEIN ZMO_RS08860
P-II FAMILY NITROGEN REGULATOR ZMO_RS02130
PLP-DEPENDENT AMINOTRANSFERASE FAMILY PROTEIN ZMO_RS08340
PLP-DEPENDENT AMINOTRANSFERASE FAMILY PROTEIN ZMO_RS08855
POLYISOPRENOID-BINDING PROTEIN ZMO_RS05970
POLYPRENYL SYNTHETASE FAMILY PROTEIN ZMO_RS03820
POLYRIBONUCLEOTIDE NUCLEOTIDYLTRANSFERASE ZMO_RS02430
PORIN FAMILY PROTEIN ZMO_RS06680
PORPHOBILINOGEN SYNTHASE ZMO_RS08515
PQQ-DEPENDENT SUGAR DEHYDROGENASE ZMO_RS02475
PREPROTEIN TRANSLOCASE SUBUNIT SECE ZMO_RS03230
PREPROTEIN TRANSLOCASE SUBUNIT SECG ZMO_RS01995
PREPROTEIN TRANSLOCASE SUBUNIT SECY ZMO_RS02360
PREPROTEIN TRANSLOCASE SUBUNIT YAJC ZMO_RS08605
PRIMOSOMAL PROTEIN N\\' ZMO_RS08725
PROLINE--TRNA LIGASE ZMO_RS01985
PROTEIN TRANSLOCASE SUBUNIT SECF ZMO_RS08595
PSEUDOURIDINE SYNTHASE ZMO_RS02570
PTS SUGAR TRANSPORTER SUBUNIT IIA ZMO_RS05945
PURINE NUCLEOSIDE PERMEASE ZMO_RS06505
PYRIDOXAL PHOSPHATE-DEPENDENT AMINOTRANSFERASE ZMO_RS01455
PYRIDOXAL PHOSPHATE-DEPENDENT AMINOTRANSFERASE ZMO_RS08885
PYRIDOXINE 5\\'-PHOSPHATE SYNTHASE ZMO_RS07685
PYRROLINE-5-CARBOXYLATE REDUCTASE ZMO_RS01335
PYRUVATE DEHYDROGENASE COMPLEX E1 COMPONENT SUBUNIT BETA ZMO_RS07205
PYRUVATE FORMATE LYASE-ACTIVATING PROTEIN ZMO_RS07010
QUINONE OXIDOREDUCTASE ZMO_RS09080

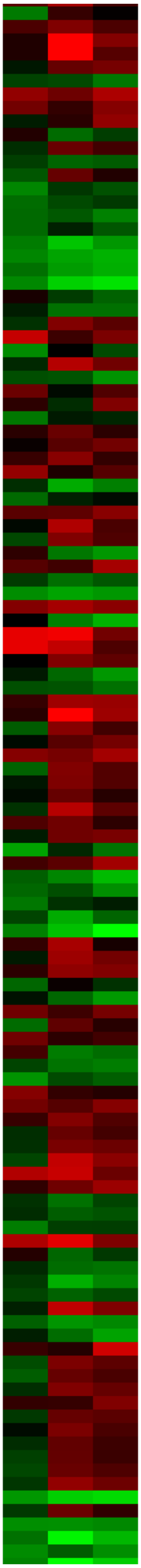

[illegible]
RDGB/HAM1 FAMILY NON-CANONICAL PURINE NTP PYROPHOSPHATASE ZMO_RS00065
REDOX-REGULATED ATPASE YCHF ZMO_RS08680
RELAXASE/MOBILIZATION NUCLEASE AND DUF3363 DOMAIN-CONTAINING PROTEIN ZMO_RS08850
REPLICATION INITIATOR PROTEIN A ZMO_RS09215
REPLICATION-ASSOCIATED RECOMBINATION PROTEIN A ZMO_RS00510
RESPONSE REGULATOR TRANSCRIPTION FACTOR ZMO_RS02055
RESPONSE REGULATOR TRANSCRIPTION FACTOR ZMO_RS06195
RESPONSE REGULATOR ZMO_RS00325
RESPONSE REGULATOR ZMO_RS01080
RESPONSE REGULATOR ZMO_RS06280
RESTRICTION ENDONUCLEASE ZMO_RS00135
RHOMBOID FAMILY INTRAMEMBRANE SERINE PROTEASE ZMO_RS07245
RIBBON-HELIX-HELIX DOMAIN-CONTAINING PROTEIN ZMO_RS01305
RIBONUCLEASE D ZMO_RS03185
RIBONUCLEASE E/G ZMO_RS00830
RIBONUCLEASE HI ZMO_RS07185
RIBONUCLEASE PH ZMO_RS00070
RIBONUCLEOSIDE TRIPHOSPHATE REDUCTASE ZMO_RS04595
RIBONUCLEOSIDE-DIPHOSPHATE REDUCTASE SUBUNIT ALPHA ZMO_RS04645
RIBOSE 5-PHOSPHATE ISOMERASE B ZMO_RS05385
RIBOSE-PHOSPHATE PYROPHOSPHOKINASE ZMO_RS06810
RIBOSOME RECYCLING FACTOR ZMO_RS05165
RIBOSOME SILENCING FACTOR ZMO_RS07475
RIBOSOME-ASSOCIATED ATPASE/PUTATIVE TRANSPORTER RBBA ZMO_RS03565
RIBOSOME-ASSOCIATED TRANSLATION INHIBITOR RAIA ZMO_RS00175
RIBULOSE-PHOSPHATE 3-EPIMERASE ZMO_RS00090
RIDA FAMILY PROTEIN ZMO_RS08870
RIP METALLOPROTEASE RSEP ZMO_RS05145
RLUA FAMILY PSEUDOURIDINE SYNTHASE ZMO_RS03355
RNA CHAPERONE HFQ ZMO_RS01480
RNA METHYLTRANSFERASE ZMO_RS05400
RNA METHYLTRANSFERASE ZMO_RS05955
RNA POLYMERASE FACTOR SIGMA-54 ZMO_RS01195
RNA POLYMERASE SIGMA FACTOR RPOD ZMO_RS07275
RNA POLYMERASE SIGMA FACTOR RPOH ZMO_RS03350
RNA POLYMERASE-BINDING PROTEIN DKSA ZMO_RS04425
RNA PSEUDOURIDINE SYNTHASE ZMO_RS02190
RNASE ADAPTER RAPZ ZMO_RS05940
RNFABCDGE TYPE ELECTRON TRANSPORT COMPLEX SUBUNIT B ZMO_RS08150
ROD SHAPE-DETERMINING PROTEIN RODA ZMO_RS01540
ROD-BINDING PROTEIN ZMO_RS02695
ROK FAMILY PROTEIN ZMO_RS07740
S41 FAMILY PEPTIDASE ZMO_RS07490
SAPC FAMILY PROTEIN ZMO_RS04515
SCP2 STEROL-BINDING DOMAIN-CONTAINING PROTEIN ZMO_RS03375
SDR FAMILY OXIDOREDUCTASE ZMO_RS00380
SDR FAMILY OXIDOREDUCTASE ZMO_RS01330
SDR FAMILY OXIDOREDUCTASE ZMO_RS01360
SDR FAMILY OXIDOREDUCTASE ZMO_RS01450
SDR FAMILY OXIDOREDUCTASE ZMO_RS06130
SDR FAMILY OXIDOREDUCTASE ZMO_RS06970
SDR FAMILY OXIDOREDUCTASE ZMO_RS07050
SDR FAMILY OXIDOREDUCTASE ZMO_RS08865
SEL1 REPEAT FAMILY PROTEIN ZMO_RS00125
SEL1 REPEAT FAMILY PROTEIN ZMO_RS00200
SEL1 REPEAT FAMILY PROTEIN ZMO_RS00560
SEL1 REPEAT FAMILY PROTEIN ZMO_RS00565
SEL1 REPEAT FAMILY PROTEIN ZMO_RS00570
SEL1-LIKE REPEAT PROTEIN ZMO_RS00280
SEL1-LIKE REPEAT PROTEIN ZMO_RS00580
SEPTAL RING LYTIC TRANSGLYCOSYLASE RLPA FAMILY PROTEIN ZMO_RS04865
SEPTUM FORMATION PROTEIN MAF ZMO_RS00195
SEPTUM FORMATION PROTEIN MAF ZMO_RS04540
SERINE ACETYLTRANSFERASE ZMO_RS07795
SERINE HYDROXYMETHYLTRANSFERASE ZMO_RS05390
SHIKIMATE KINASE ZMO_RS02645
SIGNAL PEPTIDE PEPTIDASE SPPA ZMO_RS08750
SIGNAL RECOGNITION PARTICLE PROTEIN ZMO_RS04805
SIMPL DOMAIN-CONTAINING PROTEIN ZMO_RS01245
SINGLE-STRANDED DNA-BINDING PROTEIN ZMO_RS06900
SINGLE-STRANDED-DNA-SPECIFIC EXONUCLEASE RECJ ZMO_RS05530
SMP-30/GLUCONOLACTONASE/LRE FAMILY PROTEIN ZMO_RS09050
SMR/MUTS FAMILY PROTEIN ZMO_RS07330
SORBITOL DEHYDROGENASE FAMILY PROTEIN ZMO_RS03505
SOXR REDUCING SYSTEM RSEC FAMILY PROTEIN ZMO_RS08285
SPOR DOMAIN-CONTAINING PROTEIN ZMO_RS03580
SPOR DOMAIN-CONTAINING PROTEIN ZMO_RS03750
SSRA-BINDING PROTEIN SMPB ZMO_RS03215
SUCCINATE DEHYDROGENASE CYTOCHROME B SUBUNIT ZMO_RS02530
SUCCINYL-DIAMINOPIMELATE DESUCCINYLASE ZMO_RS07320
SUCROSE-6-PHOSPHATE HYDROLASE ZMO_RS04220
SUF SYSTEM FE-S CLUSTER ASSEMBLY PROTEIN ZMO_RS01845
SUFD FAMILY FE-S CLUSTER ASSEMBLY PROTEIN ZMO_RS01835
SUGAR PORTER FAMILY MFS TRANSPORTER ZMO_RS01265
SULFATE ADENYLYLTRANSFERASE SUBUNIT CYSN ZMO_RS00020
SULFITE EXPORTER TAUE/SAFE FAMILY PROTEIN ZMO_RS00230
SUPEROXIDE DISMUTASE ZMO_RS04740
TAIL FIBER DOMAIN-CONTAINING PROTEIN ZMO_RS01710
TATD FAMILY HYDROLASE ZMO_RS04890
TETRATRICOPEPTIDE REPEAT PROTEIN ZMO_RS01945
TETRATRICOPEPTIDE REPEAT PROTEIN ZMO_RS03380
TETRATRICOPEPTIDE REPEAT PROTEIN ZMO_RS07675
THIOREDOXIN ZMO_RS04910
THIOREDOXIN-DISULFIDE REDUCTASE ZMO_RS05110
THREONINE SYNTHASE ZMO_RS08575
THREONINE--TRNA LIGASE ZMO_RS03415
TOLC FAMILY OUTER MEMBRANE PROTEIN ZMO_RS01095
TOLL/INTERLEUKIN-1 RECEPTOR DOMAIN-CONTAINING PROTEIN ZMO_RS07135
TOL-PAL SYSTEM PROTEIN TOLB ZMO_RS00695
TONB FAMILY PROTEIN ZMO_RS04750
TONB-DEPENDENT RECEPTOR ZMO_RS00005
TONB-DEPENDENT RECEPTOR ZMO_RS00150
TONB-DEPENDENT RECEPTOR ZMO_RS00540
TONB-DEPENDENT RECEPTOR ZMO_RS03520
TONB-DEPENDENT RECEPTOR ZMO_RS03545
TONB-DEPENDENT RECEPTOR ZMO_RS04025
TONB-DEPENDENT RECEPTOR ZMO_RS06600
TONB-DEPENDENT RECEPTOR ZMO_RS07005
TONB-DEPENDENT RECEPTOR ZMO_RS08160
TONB-DEPENDENT SIDEROPHORE RECEPTOR ZMO_RS07315
TRANSCRIPTION ELONGATION FACTOR GREA ZMO_RS07250
TRANSCRIPTION ELONGATION FACTOR GREB ZMO_RS03200
TRANSCRIPTION TERMINATION FACTOR RHO ZMO_RS09085
TRANSCRIPTION TERMINATION/ANTITERMINATION FACTOR NUSG ZMO_RS03235
TRANSCRIPTIONAL REPRESSOR NRDR ZMO_RS05395
[illegible]

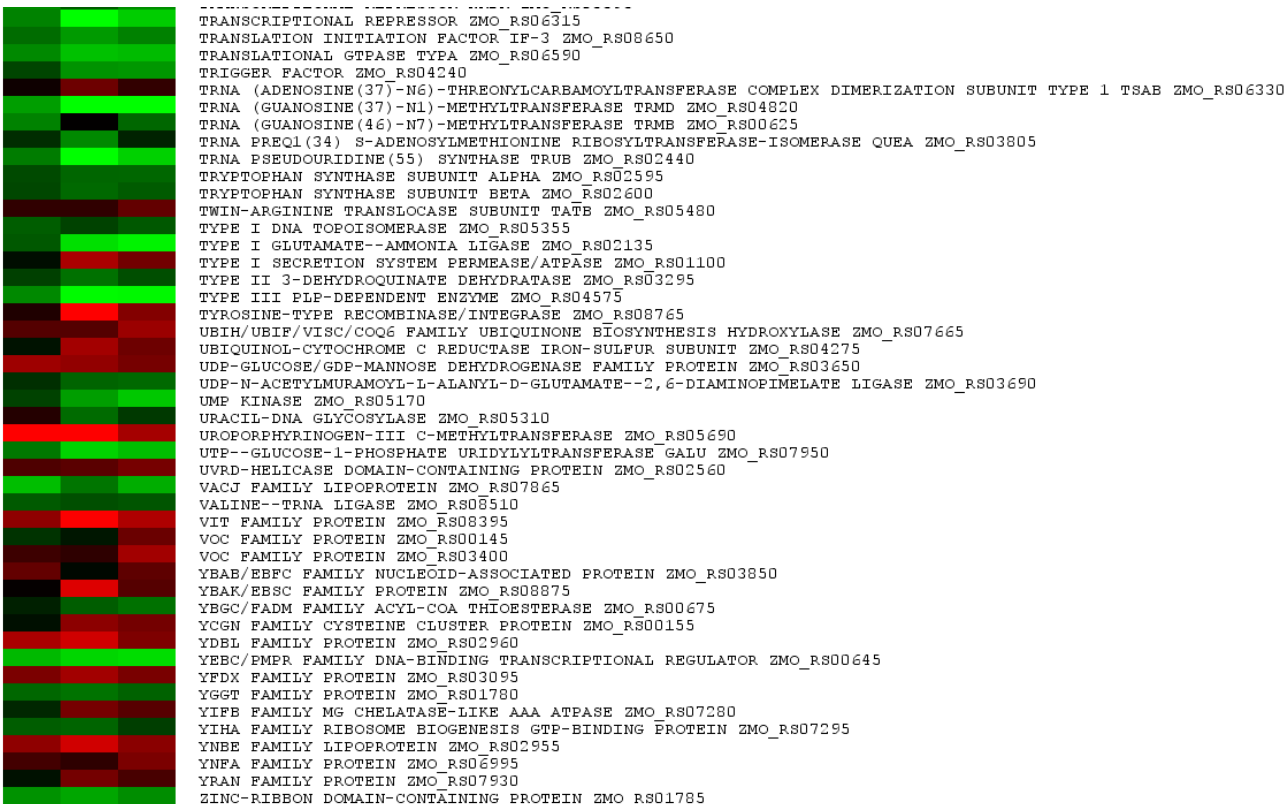

TRANSCRIPTIONAL REPRESSOR ZMO_RS06315
TRANSLATION INITIATION FACTOR IF-3 ZMO_RS08650
TRANSLATIONAL GTPASE TYPA ZMO_RS06590
TRIGGER FACTOR ZMO_RS04240
TRNA (ADENOSINE(37)-N6)-THREONYLCARBAMOYLTRANSFERASE COMPLEX DIMERIZATION SUBUNIT TYPE 1 TSAB ZMO_RS06330
TRNA (GUANOSINE(37)-N1)-METHYLTRANSFERASE TRMD ZMO_RS04820
TRNA (GUANOSINE(46)-N7)-METHYLTRANSFERASE TRMB ZMO_RS00625
TRNA PREQ1(34) S-ADENOSYLMETHIONINE RIBOSYLTRANSFERASE-ISOMERASE QUEA ZMO_RS03805
TRNA PSEUDOURIDINE(55) SYNTHASE TRUB ZMO_RS02440
TRYPTOPHAN SYNTHASE SUBUNIT ALPHA ZMO_RS02595
TRYPTOPHAN SYNTHASE SUBUNIT BETA ZMO_RS02600
TWIN-ARGININE TRANSLOCASE SUBUNIT TATB ZMO_RS05480
TYPE I DNA TOPOISOMERASE ZMO_RS05355
TYPE I GLUTAMATE--AMMONIA LIGASE ZMO_RS02135
TYPE I SECRETION SYSTEM PERMEASE/ATPASE ZMO_RS01100
TYPE II 3-DEHYDROQUINATE DEHYDRATASE ZMO_RS03295
TYPE III PLP-DEPENDENT ENZYME ZMO_RS04575
TYROSINE-TYPE RECOMBINASE/INTEGRASE ZMO_RS08765
UBIH/UBIF/VISC/COQ6 FAMILY UBIQUINONE BIOSYNTHESIS HYDROXYLASE ZMO_RS07665
UBIQUINOL-CYTOCHROME C REDUCTASE IRON-SULFUR SUBUNIT ZMO_RS04275
UDP-GLUCOSE/GDP-MANNOSE DEHYDROGENASE FAMILY PROTEIN ZMO_RS03650
UDP-N-ACETYLMURAMOYL-L-ALANYL-D-GLUTAMATE--2,6-DIAMINOPIMELATE LIGASE ZMO_RS03690
UMP KINASE ZMO_RS05170
URACIL-DNA GLYCOSYLASE ZMO_RS05310
UROPORPHYRINOGEN-III C-METHYLTRANSFERASE ZMO_RS05690
UTP--GLUCOSE-1-PHOSPHATE URIDYLYLTRANSFERASE GALU ZMO_RS07950
UVRD-HELICASE DOMAIN-CONTAINING PROTEIN ZMO_RS02560
VACJ FAMILY LIPOPROTEIN ZMO_RS07865
VALINE--TRNA LIGASE ZMO_RS08510
VIT FAMILY PROTEIN ZMO_RS08395
VOC FAMILY PROTEIN ZMO_RS00145
VOC FAMILY PROTEIN ZMO_RS03400
YBAB/EBFC FAMILY NUCLEOID-ASSOCIATED PROTEIN ZMO_RS03850
YBAK/EBSC FAMILY PROTEIN ZMO_RS08875
YBGC/FADM FAMILY ACYL-COA THIOESTERASE ZMO_RS00675
YCGN FAMILY CYSTEINE CLUSTER PROTEIN ZMO_RS00155
YDBL FAMILY PROTEIN ZMO_RS02960
YEBC/PMPR FAMILY DNA-BINDING TRANSCRIPTIONAL REGULATOR ZMO_RS00645
YFDX FAMILY PROTEIN ZMO_RS03095
YGGT FAMILY PROTEIN ZMO_RS01780
YIFB FAMILY MG CHELATASE-LIKE AAA ATPASE ZMO_RS07280
YIHA FAMILY RIBOSOME BIOGENESIS GTP-BINDING PROTEIN ZMO_RS07295
YNBE FAMILY LIPOPROTEIN ZMO_RS02955
YNFA FAMILY PROTEIN ZMO_RS06995
YRAN FAMILY PROTEIN ZMO_RS07930
ZINC-RIBBON DOMAIN-CONTAINING PROTEIN ZMO RS01785
