# Supplementary material for: The Quorum Sensing Auto-Inducer 2 (AI-2) Stimulates Nitrogen Fixation and Favors Ethanol Production over Biomass Accumulation in Zymomonas mobilis
Source: Int J Mol Sci. 2021 May 26;22(11):5628. doi: 10.3390/ijms22115628 (PMC8198075; doi:10.3390/ijms22115628)
Supplement: Supplementary file 1 [file ijms-22-05628-s001.zip › Supplementary/Supplementary Material S2.pdf]

Supplementary Material S2 - Comparison of relative expression data obtained by RNA-seq and quantitative real-time PCR (qPCR). To assess the reliability of the RNA-seq results, 20 genes were selected to have their relative expression ratios evaluated independently, by qPCR (see text for details). Qualitatively coincident comparisons are highlighted in green, while discrepancies are highlighted in red.

Alencar *et al*. (2021). The quorum sensing auto-inducer 2 (AI-2) stimulates nitrogen fixation and favors ethanol production over biomass accumulation in *Zymomonas mobilis*. *Int. J. Mol. Sci. Submitted.*

LocusTag_ID	Description	14h x 20h		14h x 16h+AI-2		14h x 20h+AI-2	
		RNA-seq	qPCR	RNA-seq	qPCR	RNA-seq	qPCR
ZMO_RS00220	alpha/beta hydrolase	-0.58	-7.36	0.9	0.73	0.82	7.86
ZMO_RS05560	alcohol dehydrogenase	2.02	2.21	1.06	3.5	1.13	1.82
ZMO_RS08755	co-chaperonin GroES	-0.65	-1.81	0.83	1.2	-1.63	-1.83
ZMO_RS00205	lysine exporter protein	-0.42	-3.15	0.25	1.47	0.99	-1.85
ZMO_RS08485	glutaredoxin-like protein	0.32	0.76	0.64	0.04	0.13	0.71
ZMO_RS05980	LysR family transcriptional regulator	-0.07	2.83	0.97	1.98	0.24	-1.73
ZMO_RS04025	TonB-dependent receptor	-0.07	-2.81	0.73	2.32	0.44	0.48
ZMO_RS00175	sigma 54 modulation protein/ribosomal protein S30EA	1.17	0.2	0.36	2.53	0.73	-0.42
ZMO_RS07220	phosphopyruvate hydratase	-0.25	-2.2	-0.84	0.64	-1.16	-2.94
ZMO_RS02370	30S ribosomal protein S13	-1.26	-4.62	-1.93	-1.51	-1.99	-5.29
ZMO_RS06270	peptidase M23	-0.73	-0.76	-1.59	0.41	-1.76	-0.52
ZMO_RS04470	5-methyl-tetrahydropteroyltriglutamate--homocysteine S-methyltransferase	-1.91	-3.57	-1.49	-0.2	-2.25	-4.1
ZMO_RS06320	MucR family transcriptional regulator	-1.44	-2.84	-2.28	-1.04	-1.97	-3.99
ZMO_RS02245	elongation factor G	-0.56	1.29	-1.89	-0.56	-1.61	-0.29
ZMO_RS00755	glyceraldehyde-3-phosphate dehydrogenase, type I	0.24	0.43	-0.49	0.66	-0.52	-0.56
ZMO_RS03200	transcription elongation factor GreB	-0.34	10.45	0.63	6.13	0.29	10.17
ZMO_RS04395	ABC transporter-like protein	-0.46	2.37	0.13	0.64	0.12	6.81
ZMO_RS01520	rod shape-determining protein MreB	-0.13	-0.03	-0.27	0.15	-0.38	-3.78
ZMO_RS08860	PhzF family phenazine biosynthesis protein	-0.09	-4.11	1.64	1.2	1.16	9.24
ZMO_RS01575	phosphogluconate dehydratase	1.26	1.63	1.23	1.62	0.39	0.88
