# Supplementary material for: The Quorum Sensing Auto-Inducer 2 (AI-2) Stimulates Nitrogen Fixation and Favors Ethanol Production over Biomass Accumulation in Zymomonas mobilis
Source: Int J Mol Sci. 2021 May 26;22(11):5628. doi: 10.3390/ijms22115628 (PMC8198075; doi:10.3390/ijms22115628)
Supplement: Supplementary file 1 [file ijms-22-05628-s001.zip › Supplementary/Supplementary Material S3.pdf]

Supplementary Material S3 – Expression patterns of the 724 *Z. mobilis* genes identified as modulated in response to AI-2, distributed into different categories/subcategories, as defined by the *OMICS Dashboard* tool, available from the *Pathway Tools* software package. The total modulation in each category is shown as the sum of expression ratios (Log-transformed) involving all genes present in the category in question.

Alencar *et al*. (2021). The quorum sensing auto-inducer 2 (AI-2) stimulates nitrogen fixation and favors ethanol production over biomass accumulation in *Zymomonas mobilis*. *Int. J. Mol. Sci. Submitted.*

Supplementary Material S3A –Central Dogma

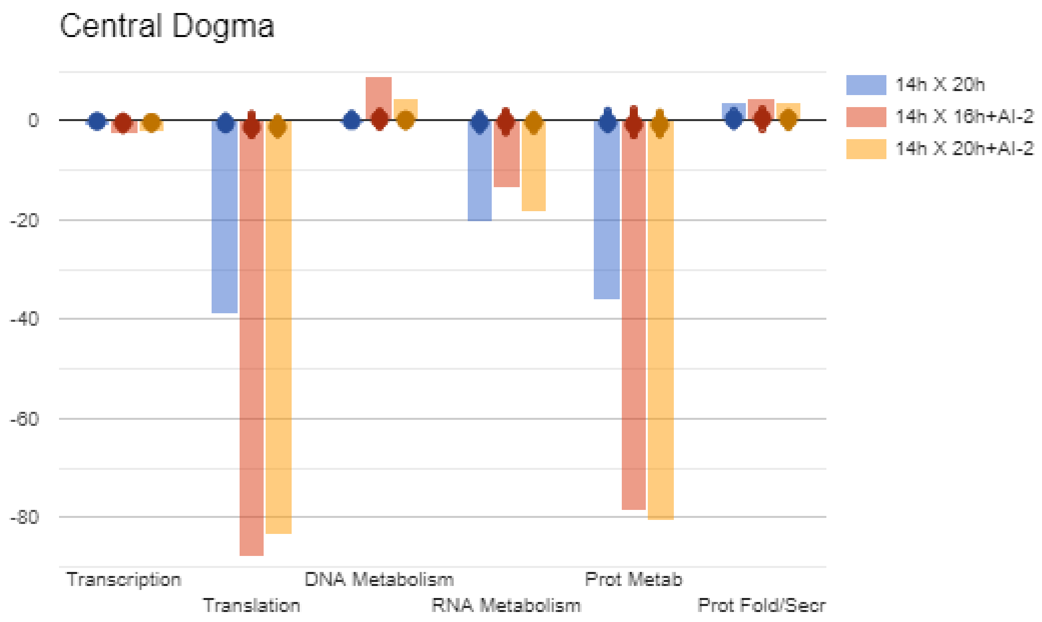

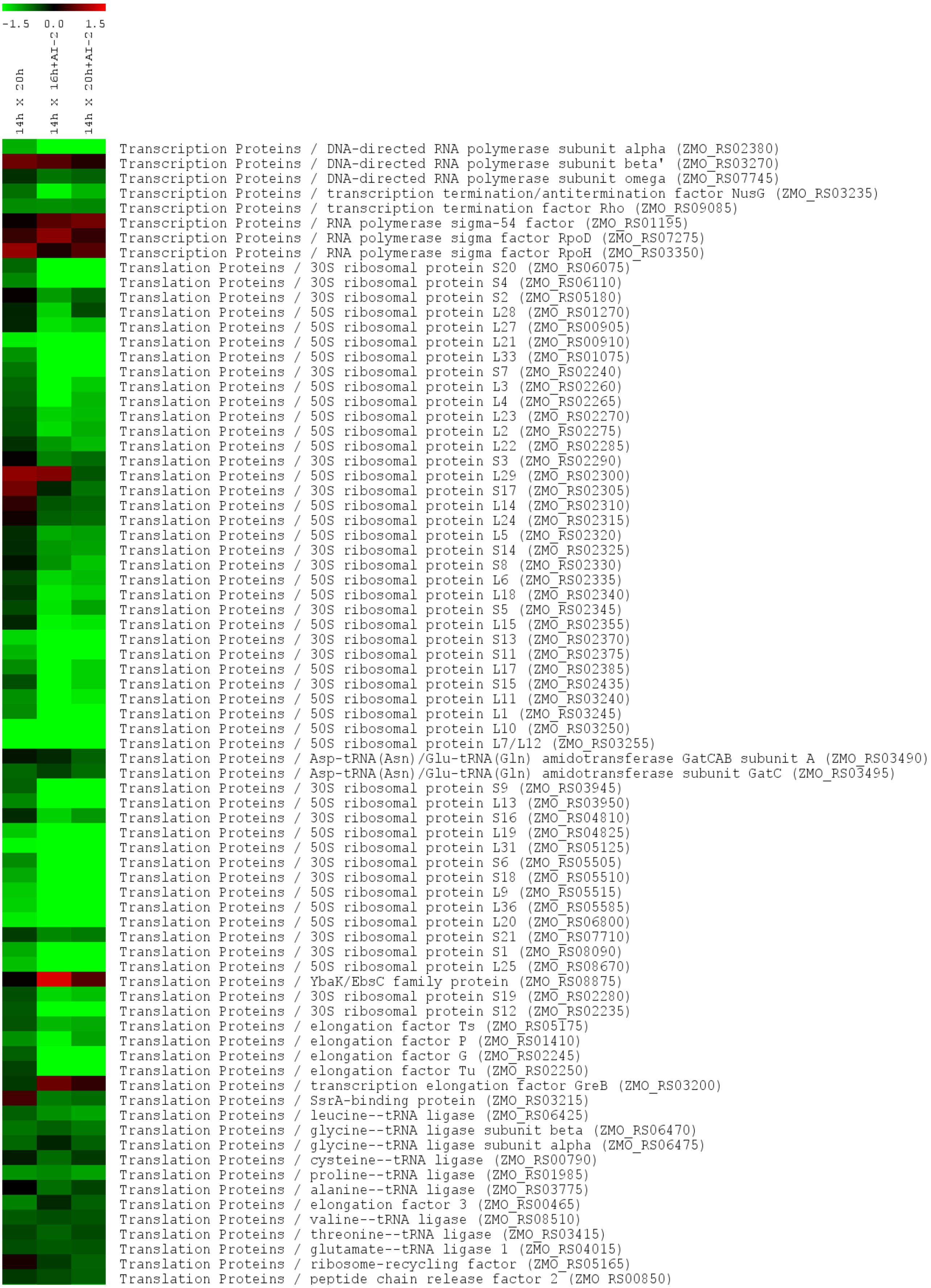

-1.5 0.0 1.5
14h X 20h
14h X 16h+AI-2
14h X 20h+AI-2
Transcription Proteins / DNA-directed RNA polymerase subunit alpha (ZMO_RS02380)
Transcription Proteins / DNA-directed RNA polymerase subunit beta' (ZMO_RS03270)
Transcription Proteins / DNA-directed RNA polymerase subunit omega (ZMO_RS07745)
Transcription Proteins / transcription termination/antitermination factor NusG (ZMO_RS03235)
Transcription Proteins / transcription termination factor Rho (ZMO_RS09085)
Transcription Proteins / RNA polymerase sigma-54 factor (ZMO_RS01195)
Transcription Proteins / RNA polymerase sigma factor RpoD (ZMO_RS07275)
Transcription Proteins / RNA polymerase sigma factor RpoH (ZMO_RS03350)
Translation Proteins / 30S ribosomal protein S20 (ZMO_RS06075)
Translation Proteins / 30S ribosomal protein S4 (ZMO_RS06110)
Translation Proteins / 30S ribosomal protein S2 (ZMO_RS05180)
Translation Proteins / 50S ribosomal protein L28 (ZMO_RS01270)
Translation Proteins / 50S ribosomal protein L27 (ZMO_RS00905)
Translation Proteins / 50S ribosomal protein L21 (ZMO_RS00910)
Translation Proteins / 50S ribosomal protein L33 (ZMO_RS01075)
Translation Proteins / 30S ribosomal protein S7 (ZMO_RS02240)
Translation Proteins / 50S ribosomal protein L3 (ZMO_RS02260)
Translation Proteins / 50S ribosomal protein L4 (ZMO_RS02265)
Translation Proteins / 50S ribosomal protein L23 (ZMO_RS02270)
Translation Proteins / 50S ribosomal protein L2 (ZMO_RS02275)
Translation Proteins / 50S ribosomal protein L22 (ZMO_RS02285)
Translation Proteins / 30S ribosomal protein S3 (ZMO_RS02290)
Translation Proteins / 50S ribosomal protein L29 (ZMO_RS02300)
Translation Proteins / 30S ribosomal protein S17 (ZMO_RS02305)
Translation Proteins / 50S ribosomal protein L14 (ZMO_RS02310)
Translation Proteins / 50S ribosomal protein L24 (ZMO_RS02315)
Translation Proteins / 50S ribosomal protein L5 (ZMO_RS02320)
Translation Proteins / 30S ribosomal protein S14 (ZMO_RS02325)
Translation Proteins / 30S ribosomal protein S8 (ZMO_RS02330)
Translation Proteins / 50S ribosomal protein L6 (ZMO_RS02335)
Translation Proteins / 50S ribosomal protein L18 (ZMO_RS02340)
Translation Proteins / 30S ribosomal protein S5 (ZMO_RS02345)
Translation Proteins / 50S ribosomal protein L15 (ZMO_RS02355)
Translation Proteins / 30S ribosomal protein S13 (ZMO_RS02370)
Translation Proteins / 30S ribosomal protein S11 (ZMO_RS02375)
Translation Proteins / 50S ribosomal protein L17 (ZMO_RS02385)
Translation Proteins / 30S ribosomal protein S15 (ZMO_RS02435)
Translation Proteins / 50S ribosomal protein L11 (ZMO_RS03240)
Translation Proteins / 50S ribosomal protein L1 (ZMO_RS03245)
Translation Proteins / 50S ribosomal protein L10 (ZMO_RS03250)
Translation Proteins / 50S ribosomal protein L7/L12 (ZMO_RS03255)
Translation Proteins / Asp-tRNA(Asn)/Glu-tRNA(Gln) amidotransferase GatCAB subunit A (ZMO_RS03490)
Translation Proteins / Asp-tRNA(Asn)/Glu-tRNA(Gln) amidotransferase subunit GatC (ZMO_RS03495)
Translation Proteins / 30S ribosomal protein S9 (ZMO_RS03945)
Translation Proteins / 50S ribosomal protein L13 (ZMO_RS03950)
Translation Proteins / 30S ribosomal protein S16 (ZMO_RS04810)
Translation Proteins / 50S ribosomal protein L19 (ZMO_RS04825)
Translation Proteins / 50S ribosomal protein L31 (ZMO_RS05125)
Translation Proteins / 30S ribosomal protein S6 (ZMO_RS05505)
Translation Proteins / 30S ribosomal protein S18 (ZMO_RS05510)
Translation Proteins / 50S ribosomal protein L9 (ZMO_RS05515)
Translation Proteins / 50S ribosomal protein L36 (ZMO_RS05585)
Translation Proteins / 50S ribosomal protein L20 (ZMO_RS06800)
Translation Proteins / 30S ribosomal protein S21 (ZMO_RS07710)
Translation Proteins / 30S ribosomal protein S1 (ZMO_RS08090)
Translation Proteins / 50S ribosomal protein L25 (ZMO_RS08670)
Translation Proteins / YbaK/EbsC family protein (ZMO_RS08875)
Translation Proteins / 30S ribosomal protein S19 (ZMO_RS02280)
Translation Proteins / 30S ribosomal protein S12 (ZMO_RS02235)
Translation Proteins / elongation factor Ts (ZMO_RS05175)
Translation Proteins / elongation factor P (ZMO_RS01410)
Translation Proteins / elongation factor G (ZMO_RS02245)
Translation Proteins / elongation factor Tu (ZMO_RS02250)
Translation Proteins / transcription elongation factor GreB (ZMO_RS03200)
Translation Proteins / SsrA-binding protein (ZMO_RS03215)
Translation Proteins / leucine--tRNA ligase (ZMO_RS06425)
Translation Proteins / glycine--tRNA ligase subunit beta (ZMO_RS06470)
Translation Proteins / glycine--tRNA ligase subunit alpha (ZMO_RS06475)
Translation Proteins / cysteine--tRNA ligase (ZMO_RS00790)
Translation Proteins / proline--tRNA ligase (ZMO_RS01985)
Translation Proteins / alanine--tRNA ligase (ZMO_RS03775)
Translation Proteins / elongation factor 3 (ZMO_RS00465)
Translation Proteins / valine--tRNA ligase (ZMO_RS08510)
Translation Proteins / threonine--tRNA ligase (ZMO_RS03415)
Translation Proteins / glutamate--tRNA ligase 1 (ZMO_RS04015)
Translation Proteins / ribosome-recycling factor (ZMO_RS05165)
Translation Proteins / peptide chain release factor 2 (ZMO_RS00850)

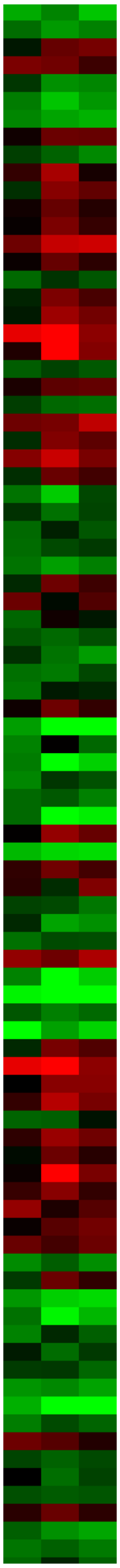

Translation Proteins / translation initiation factor IF-1 (ZMO_RS04545)
Translation Proteins / translation initiation factor IF-3 (ZMO_RS08650)
DNA Metabolism / replication-associated recombination protein A (ZMO_RS00510)
DNA Metabolism / molecular chaperone DnaJ (ZMO_RS02935)
DNA Metabolism / DNA polymerase III subunit beta (ZMO_RS04390)
DNA Metabolism / ribonucleoside triphosphate reductase (ZMO_RS04595)
DNA Metabolism / ribonucleotide-diphosphate reductase subunit alpha (ZMO_RS04645)
DNA Metabolism / DNA polymerase III subunit chi (ZMO_RS05860)
DNA Metabolism / DNA polymerase III subunit delta (ZMO_RS06415)
DNA Metabolism / single-stranded DNA-binding protein (ZMO_RS06900)
DNA Metabolism / DNA replication and repair protein RecF (ZMO_RS07115)
DNA Metabolism / DNA topoisomerase (ATP-hydrolyzing) subunit B (ZMO_RS07110)
DNA Metabolism / DNA primase (ZMO_RS07270)
DNA Metabolism / primosomal protein N' (ZMO_RS08725)
DNA Metabolism / DNA recombination protein RmuC (ZMO_RS03630)
DNA Metabolism / crossover junction endodeoxyribonuclease RuvC (ZMO_RS00650)
DNA Metabolism / Holliday junction branch migration protein RuvA (ZMO_RS00655)
DNA Metabolism / single-stranded-DNA-specific exonuclease RecJ (ZMO_RS05530)
DNA Metabolism / integration host factor subunit beta (ZMO_RS08100)
DNA Metabolism / DUF4102 domain-containing protein (ZMO_RS08765)
DNA Metabolism / DNA topoisomerase 1 (ZMO_RS05355)
DNA Metabolism / DNA topoisomerase IV subunit B (ZMO_RS01770)
DNA Metabolism / exodeoxyribonuclease III (ZMO_RS06260)
DNA Metabolism / methylated-DNA--[protein]-cysteine S-methyltransferase (ZMO_RS09070)
DNA Metabolism / DNA-3-methyladenine glycosylase 2 family protein (ZMO_RS01620)
DNA Metabolism / excinuclease ABC subunit B (ZMO_RS01550)
DNA Metabolism / restriction endonuclease (ZMO_RS00135)
DNA Metabolism / exodeoxyribonuclease 7 small subunit (ZMO_RS03825)
DNA Metabolism / TatD family deoxyribonuclease (ZMO_RS04890)
RNA Metabolism / ribonuclease PH (ZMO_RS00070)
RNA Metabolism / ribonuclease E/G (ZMO_RS00830)
RNA Metabolism / ribosome maturation factor RimM (ZMO_RS04815)
RNA Metabolism / 16S rRNA (uracil(1498)-N(3))-methyltransferase (ZMO_RS06955)
RNA Metabolism / ribosomal large subunit pseudouridine synthase D (ZMO_RS03355)
RNA Metabolism / 16S rRNA (guanine(966)-N(2))-methyltransferase RsmD (ZMO_RS02565)
RNA Metabolism / 23S rRNA (pseudouridine(1915)-N(3))-methyltransferase RlmH (ZMO_RS07480)
RNA Metabolism / ribosomal RNA small subunit methyltransferase H (ZMO_RS03675)
RNA Metabolism / FADH(2)-oxidizing methylenetetrahydrofolate--tRNA-(uracil(54)-C(5))- methyltransferase TrmFO (ZMO_RS03080)
RNA Metabolism / rRNA methyltransferase (ZMO_RS05400)
RNA Metabolism / tRNA (adenosine(37)-N6)-threonylcarbamoyltransferase complex dimerization subunit type 1 TsaB (ZMO_RS06330)
RNA Metabolism / tRNA (guanosine(37)-N1)-methyltransferase TrmD (ZMO_RS04820)
RNA Metabolism / tRNA (guanosine(46)-N7)-methyltransferase TrmB (ZMO_RS00625)
RNA Metabolism / tRNA pseudouridine(55) synthase TruB (ZMO_RS02440)
RNA Metabolism / ribonuclease D (ZMO_RS03185)
RNA Metabolism / ribonuclease H (ZMO_RS07185)
RNA Metabolism / polyribonucleotide nucleotidyltransferase (ZMO_RS02430)
RNA Metabolism / LysR family transcriptional regulator (ZMO_RS00210)
RNA Metabolism / YebC/PmpR family DNA-binding transcriptional regulator (ZMO_RS00645)
RNA Metabolism / MerR family DNA-binding transcriptional regulator (ZMO_RS00875)
RNA Metabolism / RNA chaperone Hfq (ZMO_RS01480)
RNA Metabolism / DNA-binding response regulator (ZMO_RS02055)
RNA Metabolism / Lrp/AsnC family transcriptional regulator (ZMO_RS04950)
RNA Metabolism / AraC family transcriptional regulator (ZMO_RS06165)
RNA Metabolism / DNA-binding response regulator (ZMO_RS06195)
RNA Metabolism / transcriptional repressor (ZMO_RS06315)
RNA Metabolism / MucR family transcriptional regulator (ZMO_RS06320)
RNA Metabolism / LysR family transcriptional regulator (ZMO_RS07805)
RNA Metabolism / methyltransferase domain-containing protein (ZMO_RS07860)
RNA Metabolism / LysR family transcriptional regulator (ZMO_RS08065)
RNA Metabolism / integration host factor subunit beta (ZMO_RS08100)
RNA Metabolism / PLP-dependent aminotransferase family protein (ZMO_RS08340)
RNA Metabolism / PLP-dependent aminotransferase family protein (ZMO_RS08855)
RNA Metabolism / glutamate uptake regulatory protein (ZMO_RS01970)
RNA Metabolism / phosphate regulon transcriptional regulatory protein PhoB (ZMO_RS05215)
RNA Metabolism / LysR family transcriptional regulator (ZMO_RS05980)
RNA Metabolism / hypothetical protein (ZMO_RS08835)
RNA Metabolism / RNA polymerase sigma factor RpoD (ZMO_RS07275)
RNA Metabolism / RNA polymerase sigma factor RpoH (ZMO_RS03350)
RNA Metabolism / RNA polymerase sigma-54 factor (ZMO_RS01195)
RNA Metabolism / HrcA family transcriptional regulator (ZMO_RS00075)
RNA Metabolism / transcriptional regulator NrdR (ZMO_RS05395)
RNA Metabolism / transcription elongation factor GreB (ZMO_RS03200)
RNA Metabolism / transcription elongation factor GreA (ZMO_RS07250)
RNA Metabolism / transcription termination/antitermination factor NusG (ZMO_RS03235)
RNA Metabolism / elongation factor 3 (ZMO_RS00465)
RNA Metabolism / cysteine--tRNA ligase (ZMO_RS00790)
RNA Metabolism / 23S rRNA (guanosine(2251)-2'-O)-methyltransferase RlmB (ZMO_RS01000)
RNA Metabolism / proline--tRNA ligase (ZMO_RS01985)
RNA Metabolism / DNA-directed RNA polymerase subunit alpha (ZMO_RS02380)
RNA Metabolism / RNA-binding protein S4 (ZMO_RS02570)
RNA Metabolism / DNA-directed RNA polymerase subunit beta' (ZMO_RS03270)
RNA Metabolism / threonine--tRNA ligase (ZMO_RS03415)
RNA Metabolism / alanine--tRNA ligase (ZMO_RS03775)
RNA Metabolism / glutamate--tRNA ligase 1 (ZMO_RS04015)
RNA Metabolism / RNA methyltransferase (ZMO_RS05955)
RNA Metabolism / leucine--tRNA ligase (ZMO_RS06425)
RNA Metabolism / glycine--tRNA ligase subunit beta (ZMO_RS06470)
[illegible]

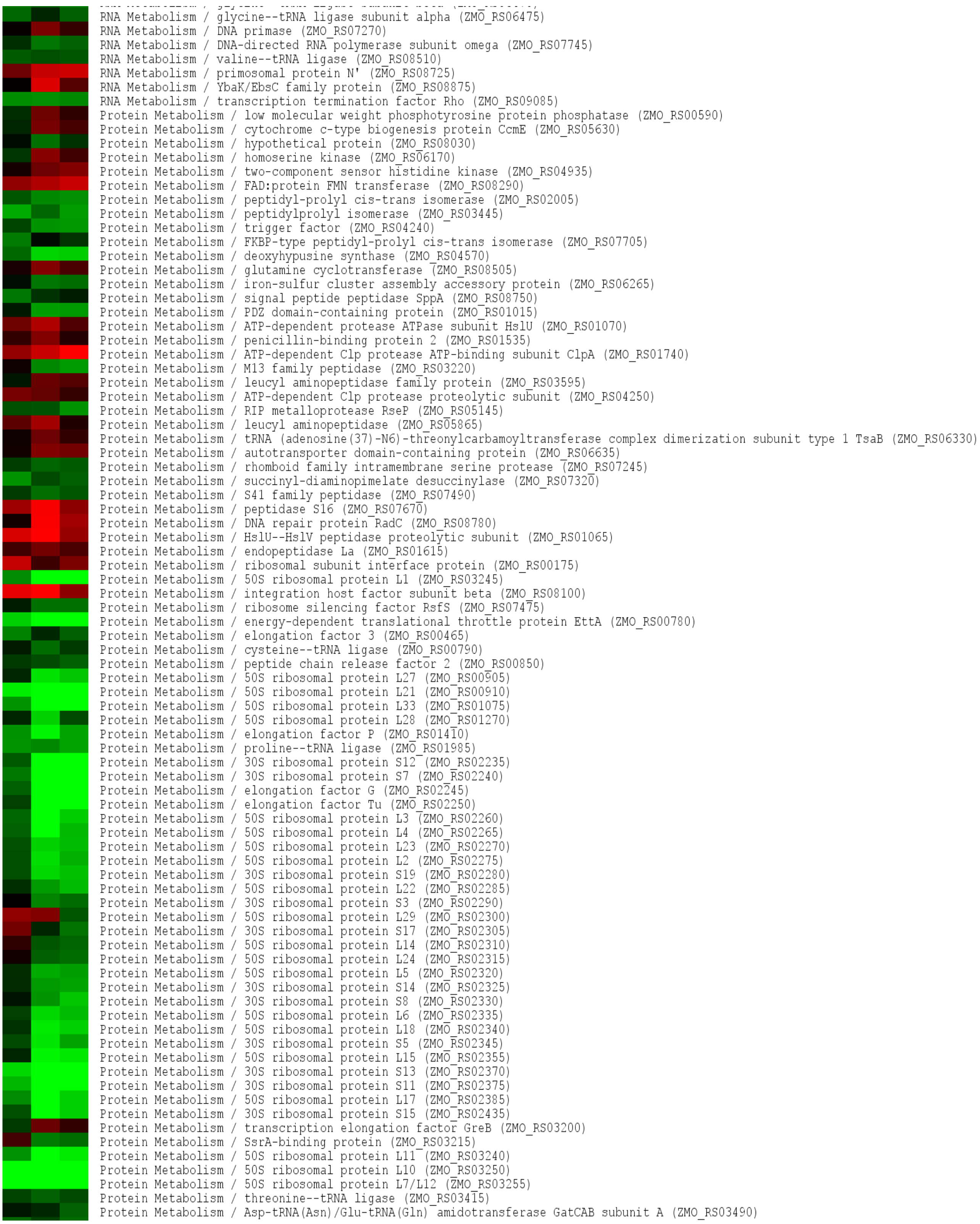
RNA Metabolism / glycine--tRNA ligase subunit alpha (ZMO_RS06475)
RNA Metabolism / DNA primase (ZMO_RS07270)
RNA Metabolism / DNA-directed RNA polymerase subunit omega (ZMO_RS07745)
RNA Metabolism / valine--tRNA ligase (ZMO_RS08510)
RNA Metabolism / primosomal protein N' (ZMO_RS08725)
RNA Metabolism / YbaK/EbsC family protein (ZMO_RS08875)
RNA Metabolism / transcription termination factor Rho (ZMO_RS09085)
Protein Metabolism / low molecular weight phosphotyrosine protein phosphatase (ZMO_RS00590)
Protein Metabolism / cytochrome c-type biogenesis protein CcmE (ZMO_RS05630)
Protein Metabolism / hypothetical protein (ZMO_RS08030)
Protein Metabolism / homoserine kinase (ZMO_RS06170)
Protein Metabolism / two-component sensor histidine kinase (ZMO_RS04935)
Protein Metabolism / FAD:protein FMN transferase (ZMO_RS08290)
Protein Metabolism / peptidyl-prolyl cis-trans isomerase (ZMO_RS02005)
Protein Metabolism / peptidylprolyl isomerase (ZMO_RS03445)
Protein Metabolism / trigger factor (ZMO_RS04240)
Protein Metabolism / FKBP-type peptidyl-prolyl cis-trans isomerase (ZMO_RS07705)
Protein Metabolism / deoxyhypusine synthase (ZMO_RS04570)
Protein Metabolism / glutamine cyclotransferase (ZMO_RS08505)
Protein Metabolism / iron-sulfur cluster assembly accessory protein (ZMO_RS06265)
Protein Metabolism / signal peptide peptidase SppA (ZMO_RS08750)
Protein Metabolism / PDZ domain-containing protein (ZMO_RS01015)
Protein Metabolism / ATP-dependent protease ATPase subunit HslU (ZMO_RS01070)
Protein Metabolism / penicillin-binding protein 2 (ZMO_RS01535)
Protein Metabolism / ATP-dependent Clp protease ATP-binding subunit ClpA (ZMO_RS01740)
Protein Metabolism / M13 family peptidase (ZMO_RS03220)
Protein Metabolism / leucyl aminopeptidase family protein (ZMO_RS03595)
Protein Metabolism / ATP-dependent Clp protease proteolytic subunit (ZMO_RS04250)
Protein Metabolism / RIP metalloprotease RseP (ZMO_RS05145)
Protein Metabolism / leucyl aminopeptidase (ZMO_RS05865)
Protein Metabolism / tRNA (adenosine(37)-N6)-threonylcarbamoyltransferase complex dimerization subunit type 1 TsaB (ZMO_RS06330)
Protein Metabolism / autotransporter domain-containing protein (ZMO_RS06635)
Protein Metabolism / rhomboid family intramembrane serine protease (ZMO_RS07245)
Protein Metabolism / succinyl-diaminopimelate desuccinylase (ZMO_RS07320)
Protein Metabolism / S41 family peptidase (ZMO_RS07490)
Protein Metabolism / peptidase S16 (ZMO_RS07670)
Protein Metabolism / DNA repair protein RadC (ZMO_RS08780)
Protein Metabolism / HslU--HslV peptidase proteolytic subunit (ZMO_RS01065)
Protein Metabolism / endopeptidase La (ZMO_RS01615)
Protein Metabolism / ribosomal subunit interface protein (ZMO_RS00175)
Protein Metabolism / 50S ribosomal protein L1 (ZMO_RS03245)
Protein Metabolism / integration host factor subunit beta (ZMO_RS08100)
Protein Metabolism / ribosome silencing factor RsfS (ZMO_RS07475)
Protein Metabolism / energy-dependent translational throttle protein EttA (ZMO_RS00780)
Protein Metabolism / elongation factor 3 (ZMO_RS00465)
Protein Metabolism / cysteine--tRNA ligase (ZMO_RS00790)
Protein Metabolism / peptide chain release factor 2 (ZMO_RS00850)
Protein Metabolism / 50S ribosomal protein L27 (ZMO_RS00905)
Protein Metabolism / 50S ribosomal protein L21 (ZMO_RS00910)
Protein Metabolism / 50S ribosomal protein L33 (ZMO_RS01075)
Protein Metabolism / 50S ribosomal protein L28 (ZMO_RS01270)
Protein Metabolism / elongation factor P (ZMO_RS01410)
Protein Metabolism / proline--tRNA ligase (ZMO_RS01985)
Protein Metabolism / 30S ribosomal protein S12 (ZMO_RS02235)
Protein Metabolism / 30S ribosomal protein S7 (ZMO_RS02240)
Protein Metabolism / elongation factor G (ZMO_RS02245)
Protein Metabolism / elongation factor Tu (ZMO_RS02250)
Protein Metabolism / 50S ribosomal protein L3 (ZMO_RS02260)
Protein Metabolism / 50S ribosomal protein L4 (ZMO_RS02265)
Protein Metabolism / 50S ribosomal protein L23 (ZMO_RS02270)
Protein Metabolism / 50S ribosomal protein L2 (ZMO_RS02275)
Protein Metabolism / 30S ribosomal protein S19 (ZMO_RS02280)
Protein Metabolism / 50S ribosomal protein L22 (ZMO_RS02285)
Protein Metabolism / 30S ribosomal protein S3 (ZMO_RS02290)
Protein Metabolism / 50S ribosomal protein L29 (ZMO_RS02300)
Protein Metabolism / 30S ribosomal protein S17 (ZMO_RS02305)
Protein Metabolism / 50S ribosomal protein L14 (ZMO_RS02310)
Protein Metabolism / 50S ribosomal protein L24 (ZMO_RS02315)
Protein Metabolism / 50S ribosomal protein L5 (ZMO_RS02320)
Protein Metabolism / 30S ribosomal protein S14 (ZMO_RS02325)
Protein Metabolism / 30S ribosomal protein S8 (ZMO_RS02330)
Protein Metabolism / 50S ribosomal protein L6 (ZMO_RS02335)
Protein Metabolism / 50S ribosomal protein L18 (ZMO_RS02340)
Protein Metabolism / 30S ribosomal protein S5 (ZMO_RS02345)
Protein Metabolism / 50S ribosomal protein L15 (ZMO_RS02355)
Protein Metabolism / 30S ribosomal protein S13 (ZMO_RS02370)
Protein Metabolism / 30S ribosomal protein S11 (ZMO_RS02375)
Protein Metabolism / 50S ribosomal protein L17 (ZMO_RS02385)
Protein Metabolism / 30S ribosomal protein S15 (ZMO_RS02435)
Protein Metabolism / transcription elongation factor GreB (ZMO_RS03200)
Protein Metabolism / SsrA-binding protein (ZMO_RS03215)
Protein Metabolism / 50S ribosomal protein L11 (ZMO_RS03240)
Protein Metabolism / 50S ribosomal protein L10 (ZMO_RS03250)
Protein Metabolism / 50S ribosomal protein L7/L12 (ZMO_RS03255)
Protein Metabolism / threonine--tRNA ligase (ZMO_RS03415)
Protein Metabolism / Asp-tRNA(Asn)/Glu-tRNA(Gln) amidotransferase GatCAB subunit A (ZMO_RS03490)

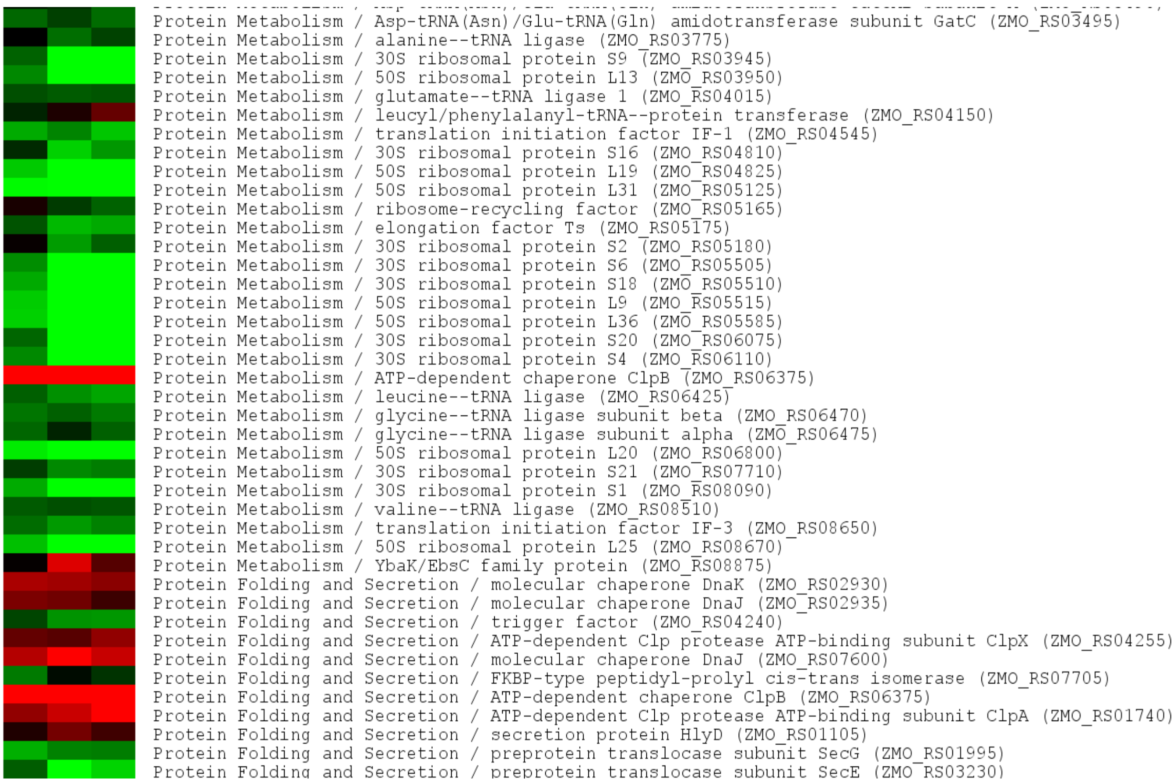
Protein Metabolism / Asp-tRNA(Asn)/Glu-tRNA(Gln) amidotransferase subunit GatC (ZMO_RS03495)
Protein Metabolism / alanine--tRNA ligase (ZMO_RS03775)
Protein Metabolism / 30S ribosomal protein S9 (ZMO_RS03945)
Protein Metabolism / 50S ribosomal protein L13 (ZMO_RS03950)
Protein Metabolism / glutamate--tRNA ligase 1 (ZMO_RS04015)
Protein Metabolism / leucyl/phenylalanyl-tRNA--protein transferase (ZMO_RS04150)
Protein Metabolism / translation initiation factor IF-1 (ZMO_RS04545)
Protein Metabolism / 30S ribosomal protein S16 (ZMO_RS04810)
Protein Metabolism / 50S ribosomal protein L19 (ZMO_RS04825)
Protein Metabolism / 50S ribosomal protein L31 (ZMO_RS05125)
Protein Metabolism / ribosome-recycling factor (ZMO_RS05165)
Protein Metabolism / elongation factor Ts (ZMO_RS05175)
Protein Metabolism / 30S ribosomal protein S2 (ZMO_RS05180)
Protein Metabolism / 30S ribosomal protein S6 (ZMO_RS05505)
Protein Metabolism / 30S ribosomal protein S18 (ZMO_RS05510)
Protein Metabolism / 50S ribosomal protein L9 (ZMO_RS05515)
Protein Metabolism / 50S ribosomal protein L36 (ZMO_RS05585)
Protein Metabolism / 30S ribosomal protein S20 (ZMO_RS06075)
Protein Metabolism / 30S ribosomal protein S4 (ZMO_RS06110)
Protein Metabolism / ATP-dependent chaperone ClpB (ZMO_RS06375)
Protein Metabolism / leucine--tRNA ligase (ZMO_RS06425)
Protein Metabolism / glycine--tRNA ligase subunit beta (ZMO_RS06470)
Protein Metabolism / glycine--tRNA ligase subunit alpha (ZMO_RS06475)
Protein Metabolism / 50S ribosomal protein L20 (ZMO_RS06800)
Protein Metabolism / 30S ribosomal protein S21 (ZMO_RS07710)
Protein Metabolism / 30S ribosomal protein S1 (ZMO_RS08090)
Protein Metabolism / valine--tRNA ligase (ZMO_RS08510)
Protein Metabolism / translation initiation factor IF-3 (ZMO_RS08650)
Protein Metabolism / 50S ribosomal protein L25 (ZMO_RS08670)
Protein Metabolism / YbaK/EbsC family protein (ZMO_RS08875)
Protein Folding and Secretion / molecular chaperone DnaK (ZMO_RS02930)
Protein Folding and Secretion / molecular chaperone DnaJ (ZMO_RS02935)
Protein Folding and Secretion / trigger factor (ZMO_RS04240)
Protein Folding and Secretion / ATP-dependent Clp protease ATP-binding subunit ClpX (ZMO_RS04255)
Protein Folding and Secretion / molecular chaperone DnaJ (ZMO_RS07600)
Protein Folding and Secretion / FKBP-type peptidyl-prolyl cis-trans isomerase (ZMO_RS07705)
Protein Folding and Secretion / ATP-dependent chaperone ClpB (ZMO_RS06375)
Protein Folding and Secretion / ATP-dependent Clp protease ATP-binding subunit ClpA (ZMO_RS01740)
Protein Folding and Secretion / secretion protein HlyD (ZMO_RS01105)
Protein Folding and Secretion / preprotein translocase subunit SecG (ZMO_RS01995)
Protein Folding and Secretion / preprotein translocase subunit SecE (ZMO_RS03230)

Supplementary Material S3B – Biosynthesis of biomolecules

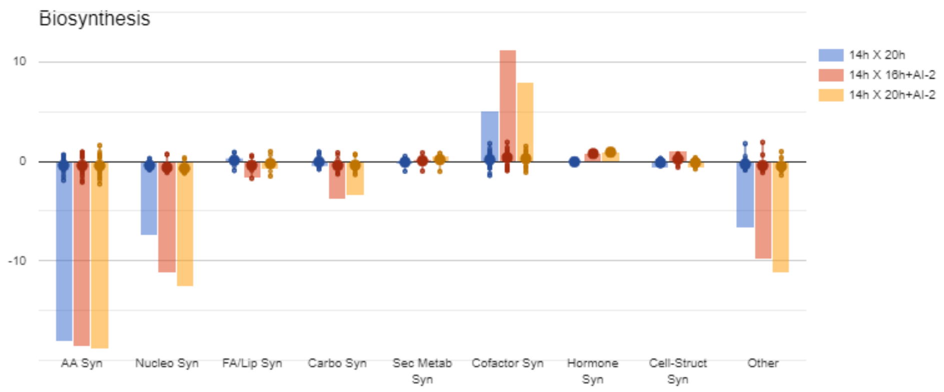

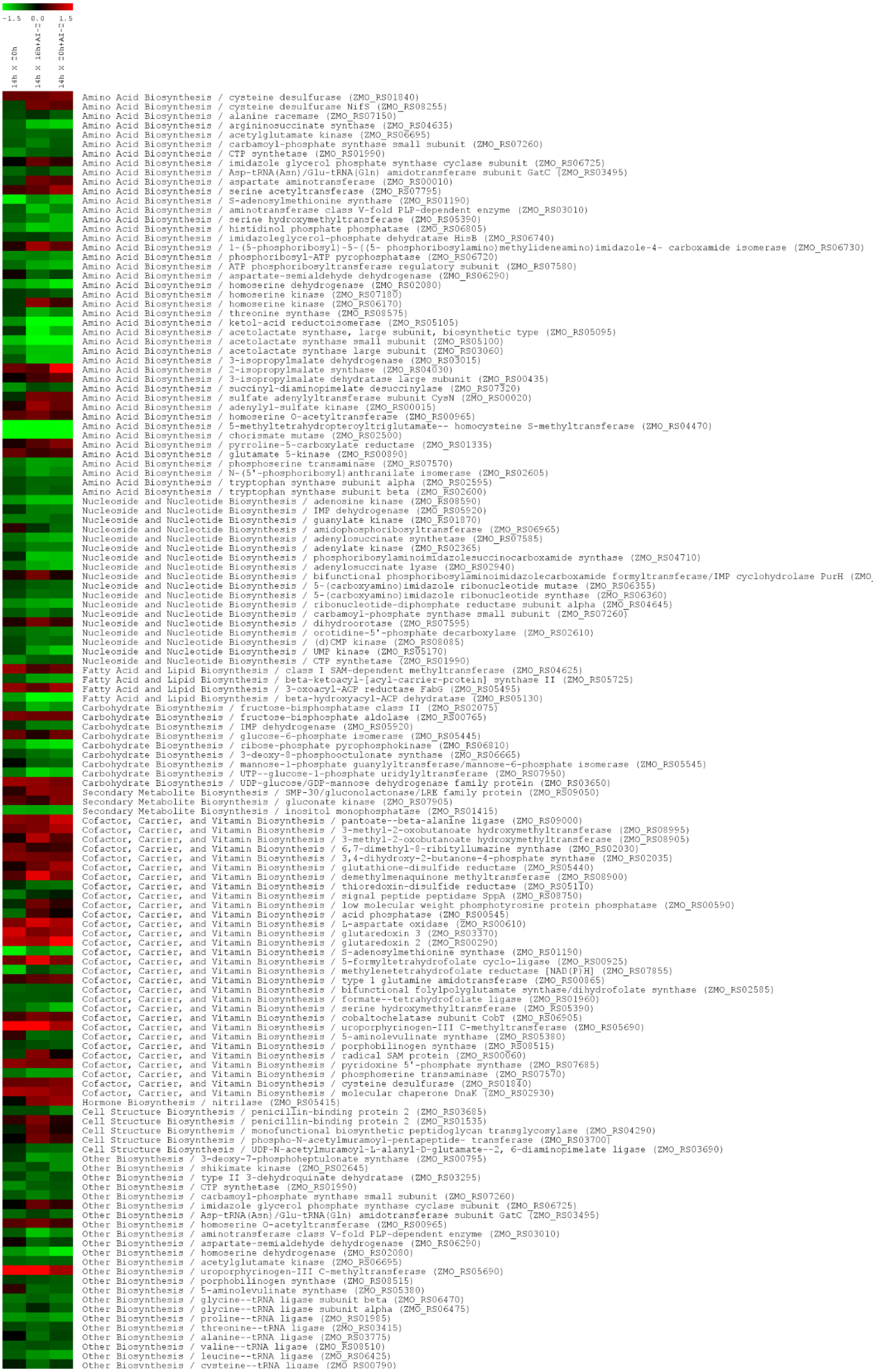
-1.5 0.0 1.5
14h X 20h
14h X 16h+AI-2
14h X 20h+AI-2
Amino Acid Biosynthesis / cysteine desulfurase (ZMO_RS01840)
Amino Acid Biosynthesis / cysteine desulfurase NifS (ZMO_RS08255)
Amino Acid Biosynthesis / alanine racemase (ZMO_RS07150)
Amino Acid Biosynthesis / argininosuccinate synthase (ZMO_RS04635)
Amino Acid Biosynthesis / acetylglutamate kinase (ZMO_RS06695)
Amino Acid Biosynthesis / carbamoyl-phosphate synthase small subunit (ZMO_RS07260)
Amino Acid Biosynthesis / CTP synthetase (ZMO_RS01990)
Amino Acid Biosynthesis / imidazole glycerol phosphate synthase cyclase subunit (ZMO_RS06725)
Amino Acid Biosynthesis / Asp-tRNA(Asn)/Glu-tRNA(Gln) amidotransferase subunit GatC (ZMO_RS03495)
Amino Acid Biosynthesis / aspartate aminotransferase (ZMO_RS00010)
Amino Acid Biosynthesis / serine acetyltransferase (ZMO_RS07795)
Amino Acid Biosynthesis / S-adenosylmethionine synthase (ZMO_RS01190)
Amino Acid Biosynthesis / aminotransferase class V-fold PLP-dependent enzyme (ZMO_RS03010)
Amino Acid Biosynthesis / serine hydroxymethyltransferase (ZMO_RS05390)
Amino Acid Biosynthesis / histidinol phosphate phosphatase (ZMO_RS06805)
Amino Acid Biosynthesis / imidazoleglycerol-phosphate dehydratase HisB (ZMO_RS06740)
Amino Acid Biosynthesis / 1-(5-phosphoribosyl)-5-((5- phosphoribosylamino)methylideneamino)imidazole-4- carboxamide isomerase (ZMO_RS06730)
Amino Acid Biosynthesis / phosphoribosyl-ATP pyrophosphatase (ZMO_RS06720)
Amino Acid Biosynthesis / ATP phosphoribosyltransferase regulatory subunit (ZMO_RS07580)
Amino Acid Biosynthesis / aspartate-semialdehyde dehydrogenase (ZMO_RS06290)
Amino Acid Biosynthesis / homoserine dehydrogenase (ZMO_RS02080)
Amino Acid Biosynthesis / homoserine kinase (ZMO_RS07180)
Amino Acid Biosynthesis / homoserine kinase (ZMO_RS06170)
Amino Acid Biosynthesis / threonine synthase (ZMO_RS08575)
Amino Acid Biosynthesis / ketol-acid reductoisomerase (ZMO_RS05105)
Amino Acid Biosynthesis / acetolactate synthase, large subunit, biosynthetic type (ZMO_RS05095)
Amino Acid Biosynthesis / acetolactate synthase small subunit (ZMO_RS05100)
Amino Acid Biosynthesis / acetolactate synthase large subunit (ZMO_RS03060)
Amino Acid Biosynthesis / 3-isopropylmalate dehydrogenase (ZMO_RS03015)
Amino Acid Biosynthesis / 2-isopropylmalate synthase (ZMO_RS04030)
Amino Acid Biosynthesis / 3-isopropylmalate dehydratase large subunit (ZMO_RS00435)
Amino Acid Biosynthesis / succinyl-diaminopimelate desuccinylase (ZMO_RS07320)
Amino Acid Biosynthesis / sulfate adenylyltransferase subunit CysN (ZMO_RS00020)
Amino Acid Biosynthesis / adenylyl-sulfate kinase (ZMO_RS00015)
Amino Acid Biosynthesis / homoserine O-acetyltransferase (ZMO_RS00965)
Amino Acid Biosynthesis / 5-methyltetrahydropteroyltriglutamate-- homocysteine S-methyltransferase (ZMO_RS04470)
Amino Acid Biosynthesis / chorismate mutase (ZMO_RS02500)
Amino Acid Biosynthesis / pyrroline-5-carboxylate reductase (ZMO_RS01335)
Amino Acid Biosynthesis / glutamate 5-kinase (ZMO_RS00890)
Amino Acid Biosynthesis / phosphoserine transaminase (ZMO_RS07570)
Amino Acid Biosynthesis / N-(5'-phosphoribosyl)anthranilate isomerase (ZMO_RS02605)
Amino Acid Biosynthesis / tryptophan synthase subunit alpha (ZMO_RS02595)
Amino Acid Biosynthesis / tryptophan synthase subunit beta (ZMO_RS02600)
Nucleoside and Nucleotide Biosynthesis / adenosine kinase (ZMO_RS08590)
Nucleoside and Nucleotide Biosynthesis / IMP dehydrogenase (ZMO_RS05920)
Nucleoside and Nucleotide Biosynthesis / guanylate kinase (ZMO_RS01870)
Nucleoside and Nucleotide Biosynthesis / amidophosphoribosyltransferase (ZMO_RS06965)
Nucleoside and Nucleotide Biosynthesis / adenylosuccinate synthetase (ZMO_RS07585)
Nucleoside and Nucleotide Biosynthesis / adenylate kinase (ZMO_RS02365)
Nucleoside and Nucleotide Biosynthesis / phosphoribosylaminoimidazolesuccinocarboxamide synthase (ZMO_RS04710)
Nucleoside and Nucleotide Biosynthesis / adenylosuccinate lyase (ZMO_RS02940)
Nucleoside and Nucleotide Biosynthesis / bifunctional phosphoribosylaminoimidazolecarboxamide formyltransferase/IMP cyclohydrolase PurH (ZMO_RS00130)
Nucleoside and Nucleotide Biosynthesis / 5-(carboxyamino)imidazole ribonucleotide mutase (ZMO_RS06355)
Nucleoside and Nucleotide Biosynthesis / 5-(carboxyamino)imidazole ribonucleotide synthase (ZMO_RS06360)
Nucleoside and Nucleotide Biosynthesis / ribonucleotide-diphosphate reductase subunit alpha (ZMO_RS04645)
Nucleoside and Nucleotide Biosynthesis / carbamoyl-phosphate synthase small subunit (ZMO_RS07260)
Nucleoside and Nucleotide Biosynthesis / dihydroorotase (ZMO_RS07595)
Nucleoside and Nucleotide Biosynthesis / orotidine-5'-phosphate decarboxylase (ZMO_RS02610)
Nucleoside and Nucleotide Biosynthesis / (d)CMP kinase (ZMO_RS08085)
Nucleoside and Nucleotide Biosynthesis / UMP kinase (ZMO_RS05170)
Nucleoside and Nucleotide Biosynthesis / CTP synthetase (ZMO_RS01990)
Fatty Acid and Lipid Biosynthesis / class I SAM-dependent methyltransferase (ZMO_RS04625)
Fatty Acid and Lipid Biosynthesis / beta-ketoacyl-[acyl-carrier-protein] synthase II (ZMO_RS05725)
Fatty Acid and Lipid Biosynthesis / 3-oxoacyl-ACP reductase FabG (ZMO_RS05495)
Fatty Acid and Lipid Biosynthesis / beta-hydroxyacyl-ACP dehydratase (ZMO_RS05130)
Carbohydrate Biosynthesis / fructose-bisphosphatase class II (ZMO_RS02075)
Carbohydrate Biosynthesis / fructose-bisphosphate aldolase (ZMO_RS00765)
Carbohydrate Biosynthesis / IMP dehydrogenase (ZMO_RS05920)
Carbohydrate Biosynthesis / glucose-6-phosphate isomerase (ZMO_RS05445)
Carbohydrate Biosynthesis / ribose-phosphate pyrophosphokinase (ZMO_RS06810)
Carbohydrate Biosynthesis / 3-deoxy-8-phosphooctulonate synthase (ZMO_RS06665)
Carbohydrate Biosynthesis / mannose-1-phosphate guanylyltransferase/mannose-6-phosphate isomerase (ZMO_RS05545)
Carbohydrate Biosynthesis / UTP--glucose-1-phosphate uridylyltransferase (ZMO_RS07950)
Carbohydrate Biosynthesis / UDP-glucose/GDP-mannose dehydrogenase family protein (ZMO_RS03650)
Secondary Metabolite Biosynthesis / SMP-30/gluconolactonase/LRE family protein (ZMO_RS09050)
Secondary Metabolite Biosynthesis / gluconate kinase (ZMO_RS07905)
Secondary Metabolite Biosynthesis / inositol monophosphatase (ZMO_RS01415)
Cofactor, Carrier, and Vitamin Biosynthesis / pantoate--beta-alanine ligase (ZMO_RS09000)
Cofactor, Carrier, and Vitamin Biosynthesis / 3-methyl-2-oxobutanoate hydroxymethyltransferase (ZMO_RS08995)
Cofactor, Carrier, and Vitamin Biosynthesis / 3-methyl-2-oxobutanoate hydroxymethyltransferase (ZMO_RS08905)
Cofactor, Carrier, and Vitamin Biosynthesis / 6,7-dimethyl-8-ribityllumazine synthase (ZMO_RS02030)
Cofactor, Carrier, and Vitamin Biosynthesis / 3,4-dihydroxy-2-butanone-4-phosphate synthase (ZMO_RS02035)
Cofactor, Carrier, and Vitamin Biosynthesis / glutathione-disulfide reductase (ZMO_RS05440)
Cofactor, Carrier, and Vitamin Biosynthesis / demethylmenaquinone methyltransferase (ZMO_RS08900)
Cofactor, Carrier, and Vitamin Biosynthesis / thioredoxin-disulfide reductase (ZMO_RS05110)
Cofactor, Carrier, and Vitamin Biosynthesis / signal peptide peptidase SppA (ZMO_RS08750)
Cofactor, Carrier, and Vitamin Biosynthesis / low molecular weight phosphotyrosine protein phosphatase (ZMO_RS00590)
Cofactor, Carrier, and Vitamin Biosynthesis / acid phosphatase (ZMO_RS00545)
Cofactor, Carrier, and Vitamin Biosynthesis / L-aspartate oxidase (ZMO_RS00610)
Cofactor, Carrier, and Vitamin Biosynthesis / glutaredoxin 3 (ZMO_RS03370)
Cofactor, Carrier, and Vitamin Biosynthesis / glutaredoxin 2 (ZMO_RS00290)
Cofactor, Carrier, and Vitamin Biosynthesis / S-adenosylmethionine synthase (ZMO_RS01190)
Cofactor, Carrier, and Vitamin Biosynthesis / 5-formyltetrahydrofolate cyclo-ligase (ZMO_RS00925)
Cofactor, Carrier, and Vitamin Biosynthesis / methylenetetrahydrofolate reductase [NAD(P)H] (ZMO_RS07855)
Cofactor, Carrier, and Vitamin Biosynthesis / type 1 glutamine amidotransferase (ZMO_RS00865)
Cofactor, Carrier, and Vitamin Biosynthesis / bifunctional folylpolyglutamate synthase/dihydrofolate synthase (ZMO_RS02585)
Cofactor, Carrier, and Vitamin Biosynthesis / formate--tetrahydrofolate ligase (ZMO_RS01960)
Cofactor, Carrier, and Vitamin Biosynthesis / serine hydroxymethyltransferase (ZMO_RS05390)
Cofactor, Carrier, and Vitamin Biosynthesis / cobaltochelatase subunit CobT (ZMO_RS06905)
Cofactor, Carrier, and Vitamin Biosynthesis / uroporphyrinogen-III C-methyltransferase (ZMO_RS05690)
Cofactor, Carrier, and Vitamin Biosynthesis / 5-aminolevulinate synthase (ZMO_RS05380)
Cofactor, Carrier, and Vitamin Biosynthesis / porphobilinogen synthase (ZMO_RS08515)
Cofactor, Carrier, and Vitamin Biosynthesis / radical SAM protein (ZMO_RS00060)
Cofactor, Carrier, and Vitamin Biosynthesis / pyridoxine 5'-phosphate synthase (ZMO_RS07685)
Cofactor, Carrier, and Vitamin Biosynthesis / phosphoserine transaminase (ZMO_RS07570)
Cofactor, Carrier, and Vitamin Biosynthesis / cysteine desulfurase (ZMO_RS01840)
Cofactor, Carrier, and Vitamin Biosynthesis / molecular chaperone DnaK (ZMO_RS02930)
Hormone Biosynthesis / nitrilase (ZMO_RS05415)
Cell Structure Biosynthesis / penicillin-binding protein 2 (ZMO_RS03685)
Cell Structure Biosynthesis / penicillin-binding protein 2 (ZMO_RS01535)
Cell Structure Biosynthesis / monofunctional biosynthetic peptidoglycan transglycosylase (ZMO_RS04290)
Cell Structure Biosynthesis / phospho-N-acetylmuramoyl-pentapeptide- transferase (ZMO_RS03700)
Cell Structure Biosynthesis / UDP-N-acetylmuramoyl-L-alanyl-D-glutamate--2, 6-diaminopimelate ligase (ZMO_RS03690)
Other Biosynthesis / 3-deoxy-7-phosphoheptulonate synthase (ZMO_RS00795)
Other Biosynthesis / shikimate kinase (ZMO_RS02645)
Other Biosynthesis / type II 3-dehydroquinate dehydratase (ZMO_RS03295)
Other Biosynthesis / CTP synthetase (ZMO_RS01990)
Other Biosynthesis / carbamoyl-phosphate synthase small subunit (ZMO_RS07260)
Other Biosynthesis / imidazole glycerol phosphate synthase cyclase subunit (ZMO_RS06725)
Other Biosynthesis / Asp-tRNA(Asn)/Glu-tRNA(Gln) amidotransferase subunit GatC (ZMO_RS03495)
Other Biosynthesis / homoserine O-acetyltransferase (ZMO_RS00965)
Other Biosynthesis / aminotransferase class V-fold PLP-dependent enzyme (ZMO_RS03010)
Other Biosynthesis / aspartate-semialdehyde dehydrogenase (ZMO_RS06290)
Other Biosynthesis / homoserine dehydrogenase (ZMO_RS02080)
Other Biosynthesis / acetylglutamate kinase (ZMO_RS06695)
Other Biosynthesis / uroporphyrinogen-III C-methyltransferase (ZMO_RS05690)
Other Biosynthesis / porphobilinogen synthase (ZMO_RS08515)
Other Biosynthesis / 5-aminolevulinate synthase (ZMO_RS05380)
Other Biosynthesis / glycine--tRNA ligase subunit beta (ZMO_RS06470)
Other Biosynthesis / glycine--tRNA ligase subunit alpha (ZMO_RS06475)
Other Biosynthesis / proline--tRNA ligase (ZMO_RS01985)
Other Biosynthesis / threonine--tRNA ligase (ZMO_RS03415)
Other Biosynthesis / alanine--tRNA ligase (ZMO_RS03775)
Other Biosynthesis / valine--tRNA ligase (ZMO_RS08510)
Other Biosynthesis / leucine--tRNA ligase (ZMO_RS06425)
Other Biosynthesis / cysteine--tRNA ligase (ZMO_RS00790)

Supplementary Material S3C – Degradation of biomolecules

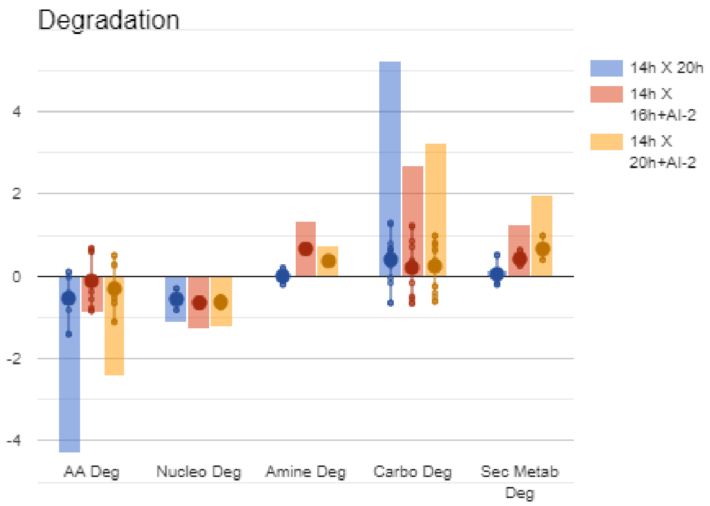

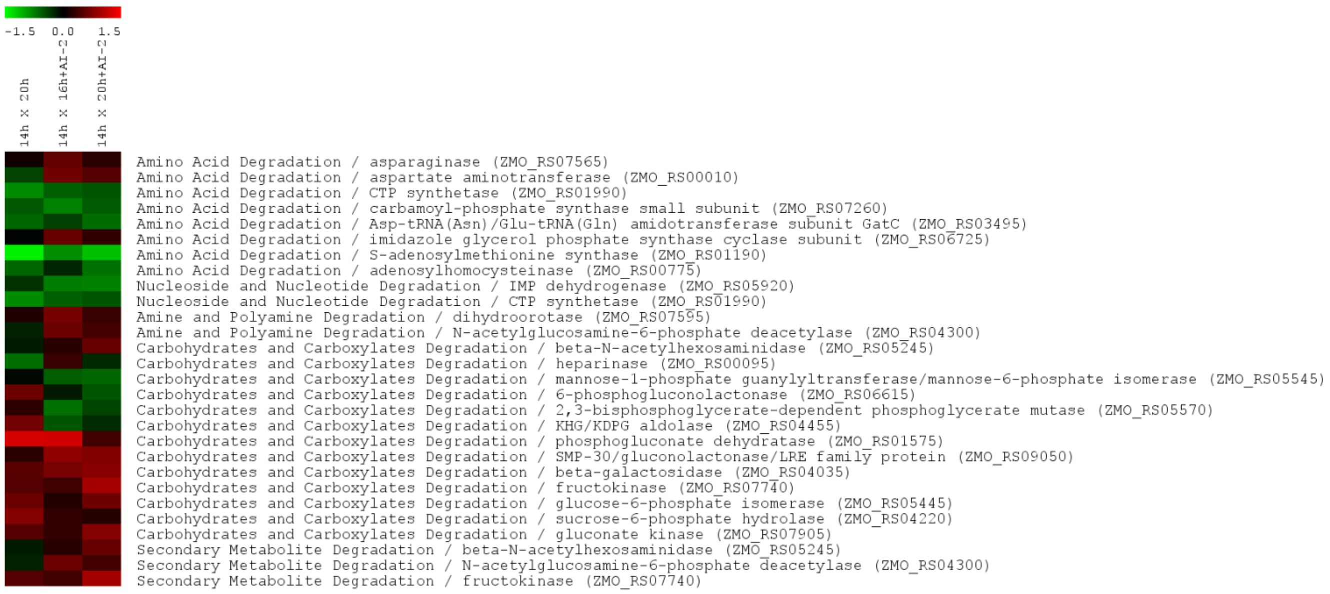

Supplementary Material S3D – Cell Exterior Structures

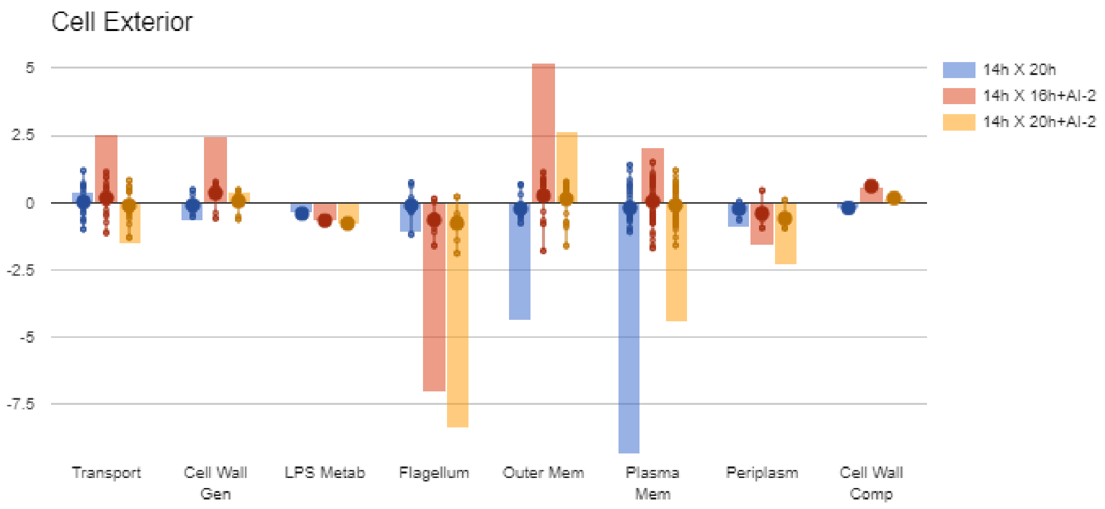

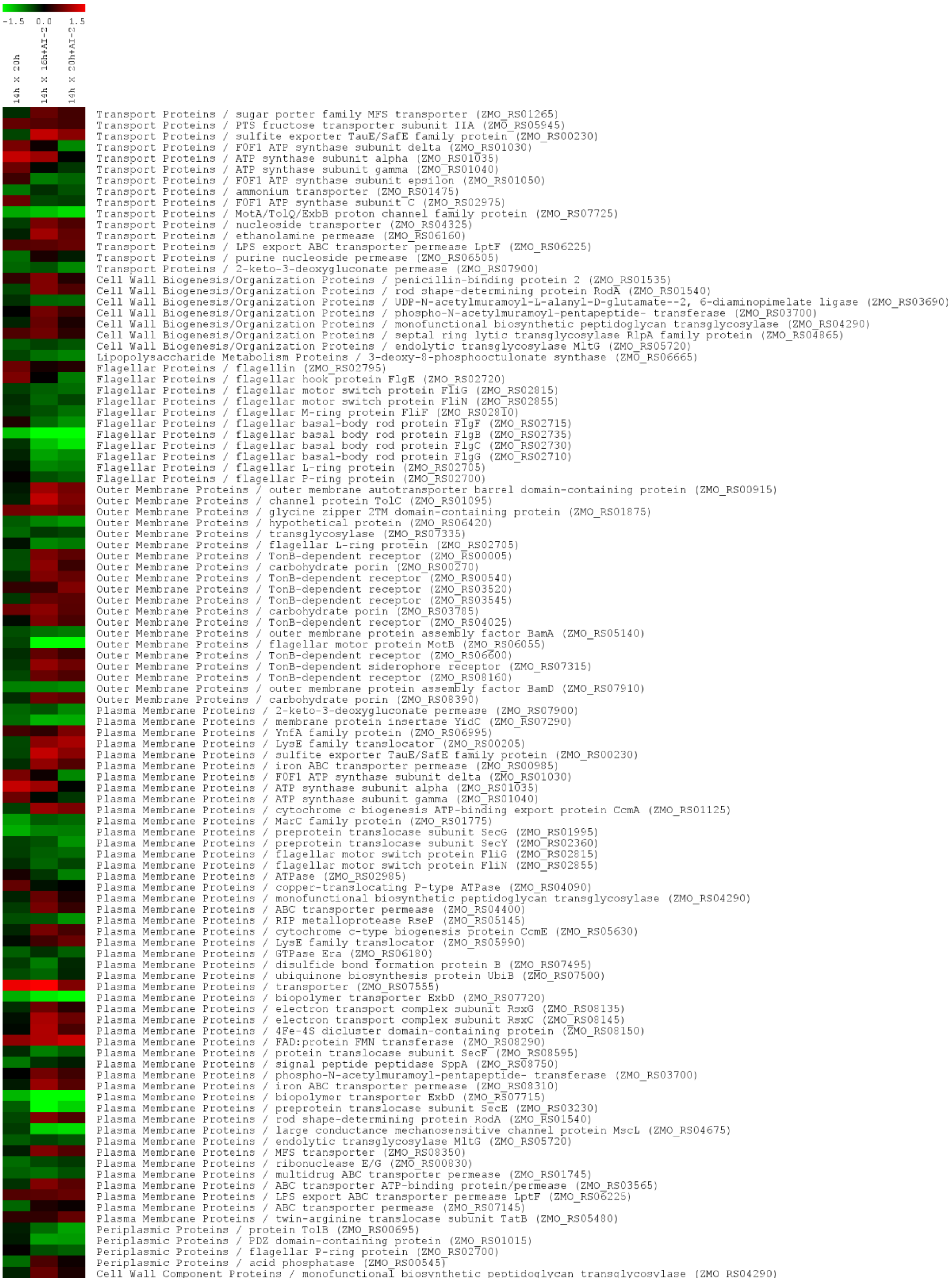

-1.5 0.0 1.5
14h X 20h
14h X 16h+AI-2
14h X 20h+AI-2
Transport Proteins / sugar porter family MFS transporter (ZMO_RS01265)
Transport Proteins / PTS fructose transporter subunit IIA (ZMO_RS05945)
Transport Proteins / sulfite exporter TauE/SafE family protein (ZMO_RS00230)
Transport Proteins / F0F1 ATP synthase subunit delta (ZMO_RS01030)
Transport Proteins / ATP synthase subunit alpha (ZMO_RS01035)
Transport Proteins / ATP synthase subunit gamma (ZMO_RS01040)
Transport Proteins / F0F1 ATP synthase subunit epsilon (ZMO_RS01050)
Transport Proteins / ammonium transporter (ZMO_RS01475)
Transport Proteins / F0F1 ATP synthase subunit C (ZMO_RS02975)
Transport Proteins / MotA/TolQ/ExbB proton channel family protein (ZMO_RS07725)
Transport Proteins / nucleoside transporter (ZMO_RS04325)
Transport Proteins / ethanolamine permease (ZMO_RS06160)
Transport Proteins / LPS export ABC transporter permease LptF (ZMO_RS06225)
Transport Proteins / purine nucleoside permease (ZMO_RS06505)
Transport Proteins / 2-keto-3-deoxygluconate permease (ZMO_RS07900)
Cell Wall Biogenesis/Organization Proteins / penicillin-binding protein 2 (ZMO_RS01535)
Cell Wall Biogenesis/Organization Proteins / rod shape-determining protein RodA (ZMO_RS01540)
Cell Wall Biogenesis/Organization Proteins / UDP-N-acetylmuramoyl-L-alanyl-D-glutamate--2, 6-diaminopimelate ligase (ZMO_RS03690)
Cell Wall Biogenesis/Organization Proteins / phospho-N-acetylmuramoyl-pentapeptide- transferase (ZMO_RS03700)
Cell Wall Biogenesis/Organization Proteins / monofunctional biosynthetic peptidoglycan transglycosylase (ZMO_RS04290)
Cell Wall Biogenesis/Organization Proteins / septal ring lytic transglycosylase RlpA family protein (ZMO_RS04865)
Cell Wall Biogenesis/Organization Proteins / endolytic transglycosylase MltG (ZMO_RS05720)
Lipopolysaccharide Metabolism Proteins / 3-deoxy-8-phosphooctulonate synthase (ZMO_RS06665)
Flagellar Proteins / flagellin (ZMO_RS02795)
Flagellar Proteins / flagellar hook protein FlgE (ZMO_RS02720)
Flagellar Proteins / flagellar motor switch protein FliG (ZMO_RS02815)
Flagellar Proteins / flagellar motor switch protein FliN (ZMO_RS02855)
Flagellar Proteins / flagellar M-ring protein FliF (ZMO_RS02810)
Flagellar Proteins / flagellar basal-body rod protein FlgF (ZMO_RS02715)
Flagellar Proteins / flagellar basal body rod protein FlgB (ZMO_RS02735)
Flagellar Proteins / flagellar basal body rod protein FlgC (ZMO_RS02730)
Flagellar Proteins / flagellar basal-body rod protein FlgG (ZMO_RS02710)
Flagellar Proteins / flagellar L-ring protein (ZMO_RS02705)
Flagellar Proteins / flagellar P-ring protein (ZMO_RS02700)
Outer Membrane Proteins / outer membrane autotransporter barrel domain-containing protein (ZMO_RS00915)
Outer Membrane Proteins / channel protein TolC (ZMO_RS01095)
Outer Membrane Proteins / glycine zipper 2TM domain-containing protein (ZMO_RS01875)
Outer Membrane Proteins / hypothetical protein (ZMO_RS06420)
Outer Membrane Proteins / transglycosylase (ZMO_RS07335)
Outer Membrane Proteins / flagellar L-ring protein (ZMO_RS02705)
Outer Membrane Proteins / TonB-dependent receptor (ZMO_RS00005)
Outer Membrane Proteins / carbohydrate porin (ZMO_RS00270)
Outer Membrane Proteins / TonB-dependent receptor (ZMO_RS00540)
Outer Membrane Proteins / TonB-dependent receptor (ZMO_RS03520)
Outer Membrane Proteins / TonB-dependent receptor (ZMO_RS03545)
Outer Membrane Proteins / carbohydrate porin (ZMO_RS03785)
Outer Membrane Proteins / TonB-dependent receptor (ZMO_RS04025)
Outer Membrane Proteins / outer membrane protein assembly factor BamA (ZMO_RS05140)
Outer Membrane Proteins / flagellar motor protein MotB (ZMO_RS06055)
Outer Membrane Proteins / TonB-dependent receptor (ZMO_RS06600)
Outer Membrane Proteins / TonB-dependent siderophore receptor (ZMO_RS07315)
Outer Membrane Proteins / TonB-dependent receptor (ZMO_RS08160)
Outer Membrane Proteins / outer membrane protein assembly factor BamD (ZMO_RS07910)
Outer Membrane Proteins / carbohydrate porin (ZMO_RS08390)
Plasma Membrane Proteins / 2-keto-3-deoxygluconate permease (ZMO_RS07900)
Plasma Membrane Proteins / membrane protein insertase YidC (ZMO_RS07290)
Plasma Membrane Proteins / YnfA family protein (ZMO_RS06995)
Plasma Membrane Proteins / LysE family translocator (ZMO_RS00205)
Plasma Membrane Proteins / sulfite exporter TauE/SafE family protein (ZMO_RS00230)
Plasma Membrane Proteins / iron ABC transporter permease (ZMO_RS00985)
Plasma Membrane Proteins / F0F1 ATP synthase subunit delta (ZMO_RS01030)
Plasma Membrane Proteins / ATP synthase subunit alpha (ZMO_RS01035)
Plasma Membrane Proteins / ATP synthase subunit gamma (ZMO_RS01040)
Plasma Membrane Proteins / cytochrome c biogenesis ATP-binding export protein CcmA (ZMO_RS01125)
Plasma Membrane Proteins / MarC family protein (ZMO_RS01775)
Plasma Membrane Proteins / preprotein translocase subunit SecG (ZMO_RS01995)
Plasma Membrane Proteins / preprotein translocase subunit SecY (ZMO_RS02360)
Plasma Membrane Proteins / flagellar motor switch protein FliG (ZMO_RS02815)
Plasma Membrane Proteins / flagellar motor switch protein FliN (ZMO_RS02855)
Plasma Membrane Proteins / ATPase (ZMO_RS02985)
Plasma Membrane Proteins / copper-translocating P-type ATPase (ZMO_RS04090)
Plasma Membrane Proteins / monofunctional biosynthetic peptidoglycan transglycosylase (ZMO_RS04290)
Plasma Membrane Proteins / ABC transporter permease (ZMO_RS04400)
Plasma Membrane Proteins / RIP metalloprotease RseP (ZMO_RS05145)
Plasma Membrane Proteins / cytochrome c-type biogenesis protein CcmE (ZMO_RS05630)
Plasma Membrane Proteins / LysE family translocator (ZMO_RS05990)
Plasma Membrane Proteins / GTPase Era (ZMO_RS06180)
Plasma Membrane Proteins / disulfide bond formation protein B (ZMO_RS07495)
Plasma Membrane Proteins / ubiquinone biosynthesis protein UbiB (ZMO_RS07500)
Plasma Membrane Proteins / transporter (ZMO_RS07555)
Plasma Membrane Proteins / biopolymer transporter ExbD (ZMO_RS07720)
Plasma Membrane Proteins / electron transport complex subunit RsxG (ZMO_RS08135)
Plasma Membrane Proteins / electron transport complex subunit RsxC (ZMO_RS08145)
Plasma Membrane Proteins / 4Fe-4S dicluster domain-containing protein (ZMO_RS08150)
Plasma Membrane Proteins / FAD:protein FMN transferase (ZMO_RS08290)
Plasma Membrane Proteins / protein translocase subunit SecF (ZMO_RS08595)
Plasma Membrane Proteins / signal peptide peptidase SppA (ZMO_RS08750)
Plasma Membrane Proteins / phospho-N-acetylmuramoyl-pentapeptide- transferase (ZMO_RS03700)
Plasma Membrane Proteins / iron ABC transporter permease (ZMO_RS08310)
Plasma Membrane Proteins / biopolymer transporter ExbD (ZMO_RS07715)
Plasma Membrane Proteins / preprotein translocase subunit SecE (ZMO_RS03230)
Plasma Membrane Proteins / rod shape-determining protein RodA (ZMO_RS01540)
Plasma Membrane Proteins / large conductance mechanosensitive channel protein MscL (ZMO_RS04675)
Plasma Membrane Proteins / endolytic transglycosylase MltG (ZMO_RS05720)
Plasma Membrane Proteins / MFS transporter (ZMO_RS08350)
Plasma Membrane Proteins / ribonuclease E/G (ZMO_RS00830)
Plasma Membrane Proteins / multidrug ABC transporter permease (ZMO_RS01745)
Plasma Membrane Proteins / ABC transporter ATP-binding protein/permease (ZMO_RS03565)
Plasma Membrane Proteins / LPS export ABC transporter permease LptF (ZMO_RS06225)
Plasma Membrane Proteins / ABC transporter permease (ZMO_RS07145)
Plasma Membrane Proteins / twin-arginine translocase subunit TatB (ZMO_RS05480)
Periplasmic Proteins / protein TolB (ZMO_RS00695)
Periplasmic Proteins / PDZ domain-containing protein (ZMO_RS01015)
Periplasmic Proteins / flagellar P-ring protein (ZMO_RS02700)
Periplasmic Proteins / acid phosphatase (ZMO_RS00545)
Cell Wall Component Proteins / monofunctional biosynthetic peptidoglycan transglycosylase (ZMO_RS04290)

Supplementary Material S3E – Cellular Processes

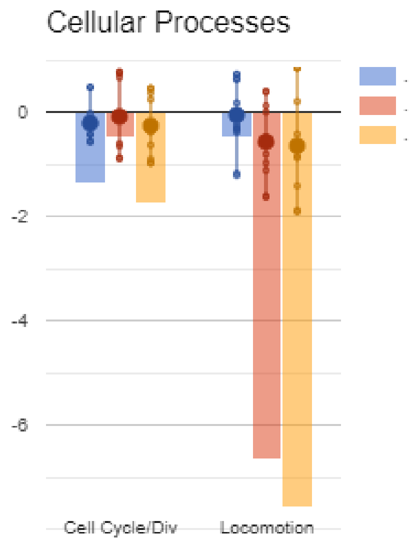

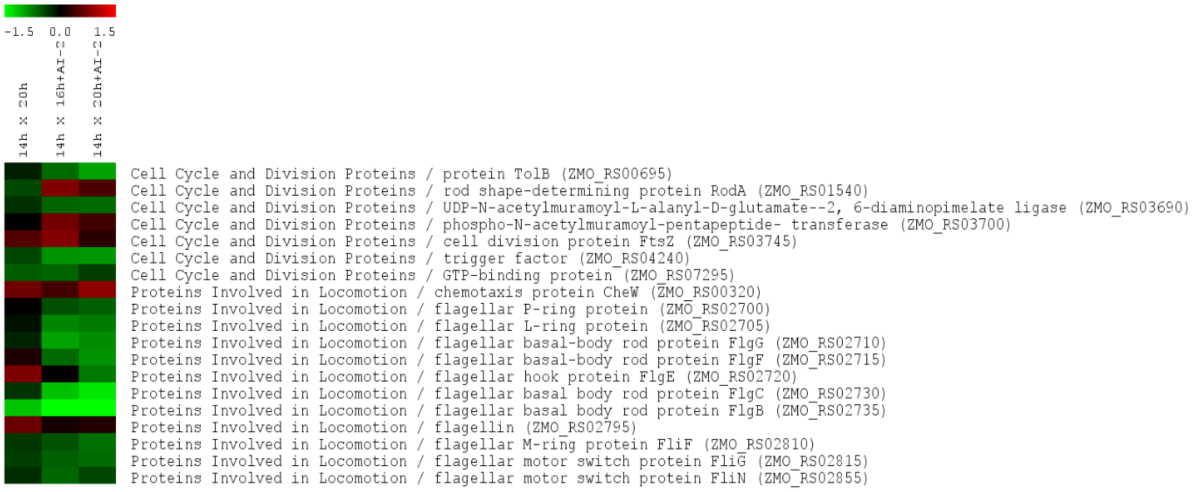

Supplementary Material S3F – Response to Stimulus

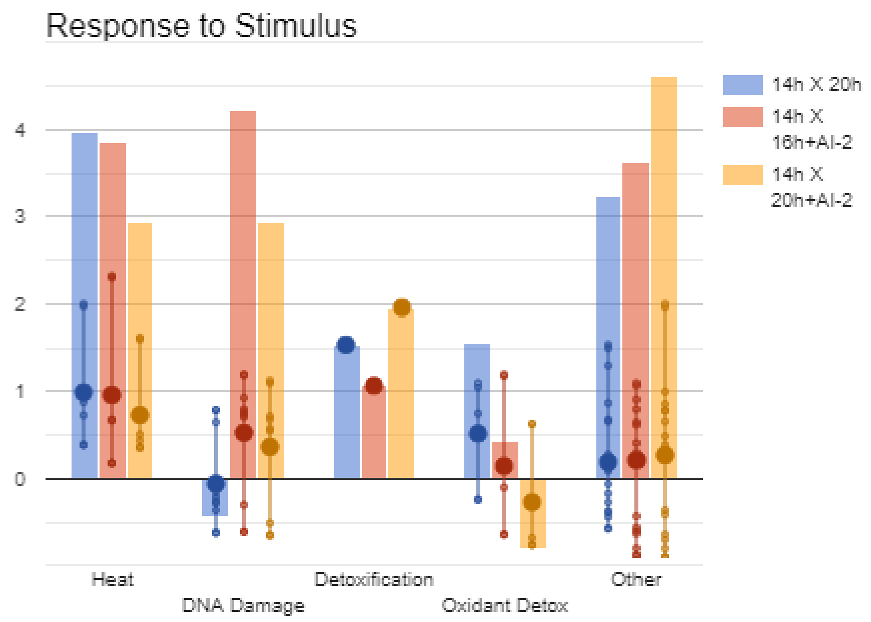

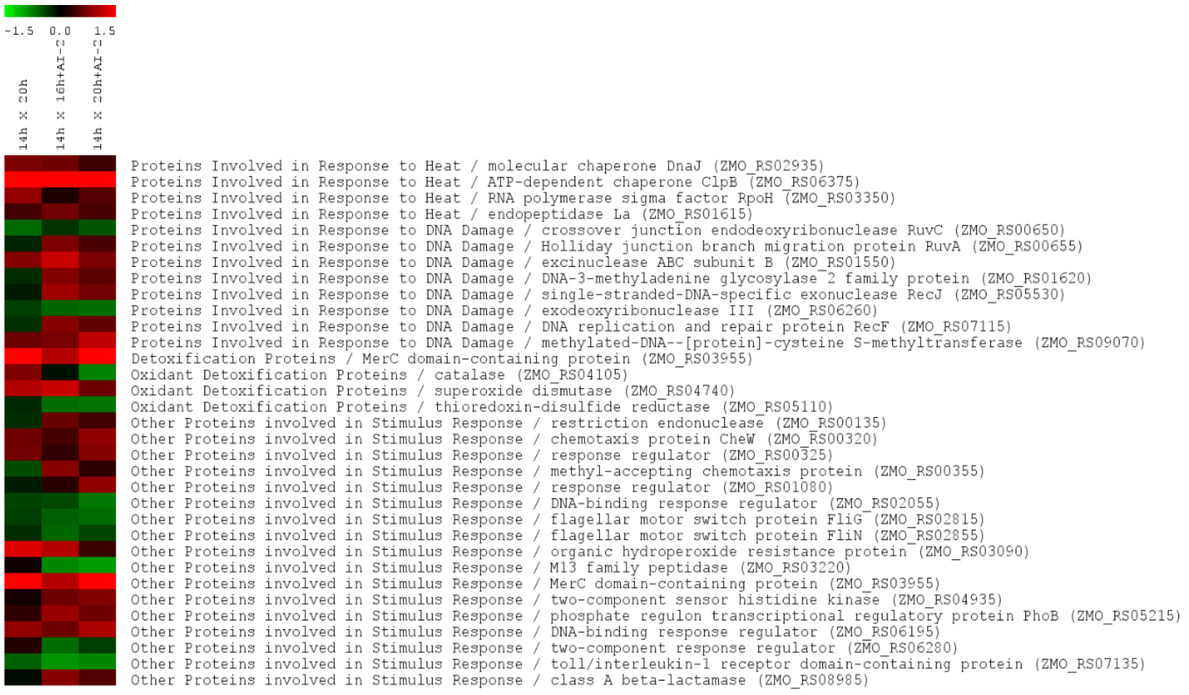

Supplementary Material S3G – Energy Metabolism

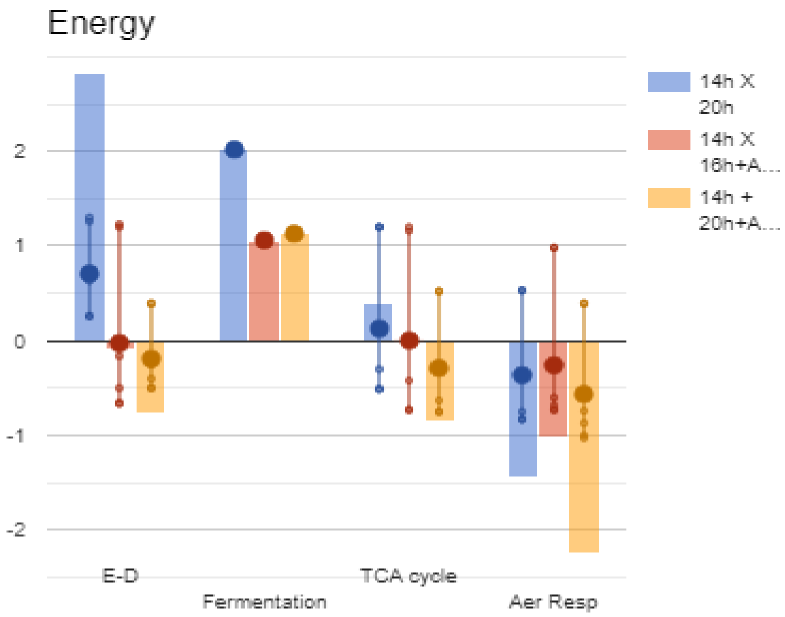

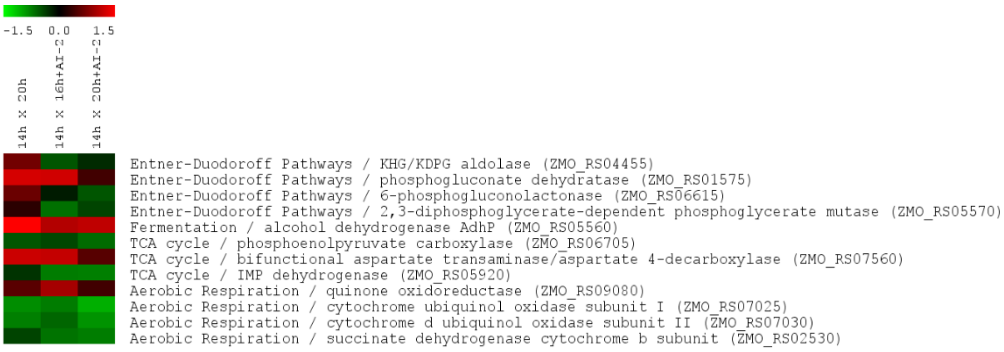

Supplementary Material S2H – Other Metabolic Pathways

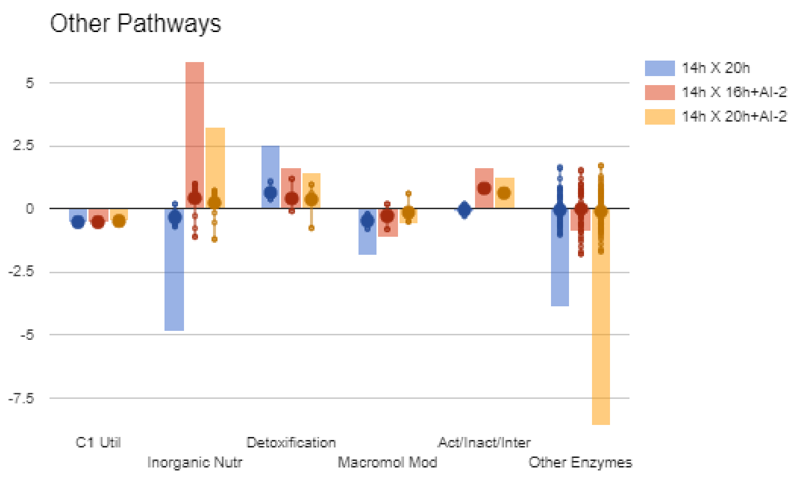

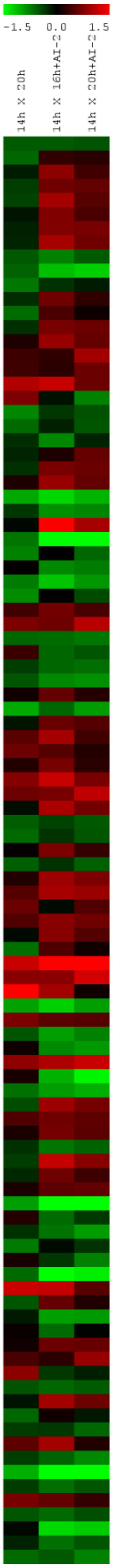

C1 Compound Utilization and Assimilation / formate--tetrahydrofolate ligase (ZMO_RS01960)
Inorganic Nutrient Metabolism / ammonia monooxygenase (ZMO_RS08530)
Inorganic Nutrient Metabolism / ammonia monooxygenase (ZMO_RS08550)
Inorganic Nutrient Metabolism / nitrogenase iron-molybdenum cofactor biosynthesis protein NifN (ZMO_RS08220)
Inorganic Nutrient Metabolism / nitrogenase iron-molybdenum cofactor biosynthesis protein NifE (ZMO_RS08215)
Inorganic Nutrient Metabolism / nitrogenase molybdenum-iron protein subunit beta (ZMO_RS08210)
Inorganic Nutrient Metabolism / nitrogenase molybdenum-iron protein alpha chain (ZMO_RS08205)
Inorganic Nutrient Metabolism / nitrogenase iron protein (ZMO_RS08200)
Inorganic Nutrient Metabolism / carbamoyl-phosphate synthase small subunit (ZMO_RS07260)
Inorganic Nutrient Metabolism / argininosuccinate synthase (ZMO_RS04635)
Inorganic Nutrient Metabolism / signal peptide peptidase SppA (ZMO_RS08750)
Inorganic Nutrient Metabolism / low molecular weight phosphotyrosine protein phosphatase (ZMO_RS00590)
Inorganic Nutrient Metabolism / acid phosphatase (ZMO_RS00545)
Inorganic Nutrient Metabolism / sulfate adenylyltransferase subunit CysN (ZMO_RS00020)
Inorganic Nutrient Metabolism / adenylyl-sulfate kinase (ZMO_RS00015)
Detoxification / lactoylglutathione lyase (ZMO_RS03400)
Detoxification / hydroxyacylglutathione hydrolase (ZMO_RS03395)
Detoxification / superoxide dismutase (ZMO_RS04740)
Detoxification / catalase (ZMO_RS04105)
Macromolecule Modification / ribonuclease D (ZMO_RS03185)
Macromolecule Modification / ribonuclease PH (ZMO_RS00070)
Macromolecule Modification / tRNA preQ1(34) S-adenosylmethionine ribosyltransferase-isomerase QueA (ZMO_RS03805)
Macromolecule Modification / leucyl/phenylalanyl-tRNA--protein transferase (ZMO_RS04150)
Activation/Inactivation/Interconversion / sulfate adenylyltransferase subunit CysN (ZMO_RS00020)
Activation/Inactivation/Interconversion / adenylyl-sulfate kinase (ZMO_RS00015)
Enzymes not in Pathways / inorganic diphosphatase (ZMO_RS06760)
Enzymes not in Pathways / DNA polymerase III subunit beta (ZMO_RS04390)
Enzymes not in Pathways / flavodoxin family protein (ZMO_RS08880)
Enzymes not in Pathways / acyl carrier protein (ZMO_RS05730)
Enzymes not in Pathways / tRNA (guanosine(46)-N7)-methyltransferase TrmB (ZMO_RS00625)
Enzymes not in Pathways / anaerobic ribonucleoside-triphosphate reductase activating protein (ZMO_RS04605)
Enzymes not in Pathways / ribonucleoside triphosphate reductase (ZMO_RS04595)
Enzymes not in Pathways / ribulose-phosphate 3-epimerase (ZMO_RS00090)
Enzymes not in Pathways / endopeptidase La (ZMO_RS01615)
Enzymes not in Pathways / aldo/keto reductase (ZMO_RS07980)
Enzymes not in Pathways / dCTP deaminase (ZMO_RS03855)
Enzymes not in Pathways / acyl carrier protein (ZMO_RS05680)
Enzymes not in Pathways / exodeoxyribonuclease III (ZMO_RS06260)
Enzymes not in Pathways / peptidyl-prolyl cis-trans isomerase (ZMO_RS02005)
Enzymes not in Pathways / DNA topoisomerase (ATP-hydrolyzing) subunit B (ZMO_RS07110)
Enzymes not in Pathways / peptidylprolyl isomerase (ZMO_RS03445)
Enzymes not in Pathways / leucyl aminopeptidase family protein (ZMO_RS03595)
Enzymes not in Pathways / quinone oxidoreductase (ZMO_RS09080)
Enzymes not in Pathways / DNA-directed RNA polymerase subunit beta' (ZMO_RS03270)
Enzymes not in Pathways / dienelactone hydrolase family protein (ZMO_RS09075)
Enzymes not in Pathways / excinuclease ABC subunit B (ZMO_RS01550)
Enzymes not in Pathways / methylated-DNA--[protein]-cysteine S-methyltransferase (ZMO_RS09070)
Enzymes not in Pathways / type I secretion system permease/ATPase (ZMO_RS01100)
Enzymes not in Pathways / DNA topoisomerase 1 (ZMO_RS05355)
Enzymes not in Pathways / crossover junction endodeoxyribonuclease RuvC (ZMO_RS00650)
Enzymes not in Pathways / DNA primase (ZMO_RS07270)
Enzymes not in Pathways / GTPase Era (ZMO_RS06180)
Enzymes not in Pathways / helicase (ZMO_RS00935)
Enzymes not in Pathways / aldo/keto reductase (ZMO_RS09055)
Enzymes not in Pathways / ribosomal large subunit pseudouridine synthase D (ZMO_RS03355)
Enzymes not in Pathways / pyruvate formate-lyase 1-activating enzyme (ZMO_RS07010)
Enzymes not in Pathways / class A beta-lactamase (ZMO_RS08985)
Enzymes not in Pathways / alkaline phosphatase family protein (ZMO_RS07200)
Enzymes not in Pathways / nitronate monooxygenase (ZMO_RS08400)
Enzymes not in Pathways / amidohydrolase (ZMO_RS01340)
Enzymes not in Pathways / NAD-dependent succinate-semialdehyde dehydrogenase (ZMO_RS07890)
Enzymes not in Pathways / ferredoxin--NADP reductase (ZMO_RS07885)
Enzymes not in Pathways / GTPase ObgE (ZMO_RS00895)
Enzymes not in Pathways / acetyl-CoA carboxylase biotin carboxyl carrier protein (ZMO_RS03290)
Enzymes not in Pathways / M13 family peptidase (ZMO_RS03220)
Enzymes not in Pathways / FAD:protein FMN transferase (ZMO_RS08290)
Enzymes not in Pathways / aldo/keto reductase (ZMO_RS06010)
Enzymes not in Pathways / ribose 5-phosphate isomerase B (ZMO_RS05385)
Enzymes not in Pathways / homocitrate synthase (ZMO_RS08260)
Enzymes not in Pathways / lytic murein transglycosylase (ZMO_RS04860)
Enzymes not in Pathways / glyoxylate/hydroxypyruvate reductase A (ZMO_RS08535)
Enzymes not in Pathways / DNA-directed RNA polymerase subunit omega (ZMO_RS07745)
Enzymes not in Pathways / glycerate kinase (ZMO_RS00250)
Enzymes not in Pathways / 16S rRNA (uracil(1498)-N(3))-methyltransferase (ZMO_RS06955)
Enzymes not in Pathways / DNA topoisomerase IV subunit B (ZMO_RS01770)
Enzymes not in Pathways / tRNA (guanosine(37)-N1)-methyltransferase TrmD (ZMO_RS04820)
Enzymes not in Pathways / uracil-DNA glycosylase (ZMO_RS05310)
Enzymes not in Pathways / ribosomal RNA small subunit methyltransferase H (ZMO_RS03675)
Enzymes not in Pathways / FKBP-type peptidyl-prolyl cis-trans isomerase (ZMO_RS07705)
Enzymes not in Pathways / ATPase (ZMO_RS02985)
Enzymes not in Pathways / polyribonucleotide nucleotidyltransferase (ZMO_RS02430)
Enzymes not in Pathways / aspartate 4-decarboxylase (ZMO_RS07560)
Enzymes not in Pathways / aryl-sulfate sulfotransferase (ZMO_RS01305)
Enzymes not in Pathways / PDZ domain-containing protein (ZMO_RS01015)
Enzymes not in Pathways / histidine kinase (ZMO_RS03055)
Enzymes not in Pathways / DNA polymerase III subunit chi (ZMO_RS05860)
Enzymes not in Pathways / HD domain-containing protein (ZMO_RS06650)
Enzymes not in Pathways / aldo/keto reductase (ZMO_RS04375)
Enzymes not in Pathways / ATPase (ZMO_RS02425)
Enzymes not in Pathways / ubiquinol-cytochrome c reductase iron-sulfur subunit (ZMO_RS04275)
Enzymes not in Pathways / 16S rRNA (guanine(966)-N(2))-methyltransferase RsmD (ZMO_RS02565)
Enzymes not in Pathways / 23S rRNA (guanosine(2251)-2'-O)-methyltransferase RlmB (ZMO_RS01000)
Enzymes not in Pathways / leucyl aminopeptidase (ZMO_RS05865)
Enzymes not in Pathways / DNA polymerase III subunit delta (ZMO_RS06415)
Enzymes not in Pathways / DNA-directed RNA polymerase subunit alpha (ZMO_RS02380)
Enzymes not in Pathways / S41 family peptidase (ZMO_RS07490)
Enzymes not in Pathways / ATP-dependent Clp protease proteolytic subunit (ZMO_RS04250)
Enzymes not in Pathways / 23S rRNA (pseudouridine(1915)-N(3))-methyltransferase RlmH (ZMO_RS07480)
Enzymes not in Pathways / carbonic anhydrase (ZMO_RS05065)
Enzymes not in Pathways / 4-hydroxy-3-methylbut-2-enyl diphosphate reductase (ZMO_RS03915)
Enzymes not in Pathways / ribonuclease H (ZMO_RS07185)
