# Supplementary material for: The Quorum Sensing Auto-Inducer 2 (AI-2) Stimulates Nitrogen Fixation and Favors Ethanol Production over Biomass Accumulation in Zymomonas mobilis
Source: Int J Mol Sci. 2021 May 26;22(11):5628. doi: 10.3390/ijms22115628 (PMC8198075; doi:10.3390/ijms22115628)

Supplementary Material S4- Expression Modulation in genes involved in glucose-to-ethanol interconversion in *Z. mobilis*. The figure shows a diagram of the mechanism used by *Z. mobilis* to produce ethanol from glucose 6-phosphate. This hexose is converted to pyruvate, using the Entner-Doudoroff (ED) glycolytic pathway, followed by pyruvate decarboxylation and reduction of acetaldehyde to ethanol. The highlighted squares next to each stage of this process represent the modulation detected for the genes encoding the respective enzymes, in comparisons involving: (i) 14h X 20h; (ii) 14h X 16h+AI-2 and (iii) 14h X 20h+AI-2, respectively. Positive modulations are highlighted in red, while negative modulations are highlighted in purple (no modulation is indicated in gray). The genes are identified by their respective *Locus Tag IDs,* according to the *Z. mobilis* ZM4 genome, available in the RefSeq repository.

Alencar *et al*. (2021). The quorum sensing auto-inducer 2 (AI-2) stimulates nitrogen fixation and favors ethanol production over biomass accumulation in *Zymomonas mobilis*. *Int. J. Mol. Sci. Submitted.*

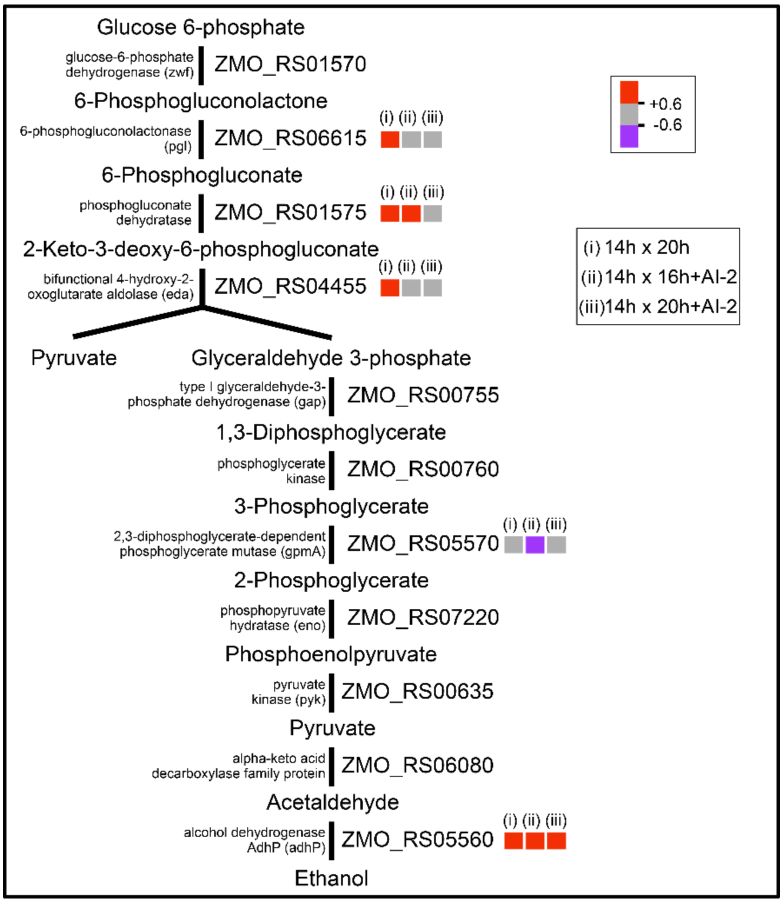

Supplement: Supplementary file 1 [file ijms-22-05628-s001.zip › Supplementary/Supplementary Material S4.pdf]
