# Supplementary material for: The Quorum Sensing Auto-Inducer 2 (AI-2) Stimulates Nitrogen Fixation and Favors Ethanol Production over Biomass Accumulation in Zymomonas mobilis
Source: Int J Mol Sci. 2021 May 26;22(11):5628. doi: 10.3390/ijms22115628 (PMC8198075; doi:10.3390/ijms22115628)
Supplement: Supplementary file 1 [file ijms-22-05628-s001.zip › Supplementary/Supplementary Material S5.pdf]

Supplementary Material S5- Primers used for qPCR of randomly selected genes.

Alencar *et al*. (2021). The quorum sensing auto-inducer 2 (AI-2) stimulates nitrogen fixation and favors ethanol production over biomass accumulation in *Zymomonas mobilis*. *Int. J. Mol. Sci. Submitted.*

LocusTag_ID	Description	Forward primer	Reverse primer
**ZMO_RS00220**	alpha/beta hydrolase	GCGATTGTCGTCGCTTTATC	CCGATCAGATGTTGCCATTG
**ZMO_RS05560**	alcohol dehydrogenase	GTGCTTGACGGTATCGAAGT	GCTTGGTGACTTTCGGTTTG
**ZMO_RS08755**	co-chaperonin GroES	GACAGCTGGCGGTATCATTA	TTCGCTATGGGTTCCGTTAC
**ZMO_RS00205**	lysine exporter protein	GGTGTTGCTTCGACTTACCT	GAAGGCTGCAAGGGAGTAAT
**ZMO_RS08485**	glutaredoxin-like protein	CCGGATCAAAGAAGTTCTGG	CCGCATTCAGAATAGAGACG
**ZMO_RS05980**	LysR family transcriptional regulator	CTTGCGGATTCAGCTTTACG	CGACCAATGCGATCGAGATA
**ZMO_RS04025**	TonB-dependent receptor	ACGCGGTGGTTTCTTCTT	TGTCGTGCTCGTCTGATTAC
**ZMO_RS00175**	sigma 54 modulation protein/ribosomal protein S30EA	GCCTCTGTCACTTTCGGTAATC	GCATGGGCGTCTTTGTCTAT
**ZMO_RS07220**	phosphopyruvate hydratase	TTATAAGCCGGGCGAAGATG	GTTTACGGCCTTCACCCTTA
**ZMO_RS02370**	30S ribosomal protein S13	CCCGACAAACAAACGTGTAG	TCAGAAAGATCCTGGACACG
**ZMO_RS06270**	peptidase M23	CCGTTATGCCGTTGCTCATA	TCGCGATTGATGGGTTCTTC
**ZMO_RS04470**	5-methyltetrahydropteroyltriglutamate-homocysteine S-methyltransferase	CAAAGCGTCAGAGCCCTTAT	AATGGTCGTCGTCGGATAGA
**ZMO_RS06320**	MucR family transcriptional regulator	ATCTGCCTCGAAGACGGTAA	TTTAGCGCGGTATTGTTCCG
**ZMO_RS02245**	elongation factor G	CCACAACCGAGCGTATTCTT	TGTTCCTGCTCCATCCAATC
**ZMO_RS00755**	glyceraldehyde-3-phosphate dehydrogenase, type I	CTGACCGCAGATGACAAGAT	CTGTGAACGGTGGTCATCAA
**ZMO_RS03200**	transcription elogation factor GreB	CCAAGGCAGAATAAGCTGGA	CTGGTAGGCGGACAATTCTAAG
**ZMO_RS04395**	ABC transporter-like protein	CTTGGAACAGGTGGGACTTT	TGGCTGCCCTTTGTCTTT
**ZMO_RS01520**	rod shape-determining protein MreB	GAAAGTGCATGGCGGTAAAG	CGAATAGCGCGTCTTTCAAC
**ZMO_RS08860**	PhzF family phenazine biosynthesis protein	CGAATGCAAGGCACTGAAAG	AAGGTACGCACACGGATT
**ZMO_RS01575**	phosphogluconate dehydratase	TTGGCTACCAGCGTTTCT	CAACAGACCAGGGACAATCT
**ZMO_RS03720**	UDP-N-acetylmuramate-L-alanine ligase	TCGGGATAGACGGCTGATTA	AGCGGTTACCACCAGAGATA
